# The epidemiology, clinical burden, and prevention of intrauterine adhesions (IUAs) related to surgically induced endometrial trauma: a systematic literature review and selective meta-analyses

**DOI:** 10.1093/humupd/dmaf019

**Published:** 2025-09-07

**Authors:** Malcolm G Munro, Christina A Salazar, Bala Bhagavath, Mark H Emanuel, Heather G Huddleston, Dhruv Sobti, Ajit K Jaiswal, Rachel Gamburg, Jatinder Kumar, Coby Martin, Angelo B Hooker, Women’s Health Research Collaborative’s Endometrial Trauma Group, Malcolm G Munro, Christina A Salazar, Bala Bhagavath, Mark H Emanuel, Heather G Huddleston, Dhruv Sobti, Ajit K Jaiswal, Rachel Gamburg, Jatinder Kumar, Coby Martin, Angelo B Hooker

**Affiliations:** Department of Obstetrics & Gynecology, David Geffen School of Medicine at UCLA, University of California Los Angeles, Los Angeles, CA, USA; Women’s Health Research Collaborative, New York, NY, USA; Women’s Health Research Collaborative, New York, NY, USA; Department of Women’s Health, University of Texas at Austin Dell Medical School, Austin, TX, USA; Women’s Health Research Collaborative, New York, NY, USA; Division of Reproductive Endocrinology and Infertility, Department of Obstetrics & Gynecology, University of Wisconsin-Madison, Madison, WI, USA; Women’s Health Research Collaborative, New York, NY, USA; Department of Gynecology and Reproductive Health, University Medical Center, Utrecht, The Netherlands; Women’s Health Research Collaborative, New York, NY, USA; Division of Reproductive Endocrinology and Infertility, Department of Obstetrics, Gynecology & Reproductive Sciences, The University of California, San Francisco, San Francisco, CA, USA; Axtria India Pvt Ltd., Gurugram, Haryana, India; Axtria India Pvt Ltd., Gurugram, Haryana, India; Axtria Inc, Berkeley Heights, NJ, USA; Axtria India Pvt Ltd., Gurugram, Haryana, India; Axtria Inc, Berkeley Heights, NJ, USA; Women’s Health Research Collaborative, New York, NY, USA; Department of Obstetrics and Gynaecology, Zaans Medical Center (ZMC), Zaandam, The Netherlands

**Keywords:** intrauterine adhesions, endometrial trauma, hysteroscopy, placenta accreta spectrum, myomectomy, metroplasty, septum removal, retained products of conception, postpartum hemorrhage, infertility

## Abstract

**BACKGROUND:**

Reproductive-age women with intrauterine adhesions (IUAs) following uterine surgery may be asymptomatic or may experience light or absent menstruation, infertility, preterm delivery, and/or peripartum hemorrhage. Understanding procedure- and technique-specific risks and the available evidence on the impact of surgical adjuvants is essential to the design of future research.

**OBJECTIVE AND RATIONALE:**

While many systematic reviews have been published, most deal with singular aspects of the problem. Consequently, a broadly scoped systematic review and selective meta-analyses identifying evidence strengths and gaps are necessary to inform future research and treatment strategies.

**SEARCH METHODS:**

A systematic literature review was performed seeking evidence on IUA incidence following selected uterine procedures and the effectiveness of hysteroscopic adhesiolysis on menstrual, endometrial, fertility, and pregnancy-related outcomes. An evaluation of the impact of surgical adjuvants designed to facilitate adhesion-free endometrial repair was included. Searches were conducted in the PubMed, Embase, and Cochrane databases following PRISMA guidelines and included English-language publications from inception to 8 November 2024. Inclusion criteria restricted articles to those reporting IUA epidemiology or related clinical outcomes. Risk of bias assessment used the US NIH tools for interventional and observational studies. Meta-analyses were conducted and reported only for outcomes where there were sufficient data. Per analysis, we report on proportions (with 95% CI), heterogeneity (*I^2^*), and the risk of bias for each study included.

**OUTCOMES:**

The review identified 249 appropriate publications. The risks of new-onset IUAs following the removal of products of conception after early pregnancy loss, hysteroscopic myomectomy, and hysteroscopic metroplasty for septum correction were 17% (95% CI: 11–25%; 13 studies, *I*^2^ = 87%, poor to good evidence quality), 16% (95% CI: 6–28%; 8 studies, *I*^2^ = 93%, fair to good evidence quality), and 28% (95% CI: 13–46%; 8 studies, *I*^2^ = 91%, fair to good evidence quality), respectively. For primary IUA prevention with adjuvant intrauterine gel barriers, the relative risks were 0.45 (95% CI: 0.30–0.68; three studies, *I*^2^ = 0%, poor to good evidence quality), 0.38 (95% CI: 0.20–0.73; three studies, *I*^2^ = 0%, fair evidence quality), and 0.29 (95% CI: 0.12–0.69; three studies, *I*^2^ = 0%, fair to good evidence quality), respectively, following the above potentially adhesiogenic procedures. Following adhesiolysis without adjuvants, the IUA recurrence rate was 35% (95% CI: 24–46%; 13 studies, *I*^2^ = 95%, poor to good evidence quality), similar to the rate of 43% for both those treated adjuvantly with an intrauterine balloon (95% CI: 35–51%; 14 studies, *I*^2^ = 85%, poor to good evidence quality), or an IUD (95% CI: 27–59%; four studies, *I*^2^ = 85%, fair to good evidence quality). The recurrence rate for secondary prevention with gel barriers was 28% (95% CI: 4–62%; three studies, *I*^2^ = 94%, good evidence quality). Notably, there was an excess rate of associated adverse obstetrical outcomes, including preterm delivery, placenta accreta spectrum, placenta previa, peripartum hemorrhage, and hysterectomy, with evidence demonstrating the beneficial impact of adjuvant therapies on these outcomes.

**WIDER IMPLICATIONS:**

This systematic review comprehensively analyzes IUA formation following uterine surgical procedures and adjuvant therapy effectiveness. Even following adhesiolysis, it is apparent that the basilar endometrial trauma thought to facilitate the formation of IUAs may persist and contribute to adverse reproductive outcomes. Many critical gaps remain in our knowledge of the pathogenesis, prevention, and management of endometrial trauma and IUAs.

**PREGISTRATION NUMBER:**

PROSPERO (ID: CRD42023366218).

## Graphical abstract

**Figure:**
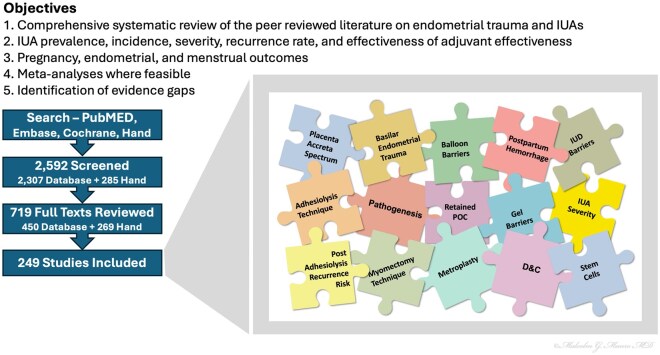
**Systematic review and selected meta-analyses of intrauterine adhesions related to endometrial trauma.** IUA, intrauterine adhesions.

## Introduction

Intrauterine adhesions (IUAs) are a manifestation of damage to the basilar layer of the endometrium that results in variable degrees of endometrial fibrosis. The adhesions present as bands of fibrous scar tissue that bind the surfaces of the uterine cavity, which comprises the endometrial cavity and cervical canal. In severe cases, the endometrial cavity can be completely obliterated with no underlying functional endometrium. The disorder was first described by Heinrich Fritsch in 1894 (Fritsch, 1894), then by Bass in 1927 (Bass, 1927), and Stamer in 1946 (Stamer, 1946), before Joseph Asherman published the two papers, in 1948 and 1950, which led to the eponymous syndrome known today (Asherman, 1948, 1950). Indeed, Asherman’s disease is now considered synonymous with IUAs, while the term Asherman’s syndrome should be used when IUAs are found in association with symptoms that may include infertility, recurrent pregnancy loss, and light menstrual bleeding or amenorrhea (Yu *et al.*, 2008b).

While typically caused by transcervical surgical procedures that injure the basilar endometrial layer, such as dilation and curettage (D&C), IUAs may also occur secondary to other uterine interventions such as abdominal myomectomy performed either laparoscopically or via laparotomy (Capmas *et al.*, 2018; Bortoletto *et al.*, 2022; Lagana *et al.*, 2022), image-guided procedures for leiomyomas and adenomyosis such as uterine artery embolization (Toguchi *et al.*, 2020; Wang *et al.*, 2020a), or even intra-abdominal procedures performed at the time of Cesarean delivery designed to treat postpartum hemorrhage (Zouaghi *et al.*, 2023). Genital tuberculosis is a common cause of severe endometrial trauma and IUAs in endemic areas, specifically Africa and Asia, where it is estimated to comprise 20% of the etiologies of infertility cases (Sharma *et al.*, 2008; Sharma *et al.*, 2021). In the developed world, including the USA and Europe, it is more common in immigrants from endemic areas but overall appears to be involved in <1% of women with infertility (Langer *et al.*, 2019; Sharma *et al.*, 2021).

Whereas minimal endometrial trauma and mild IUAs can be asymptomatic, more extensive trauma and associated severe IUAs are a known cause of light or absent menstruation (amenorrhea), in addition to other manifestations such as infertility, recurrent pregnancy loss, and inability to access the endometrial cavity for diagnostic testing (Salazar *et al.*, 2017). Even when IUAs are treated surgically, recurrence is common, and regardless, the underlying trauma to the endometrial basalis may not be corrected, and the functional endometrium may not be fully restored. When pregnancy occurs following treatment of IUAs, there is evidence of adverse obstetrical outcomes, including preterm delivery and postpartum hemorrhage (Deans *et al.* 2018; Hui *et al.*, 2018; Hooker *et al.*, 2020). A consequence of endometrial trauma may be impaired placental circulation, increasing the risk of growth restriction and preterm labor (Hooker *et al.*, 2022). The absence of an intact decidualized endometrial basalis can lead to abnormal placental attachment to, or invasion of, the myometrium, a circumstance known as placenta accreta spectrum (PAS) disorder, and is associated with increased risks of peripartum hemorrhage and requires additional interventions, including blood transfusion, uterine artery occlusion, and hysterectomy (Deans *et al.*, 2018; Feng *et al.*, 2020; Zhang *et al.*, 2020; Tavcar *et al.*, 2023). These circumstances may result in substantial maternal and neonatal morbidity and related increased utilization of health care resources.

The initial approach to primary prevention of IUAs is the avoidance of endometrial infection and potentially traumatic intrauterine surgical procedures. However, in many cases, such procedures are necessary for optimal care. Consequently, it could be hypothesized that adopting surgical techniques designed and executed in fashions that minimize basilar endometrial trauma should reduce the risk of associated adhesions. In addition, following a potentially traumatic and adhesiogenic procedure, utilizing systemic and/or locally applied adjuvant therapies, including systemic pharmaceuticals, intrauterine barriers, and active intrauterine agents such as ‘biologics’, may facilitate optimal basilar endometrial repair without adhesion formation.

For women diagnosed with IUAs, hysteroscopically directed adhesiolysis is considered the treatment of choice. The measures taken to optimize basilar endometrial repair, thereby reducing the risk of reformation of IUAs, can collectively be called secondary prevention and, in addition to minimally traumatic dissection, include the adjuvant therapies already described as potentially applicable for primary prevention.

This systematic review and, where feasible, meta-analyses, synthesize the body of evidence examining the incidence of IUAs following potentially adhesiogenic uterine surgical procedures and the impact of the various techniques and adjuvants used for the primary and secondary prevention of IUAs. We also analyzed secondary surgical outcomes, such as the thickness of the endometrial echo complex (EEC), often referred to as endometrial thickness (EMT), and the impact on menstrual function as well as pregnancy outcomes, including adverse obstetrical and neonatal outcomes, such as preterm delivery, placenta previa, peripartum hemorrhage, and the related PAS disorder. Our scope did not include IUAs related to image-guided procedures, intraoperative procedures related to the treatment of peripartum hemorrhage at Cesarean section, or to infectious disease, in particular, tuberculosis. A glossary of the definitions and terms used in this review is found in Table 1.

**Table 1.** Glossary of terms.

Abdominal myomectomy	Removal of leiomyomas, usually from the uterus, performed abdominally either via laparotomy or under laparoscopic direction. Laparoscopic myomectomy may be performed with or without the use of an assistive device (e.g. the da Vinci^®^ system).
Abnormal uterine bleeding (AUB)	Non-gestational vaginal bleeding of uterine origin in the reproductive years that is abnormal in frequency, regularity, duration, and/or volume that includes bleeding between periods and unscheduled bleeding associated with the use of medications or intrauterine contraceptive devices.
Bipolar RF instrument	A radiofrequency (RF) electrosurgical instrument that contains both electrodes. During hysteroscopic surgery, this design allows function in electrolyte-containing distention media.
Cervical canal	The anatomical passage through the cervix, lined with columnar epithelium, connecting the vagina to the endometrial cavity.
Cervix	See Uterine Cervix.
Dilation and curettage (D&C)	A procedure involving serial expansion of the cervical canal using a set of dilators (dilation) followed by inserting a serrated instrument for blind mechanical removal of the endometrium, intracavitary lesions, and/or products of conception. Alternatively can also be performed using suction curettage for evacuation of uterine contents.
Electromechanical tissue removal instrument	A usually proprietary cylindrical hollow hysteroscopic instrument with a distal side fenestration surrounding an oscillating cylinder with an aligned fenestration that acts as a blade. When activated, tissue is drawn through the fenestration with suction where it is transected (morcellated) by the blade. The morcellated tissue is generally aspirated by suction to a remotely-located tissue trap.
Endometrial cavity	The potential space within the uterine corpus, lined with endometrium, is connected at the lateral cornua to each fallopian tube and caudally to the cervical canal. It comprises the largest component of the ‘uterine cavity’.
Endometrial echo complex (EEC)	The endometrium and its contents, if any, as seen typically on the sagittal view with transvaginal ultrasound and sometimes referred to as the ‘endometrial stripe’. It is measured from the endomyometrial interface anteriorly to the same interface posteriorly and includes any content visualized within the cavity between the endometrial layers.
Endometrial thickness (EMT)	The double layer of endometrium visualized sonographically as the EEC but absent any endometrial cavity content. It is generally used as a metric in infertility care and investigation of abnormal uterine bleeding.
Endometrial trauma	Damage to the endometrium, typically from surgical procedures. When this trauma damages the basilar layer of endometrium, it can result in the formation of intrauterine adhesions. Such trauma and enduring damage can also occur secondary to a chronic endometrial infection such as tuberculosis.
Endometrium	The layer of glands and stroma that lines the endometrial cavity. Receives and supports the early development of a pregnancy and, absent pregnancy, the superficial component (functionalis) partially sloughs during the process of menstruation.
Hysteroscopy/hysteroscopic	An endoscopic technique used to visually direct the performance of intrauterine diagnostic or operative procedures with instruments passed through the cervical canal.
IVF-ET	In vitro fertilization (IVF) and embryo transfer (ET). IVF refers to fertilization of an egg with sperm in a laboratory setting, while ET is the placement of a resulting embryo into the endometrial cavity via a catheter inserted through the cervical canal.
Intrauterine adhesions (IUAs)	Fibrous tissue abnormally attaching opposing sides of the endometrial cavity and/or cervical canal secondary to abnormal healing typically following basilar endometrial trauma.
Laparoscopy/laparoscopic	An abdominal endoscopic (telescopic) technique used to visually direct the performance of intraperitoneal diagnostic or operative procedures with instruments passed through small, ancillary ports.
Laparotomy/laparotomic	An intraperitoneal procedure performed through an abdominal incision large enough for the passage of the hand and/or standard surgical instruments.
Hysteroscopic metroplasty	Surgical (usually hysteroscopic) correction of congenital anomalies (Müllerian) of the uterus, most commonly of a septum, but may include remodeling for ‘T-shaped’, ‘Y-shaped’, or other dysmorphic congenital anomalies.
Monopolar RF instrument	A radiofrequency (RF) electrosurgical instrument that contains only one small diameter electrode designed to create the surgical tissue effect; the second is a large area electrode positioned remotely, typically on the thigh, designed to disperse the current preventing damage to the skin. During hysteroscopy, this design requires electrolyte-free distention media to function.
Myomectomy	Removal of a leiomyoma, almost invariably from the uterus. Myomectomy may be performed hysteroscopically (hysteroscopic myomectomy), vaginally (vaginal myomectomy), or abdominally (abdominal myomectomy via either laparotomy (laparotomic myomectomy) or under laparoscopic direction (laparoscopic myomectomy). Laparoscopic myomectomy may be performed with or without the use of an assistive device (e.g. the da Vinci® system).
Opposing leiomyomas	Submucous leiomyomas (e.g. FIGO Type 0, 1, or 2) situated adjacent to each other on opposite sides of the endometrial cavity.
Primary prevention (of IUAs)	Adjunctive surgical techniques, devices, medications, or other prophylactic interventions designed to facilitate endometrial repair following surgically induced endometrial trauma, thereby reducing or eliminating the risk of IUA formation. Primary prevention can also be considered to encompass avoidance of such procedures using expectant, medical or surgical management techniques.
Radiofrequency (RF) electrical energy	High frequency (300–500K Hz), alternating polarity electrical current used to create tissue effects (vaporization or coagulation) between two electrodes by rapid elevation of intracellular temperature.
Retained products of conception (RPOC)	Pregnancy tissue not completely expelled at the time of spontaneous miscarriage or delivery, or remaining in the endometrial cavity following attempted medical or surgical removal.
Second look hysteroscopy (SLH)	Refers to a repeat diagnostic hysteroscopic evaluation typically performed 4–12 weeks after a surgical procedure that may adversely impact the endometrium. The procedure is used to assess for the presence of IUAs or, following adhesiolysis, recurrent intrauterine adhesions. If found, adhesiolysis is typically performed during the same procedure.
Secondary prevention (of IUAs)	Adjunctive surgical techniques, devices, or other therapeutic and prophylactic interventions designed to be used in association with intrauterine adhesiolysis, thereby reducing or eliminating the risk of recurrent formation of IUAs.
Sonohysterography (SHG)	The performance of uterine ultrasound while instilling and distending the endometrial cavity with sonolucent contrast, usually gel or normal saline (also commonly referred to as gel infusion sonography ‘GIS’ or saline infusion sonography ‘SIS’). This imaging technique enhances the visualization of intracavitary lesions such as polyps, leiomyomas, and adhesions.
Uterus	The normally pear-shaped, muscular reproductive organ in the female pelvis comprising a corpus (body) and the cervix. It supports a pregnancy until maturity when its muscular function results in expulsion of the fetus through a fully dilated cervical canal.
Uterine cavity	The lumen within the uterus comprising the endometrial cavity and the contiguous cervical canal.
Uterine cervix	The fibromuscular cylindrical portion of the uterus connecting the uterine corpus to the vagina that contains the cervical canal.
Uterine corpus	The muscular ‘body’ of the uterus that is comprised of myometrium, surrounding serosa, and the roughly T or triangular-shaped endometrial cavity lined with endometrium. The uterine corpus attaches inferiorly to the uterine cervix and laterally, at the cornua, to the right and left fallopian tubes.

## Methods

### Systematic review protocol and registration

This systematic literature review (SLR) was reported using the Preferred Reporting Items for Systematic Reviews and Meta-Analyses (PRISMA) statement (Page *et al.*, 2021) and conducted in alignment with the Cochrane Handbook for Systematic Reviews of Interventions (Higgins *et al.*, 2011). The study protocol was registered in the International Prospective Register of Systematic Reviews database (PROSPERO ID: CRD42023366218).

### Search and study selection

An extensive systematic search of the English language literature regarding surgically induced IUAs was conducted using the PubMed, Embase, and Cochrane Library databases. The original search was run from inception to 16 December 2022, with no search filters applied for geographical location. A subsequent search of all three databases was performed on 8 November 2024, to capture subsequent candidate publications. Search strategies were developed using the Medical Subject Headings (MeSH) terms and relevant keywords pertaining to the patient population, intervention, and the outcomes of interest. The search strategies, including all search terms, are provided in Supplementary Tables S1 and S2. Additionally, the bibliographies of relevant SLRs identified in the search were hand-searched for potentially relevant publications.

### Screening process

Studies identified in the database searches were reviewed by two independent reviewers at the title and abstract stage to be selected for full-text review. Discrepancies were resolved by consensus and, if necessary, by a third independent reviewer and confirmed by the principal investigator (M.G.M.). Full-text review was also performed by both reviewers, with discrepancies generated and reconciled similarly by consensus, or if necessary, by the principal investigator. Studies identified through hand searches were immediately advanced to the full-text screening stage. The screening process was conducted based on predefined eligibility criteria for the study population, intervention, comparator, and study design (PICOS criteria).

The inclusion criteria, according to the PICOS framework, for the selection of studies were (i) published in English; (ii) population: pre-menopausal women of reproductive age; (iii) interventions: any intrauterine or abdominal surgical procedure that could affect the uterus, including the endometrial cavity, such as hysteroscopic or abdominal myomectomy by laparotomy or laparoscopy; in addition, all adjuvant treatments used in surgeries were considered eligible, as were all surgical tools; (iv) comparator: no specific comparator was required; (v) outcomes: IUA rates and severities, pregnancy-related outcomes, and menstrual outcomes; and (vi) study design: studies with sample sizes of <10 patients were excluded, as were narrative reviews, editorials, letters, opinion pieces, conference abstracts, and other systematic reviews.

The studies included following the full-text review were extracted by two independent reviewers into a Microsoft Excel-based (Microsoft Corp, Redmond, Washington) Data Extraction Form (DEF). The DEF comprised five major sections: (i) study details (study title, author list, citations, and geographical region); (ii) methods (study design, study duration, and follow-up duration); (iii) procedural details (surgical procedures, index procedure, diagnostic procedures, techniques used, and adjuvant therapies utilized); (iv) patient demographics (population types, age, and total population); and (v) outcomes (primary and secondary outcomes and complications). Primary outcomes included the prevalence, incidence, and recurrence rate of IUAs and adhesion severity as diagnosed by direct visualization with hysteroscopy. Secondary obstetrical outcomes included rates of natural pregnancy, pregnancy following ART, live births, and miscarriages. Secondary gynecologic outcomes included EMT and menstrual function (graded as absent, light, regular, or heavy). Additional outcomes extracted were related to obstetrical complications, including preterm delivery, PAS disorders (comprising placenta accreta, increta, and percreta), as well as placenta previa and peripartum hemorrhage.

Selected authors extracted the data, emphasizing means, medians, ranges, and risk ratios (RRs) in the observed data. Outcomes reported at multiple time points were extracted at each point so that analysis and comparisons could be made afterward. This is commonly applied to endometrial outcomes (EMT and menstrual function) and adhesion scores pre- and post-adhesiolysis at the time of second-look hysteroscopy.

### Study quality assessment

Assessment of study quality was conducted by three independent reviewers (D.S., J.K., and A.K.J.) supervised by M.G.M., using two quality assessment tools published by the National Institutes of Health (NIH, 2021), the Quality Assessment of Controlled Intervention Studies Tool and the Quality Assessment of Observational Studies Tool, which were utilized to assess controlled intervention and observational studies, respectively. Discrepancies between reviewers were resolved with discussion, and if a consensus was not reached, a third reviewer was consulted. In this assessment, the quality of each included study was rated as good, fair, or poor. Within each tool, the risk of bias (RoB) assessment was based on 14 items, with possible responses of ‘Yes’, ‘No’, ‘Cannot Determine’, ‘Not Applicable’, ‘Not reported’, and ‘None of the above’. Records that received a ‘Yes’ for 11–14 items were categorized as ‘Good’, whereas those that scored 7–10 were deemed ‘Fair’, and finally, those that scored 1–6 were deemed ‘Poor’.

### Evidence synthesis

Following data extraction, the available evidence within the literature was reviewed to determine which outcomes represented targets for meta-analysis or summary statistics as forms of evidence synthesis. Meta-analysis was preferably conducted on primary outcomes related to the risk of IUA formation, which was the focus of this review. The feasibility of meta-analysis of each outcome was determined according to the similarity among populations and study designs. For outcomes wherein comparisons were made, meta-analyses were limited to subsets of the included studies with head-to-head randomized controlled trial (RCT) designs. These included the efficacy of preventative adjuvants in reducing the incidence or recurrence of IUAs. For other outcomes, wherein no comparison was intended, or this comparison could not be informed using controlled trials due to gaps in the literature, observational studies were considered acceptable study designs for inclusion within a meta-analysis. Due to population and study design differences, meta-analysis results are presented without comparison between interventions. Finally, if results were not reported in enough detail or among publications not sufficiently similar in population or study design, these were summarized and presented descriptively herein.

Following the rationale mentioned above, comparative meta-analyses were conducted to analyze both the incidence and recurrence of IUAs in patients who did and did not receive preventative adjuvant treatments for primary and secondary IUA prophylaxis. These analyses were informed using the results of controlled interventional studies with similar populations and study designs. Other meta-analyses were conducted using a combination of observational and randomized control study results, including IUA-related outcomes and those related to pregnancy, menstrual function, and EMT.

### Meta-analysis methodology

All statistical analyses were conducted using R software version 4.2.2 (Core, 2024). A single-arm meta-analysis was performed for effect measures calculated by counting the number of individuals who experienced an event. In these cases, results were reported by the included studies as proportions, representing the proportion of patients experiencing a given event. This method was applied to analyze the prevalence, incidence, and recurrence of IUAs. We used an inverse-variance method, a random effect model, to derive a pooled estimate for the outcomes. Using this methodology, each study was weighted based on the inverse of the variance of the effect estimate. Thus, studies with large sample sizes and smaller SEs were given more weight than those with small sizes and high SEs, enhancing the pooled estimate’s precision (Deeks *et al.*, 2023).

To compare the effect of different interventions on outcomes, such as incidence and recurrence of IUAs, the proportion of patients experiencing an event (IUAs) in each arm (intervention type) was meta-analyzed comparatively to generate RRs. The pooled RR was calculated using the Mantel–Haenszel method, which derives the study weights using the number of events and non-events in the treatment and control groups (Harrer *et al.*, 2021). This random effect model was preferred as it provides greater statistical precision compared with other available methods when binary events are analyzed (Deeks *et al.*, 2023).

Finally, a standardized mean difference (SMD) was used as an effect measure for outcomes that evaluated continuous data. A SMD was used to combine the mean adhesion scores that were calculated using various scoring algorithms and EMT measurements. We used the Hedges’ g statistic for SMDs, as our analysis was conducted over a relatively small sample, and the studies had greater sample size variations in the experimental and the control arms (Harrer *et al.*, 2021). The inverse variance approach was used to calculate the study weights in the pooled estimates. Given a high degree of heterogeneity among the included studies regarding the study design, effect measures, sizes, study population, and study interventions, a random effects model was used for the meta-analyses of all outcomes. Pooled outcomes from the analyses were reported along with their corresponding 95% CIs, with results presented as a forest plot. Additionally, the degree of heterogeneity was presented using *I*^2^ and τ^2^ statistics in each forest plot.

### Literature summary through meta-analysis

#### Standardized exploration methodology

Standardized inclusion criteria were implemented for each outcome analyzed to ensure that studies were sufficiently homogenous and of sufficient quality for use in meta-analysis. These comprised a set of PICOS criteria for each meta-analysis and were based on study features, including population (generally the study patients’ disease or disorder), intervention (surgical technique or IUA-preventative adjuvant treatment), comparison made (if any), outcome reported and metrics used, and study design (RCTs or any design). Some criteria implemented stemmed from the understanding that RCTs represent the highest level of evidence for comparative effectiveness studies, allowing comparison of treatment outcomes amongst otherwise homogeneous groups of subjects. Therefore, meta-analyses of comparative effectiveness were limited to RCTs with direct head-to-head comparisons of the interventions of interest. The intended comparisons were between surgical techniques, tools, and adjuvant treatments designed to prevent or reduce the rate or severity of IUA formation. The rigorous nature of RCTs also provides consistency within treatment arms and standardized subject evaluations to yield more reliable conclusions. Where heterogeneity between studies was a concern, meta-analyses were limited to RCTs as a data set. These included the proportional meta-analyses of single arms of studies of IUA recurrence following adhesiolysis, which focused on surgical techniques or adjuvant treatments without comparisons.

The proposed study set for analysis was restricted to subjects who had undergone a diagnostic hysteroscopy capable of yielding an IUA diagnosis. This criterion was imposed to ensure all patients with IUAs could be identified, minimizing false negatives. Studies were excluded from analyses if IUAs were diagnosed using any method other than hysteroscopy. Studies were grouped according to the population studied, thereby facilitating meta-analysis. Pooled prevalence estimates were to be derived using a proportional meta-analysis by combining the findings from single-arm studies. We also sought to determine the prevalence of IUAs by severity, grouped categorically into ‘mild’, ‘moderate’, or ‘severe’ adhesions, and by reported mean/median adhesion scores (if provided).

##### Prevalence of IUAs

We sought to determine the background prevalence of IUAs among the general population and at-risk groups who had reportedly not undergone previous uterine surgeries.

#### Incidence and severity of IUAs following potentially adhesiogenic procedures and comparative efficacy of adjuvant therapies

The incidence of IUAs following potentially adhesiogenic uterine surgeries was estimated using single-arm proportional meta-analyses, which yielded a proportion of patients with IUAs following each procedure. These analyses were conducted separately for specific types of uterine procedures, including hysteroscopic metroplasty for septum correction, hysteroscopic myomectomy, and laparoscopic and laparotomic myomectomy. In addition, these analyses were performed for pregnancy-related procedures such as D&C for removal of products of conception (POC) following spontaneous pregnancy loss, as well as for removal of pregnancy tissue not completely expelled at the time of miscarriage or delivery or remaining in the endometrial cavity following attempted medical or surgical removal (known as retained products of conception; RPOC). Studies that evaluated patients with existing IUAs were excluded from these analyses.

Meta-analyses were further segregated according to subcategories of patients’ uterine conditions. Studies based on septum correction were also evaluated separately for those who experienced incomplete surgery, meaning less than total septal removal. Studies of patients with leiomyomas were initially subdivided according to the surgical approach to myomectomy, which could be either hysteroscopic or abdominal (laparoscopic or laparotomic). For meta-analyses, patients’ fibroid phenotypes were evaluated by the International Federation of Gynecology and Obstetrics (FIGO) classification (Munro *et al.*, 2011; Munro *et al.*, 2018), as well as other features such as leiomyoma size and number, and, when more than one, whether they were opposing or non-opposed within the endometrial cavity. Studies of patients who underwent myomectomy were further subdivided according to the technique used to perform the myomectomy, including whether radiofrequency (RF) electrosurgery with monopolar or bipolar instruments or electromechanical morcellation was utilized and whether cold-dissection techniques within the leiomyoma pseudocapsule were described. These subdivisions were applied due to the potential for these myomectomy techniques to result in different rates of IUA formation. Studies of patients who underwent D&C were subdivided according to the method used to perform the procedure and the time of pregnancy at which patients underwent surgery, categorized by pregnancy trimester.

Among these categories, meta-analyses of the incidence of IUAs were further subdivided according to study design, with analyses restricted to subsets of RCTs and non-RCTs and an unrestricted study design group. We permitted the ‘no adjuvant’ arms of the included studies to allow antibiotics and/or estrogens with or without progestins. The reasons for this decision were 2-fold. First, the available high-quality evidence suggests that these agents have no impact on measured adhesion outcomes (AAGL-ESGE, 2017; Yang *et al.*, 2022b; Hanstede *et al.*, 2023). Second, the frequency of antibiotic and estrogen preparations use was often the implied standard of care, and excluding studies that reported the use of these agents in each arm of a protocol would frequently preclude meta-analyses.

For each analysis described above, severity was also investigated using two methods for combination via meta-analysis. First, pooled incidence estimates were sought for each qualitative category of IUA severity, including mild, moderate, and severe, as described by the authors of each study. Second, mean and/or median IUA scores were sought in each standard IUA classification scale: March, European Society of Gynecological Endoscopy (ESGE), and American Fertility Society (AFS). To allow for meta-analyses of studies using different classification systems, the severities were re-categorized as mild, moderate, or severe following the protocol described by Hooker *et al.* (2014). While menstruation does not depend solely on IUA severity, given that AFS scores include menstrual outcomes, studies were investigated to determine whether menstrual outcomes were included in patients’ scores or omitted. Analyses were then conducted separately for studies that fit either category. Finally, we analyzed the relative effectiveness of various adjuvants for IUA prevention by evaluating changes in adhesion severity scores. In our review, we searched for randomized studies that evaluated the comparative effectiveness of adjuvant therapies in adhesion prevention and reducing adhesion severity. All adjuvant therapies were investigated and categorized into groups, including those that served as barrier methods, such as intrauterine devices (IUDs) and intrauterine balloons, application of biological agents (such as amnion graft, stem cells, and platelet-rich plasma), as well as dissolving or biodegradable barriers, such as intrauterine gels, mainly comprising some form of hyaluronic acid. Where estimates of comparative efficacy were not possible due to insufficient studies comparing the desired adjuvant treatments, proportional meta-analyses were conducted using single arms of each study that were treated with adjuvants within the same category.

#### Frequency and severity of IUA recurrence following adhesiolysis and comparative efficacy of adjuvant therapies

The data set for estimating the recurrence rate of IUAs comprised studies of patients who underwent hysteroscopic adhesiolysis for existing adhesions. To conduct adhesiolysis, surgeons typically use one or a combination of two types of instrumentation: ‘cold’ scissors, which use no energy source, or energy-based dissection with either monopolar or bipolar RF electrosurgical instrumentation. Because the choice of adhesiolysis technique could potentially affect the recurrence of IUAs, the plan for meta-analyses of studies evaluating recurrence was to conduct them separately according to the method used, in addition to meta-analyses of adhesiolysis using any technique. Understanding the limitations of heterogeneity inherent within surgical methodologies, we decided to optimize other aspects of study design by limiting these analyses to include RCTs only.

As executed for primary prevention of IUAs, we also attempted to meta-analyze the effectiveness of adjuvant therapies in secondary prevention of IUAs amongst patients undergoing hysteroscopic adhesiolysis. At the highest level, adjuvants were categorized as intrauterine barriers, intrauterine biologics, and systemic agents, comprising various designs, compositions, and durations of exposure or deployment. To support these meta-analyses, we searched for studies that evaluated the recurrence or severity of IUAs following adhesiolysis with the subsequent application of single or combined adjuvant therapies. For these analyses, studies were required to use an RCT design with at least two arms, comparing an adjuvant to another adjuvant, a combination of adjuvants, or no adjuvant. The resulting severity of adhesions was evaluated as described in the prior section, based on reported severity through either numerical or qualitative scales.

Early in the review process, it was evident that the vast majority of comparative studies utilized some combination of orally administered estrogen and progestin for a few months following adhesiolysis. While thought to facilitate the endometrial repair process, the rationale for this approach for those with functional ovaries and circulating levels of estradiol is unclear. It became apparent that excluding studies from analysis where the subjects in each arm received estrogen with or without progestin adjuvant therapy would severely limit our meta-analyses. While we decided to allow the use of estrogen-based hormonal adjuvants in the studies selected for our analyses, we separately looked to identify RCTs suitable for meta-analysis that compared post-surgical adhesion outcomes with and without gonadal hormonal adjuvants. We included only those that used one of the accepted IUA classification systems for adhesion severity. Where adhesion scores were not reported as a change from baseline as determined at the second-look hysteroscopy, the follow-up values were evaluated alone to assess the potential effect of adjuvant estrogens and progestins.

#### Pregnancy-related outcomes and comparative efficacy of adjuvant therapies

We sought to identify studies that reported pregnancy-related outcomes following hysteroscopic adhesiolysis. These outcomes included pregnancy rates, miscarriage, and ectopic pregnancy, as well as obstetrical outcomes, including live birth, intrauterine growth restriction, premature labor and delivery, placenta previa, peripartum hemorrhage, retained placenta, and PAS. Where appropriate, studies were meta-analyzed to estimate the proportion of patients experiencing various adverse pregnancy outcomes.

The analysis of early pregnancy-related outcomes was bifurcated into primary and secondary prevention groups. The primary prevention group included studies that evaluated patients without prior IUAs undergoing uterine surgeries with known adhesiogenic risk, including hysteroscopic myomectomy, hysteroscopic metroplasty for septum correction, and surgical management of RPOC, with and without adjuvant therapies. The secondary prevention group included studies reporting on patients with known IUAs undergoing hysteroscopic adhesiolysis with and without adjuvant therapies. We further searched for studies assessing the effect of adjuvant therapies on obstetrical outcomes. Pregnancies and live births were reported as a proportion of all patients evaluated, while miscarriages were reported as a proportion of all pregnancies. Meta-analyses were further subdivided by study design.

#### Menstrual outcomes

Patients with IUAs often present with reduced menstrual flow or amenorrhea, and surgical interventions with adhesiolysis may subsequently restore normal menstrual function (Amer *et al.*, 2010; AAGL-ESGE, 2017; Huang *et al.*, 2020b; Zhou *et al.*, 2021). Studies included in the review evaluated the effectiveness of hysteroscopic adhesiolysis and adjuvant therapies in restoring normal menstruation. They were restricted to RCTs evaluating subjects with previously identified IUAs to improve the homogeneity of the studied population. As was the case for other outcomes, we also evaluated the impact of adjuvant therapies on menstrual outcomes in patients with IUAs.

These study outcomes were typically reported at baseline (i.e. before the index surgery) and at some follow-up time. Therefore, our analyses focused on deriving the estimates of patients with restoration of normal menstruation after hysteroscopic adhesiolysis. This was calculated by deriving the mean difference between the proportion of patients achieving normal menstruation at follow-up and patients with normal menstruation at baseline. The SD for the calculated statistic was obtained using a published methodology (Berman, 2024). Studies were excluded from this analysis if they did not report on patients’ menstrual patterns, including amenorrhea, at baseline. We sought to analyze the proportion of patients with amenorrhea at the baseline who subsequently resumed normal menstruation following adhesiolysis. We also sought to analyze the proportion of patients reporting light (‘hypomenorrhea’) and infrequent (‘oligomenorrhea’) menstrual bleeding following adhesiolysis to combine findings using meta-analysis.

#### Endometrial thickness

Endometrial thickness (EMT) measures the endometrial echo complex (EEC) as assessed via ultrasound. A relatively thick EEC has been correlated with increased successful implantation and pregnancy maintenance rates (Baradwan *et al.*, 2018; Feng *et al.*, 2020). Therefore, we searched for studies investigating the change in EMT using the reported mean measurements at baseline and follow-up (Higgins *et al*., 2019). We derived the mean and SD using the methodology described in the Cochrane Handbook for studies that reported the statistics as median and interquartile range (Higgins *et al*., 2019). Finally, the calculated statistics were combined to derive the pooled SMD in EMT in the adjuvant arm compared to the non-adjuvant arm. We restricted our analysis to studies conducted using an RCT design. Additionally, studies were excluded from this analysis if they did not include a control arm (no adjuvants) as a comparator group.

## Results

### Literature search and screening

**Figure 1.**
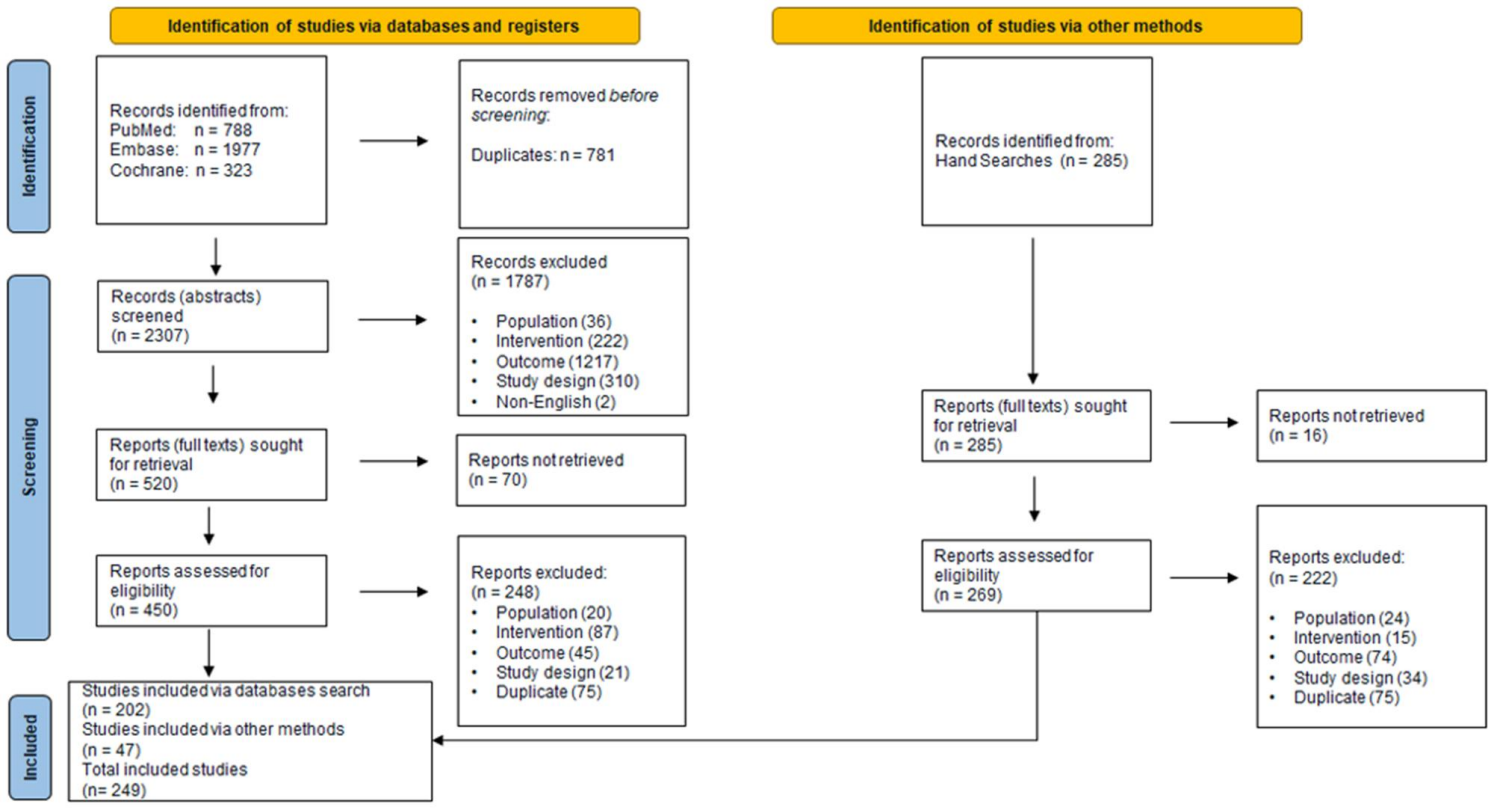
The PRISMA flow diagram of studies identified in the systematic review.

A search strategy was initially developed and run in PubMed (Supplementary Table S1), Embase (Supplementary Table S2), and Cochrane databases on 16 December 2022. The same search strategy was run again on PubMed, Embase, and Cochrane on 8 November 2024. After both searches were conducted, 788 articles were identified on PubMed, 1977 were identified on Embase, and 323 were identified on Cochrane. Before screening, 781 articles were removed due to duplication. In total, 2307 records received abstract screening, which excluded 1787 articles. Full-text screening took place with 450 articles, as 70 reports were not retrieved. There were 248 excluded articles from the full-text screening, resulting in 202 studies included. Hand searches also took place, with 285 articles identified and 269 reports retrieved. Full-text screening was performed on these publications, and 222 were excluded. The hand search resulted in 47 additional publications for an overall total of 249 included studies. A flow diagram of the process is shown in Fig. 1.

### Quality of evidence

Through the RoB assessment, study evidence quality ranged from poor to good. Most studies were of fair quality, with the second most considered to be of good quality. The smallest proportion of studies was evaluated to be of poor quality. The quality of evidence used for each meta-analysis is reported in the associated results section herein.

### Background prevalence of IUAs

Observational study designs were included for this purpose, as no controlled trials were identified that quantified the prevalence of adhesions among a population before any therapeutic intervention. The review did not identify adequate studies evaluating background prevalence in individuals without previous uterine surgery. However, several studies were identified that reported data on the prevalence of IUAs amongst various at-risk groups (such as those with abnormal uterine bleeding, infertility, and recurrent pregnancy loss) who had undergone antecedent uterine curettage. Many of these studies were performed in developing countries with a relatively high prevalence of tuberculosis (Taylor *et al.*, 1981; Valli *et al.*, 2001; Aboulghar *et al.*, 2011; Seckin *et al.*, 2012; El Huseiny and Soliman, 2013; Elsokkary *et al.*, 2018; Ajjammanavar *et al*., 2020; Ray-Offor and Nyengidiki, 2021). However, we did identify one study conducted in Italy that examined background prevalence among a population without a history of surgical intervention. This study identified 21 patients with IUAs among a population of 922, yielding a prevalence of 2% (Valli *et al.*, 2001).

### Incidence of IUAs among patients following potentially adhesiogenic procedures

#### Incidence of IUAs following hysteroscopic metroplasty for septa

**Figure 2.**
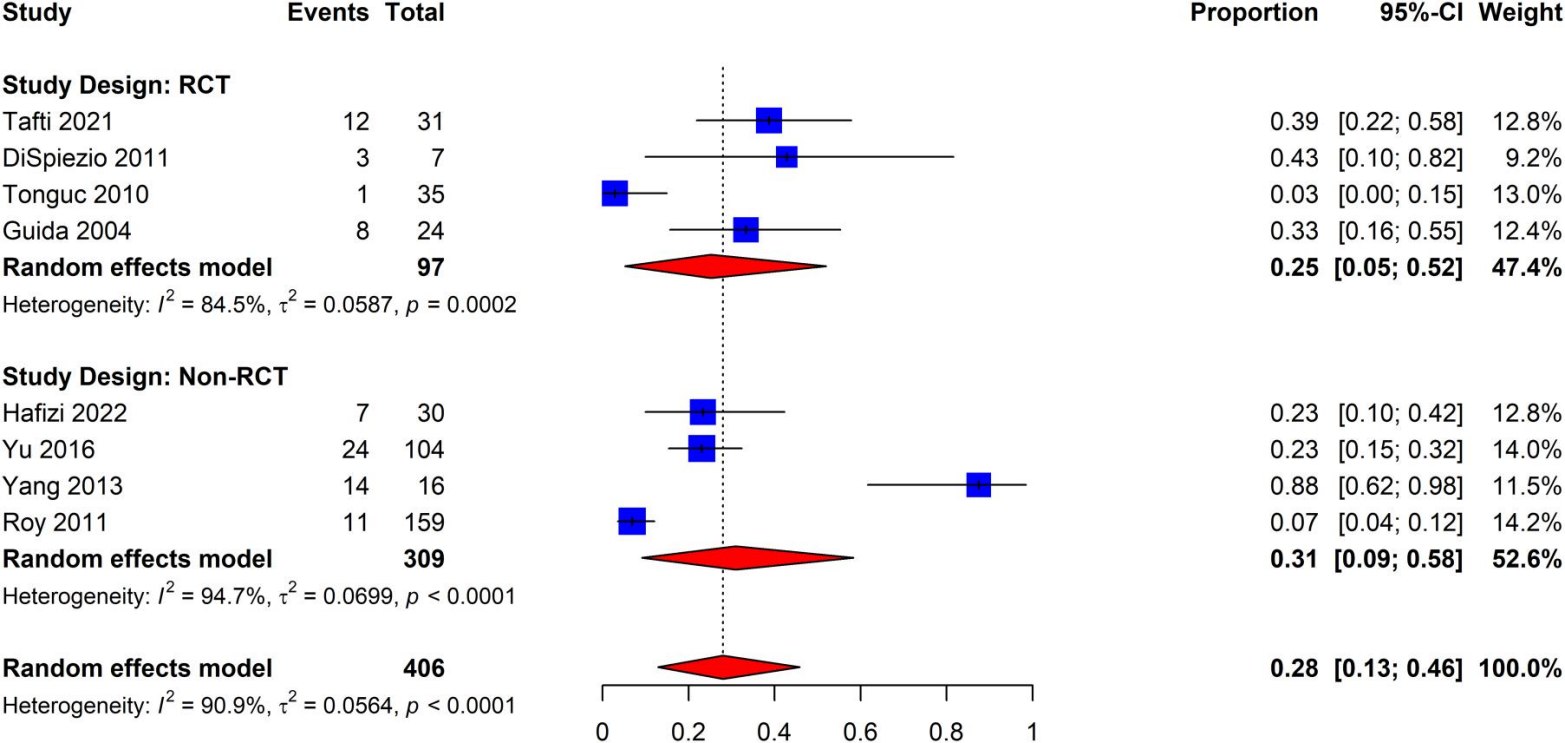
**Pooled results of meta-analysis for the incidence of IUAs following hysteroscopic metroplasty for septa.** IUAs, intrauterine adhesions; RCT, randomized controlled trial; τ, tau. Events are cases where IUAs were found at second-look hysteroscopy. Proportion refers to the risk of finding IUAs. All cases could have received antibiotics and/or estrogen-based hormone therapy.

The review identified eight eligible studies evaluating the incidence of new-onset IUAs in patients undergoing hysteroscopic metroplasty to correct a septum (four each using RCT and non-RCT designs) (Guida *et al.*, 2004; Tonguc *et al.*, 2010; Di Spiezio Sardo *et al.*, 2011; Roy *et al.*, 2011; Yang *et al.*, 2013; Yu *et al.*, 2016; Tafti *et al.*, 2021; Hafizi *et al*., 2022). Two studies were excluded from the meta-analysis as one did not investigate the incidence of IUA after metroplasty, and the other did not have a non-adjuvant arm (Supplementary Table S3). We combined the non-adjuvant arms (inclusive of antibiotics and/or estrogen-based hormone therapy use) to determine the pooled incidence. As presented in Fig. 2, the pooled incidence of new-onset IUAs post-septum correction was 28% (95% CI: 13–46%; eight studies, *I*^2^ = 91%, fair to good evidence quality). When assessed separately by RCT or non-RCT design, the incidence was 25% (95% CI: 5–52%; four studies, *I*^2^ = 85%, fair to good evidence quality) and 31% (95% CI: 9–58%; four studies, *I*^2^ = 95%, fair to good evidence quality), respectively. The measures for heterogeneity reveal substantial within-study variation in both the RCT and non-RCT subgroups. We did not identify adequate studies from the literature to evaluate adhesion severity amongst the patients undergoing metroplasty for septum correction. Additionally, we sought to study the incidence amongst the patients based on surgical technique (e.g. transection versus resection) or who had incomplete septal correction; however, sufficient studies were not available to derive the pooled estimates.

#### Incidence of IUAs following hysteroscopic myomectomy

The review found eight eligible studies evaluating the incidence of new-onset IUAs in patients undergoing hysteroscopic myomectomy (three and five using an RCT and non-RCT design, respectively) (Guida *et al.*, 2004; Yang *et al.*, 2008; Touboul *et al.*, 2009; Di Spiezio Sardo *et al.*, 2011; Yang *et al.*, 2013; Mazzon *et al.*, 2014; Huang *et al.*, 2020a; Bortoletto *et al.*, 2022). Five other studies were excluded from the meta-analyses as the design did not include evaluation of subjects treated without adjuvants, the results contained inaccurate reporting, or the results were not stratified by approach (Supplementary Table S4).

**Figure 3.**
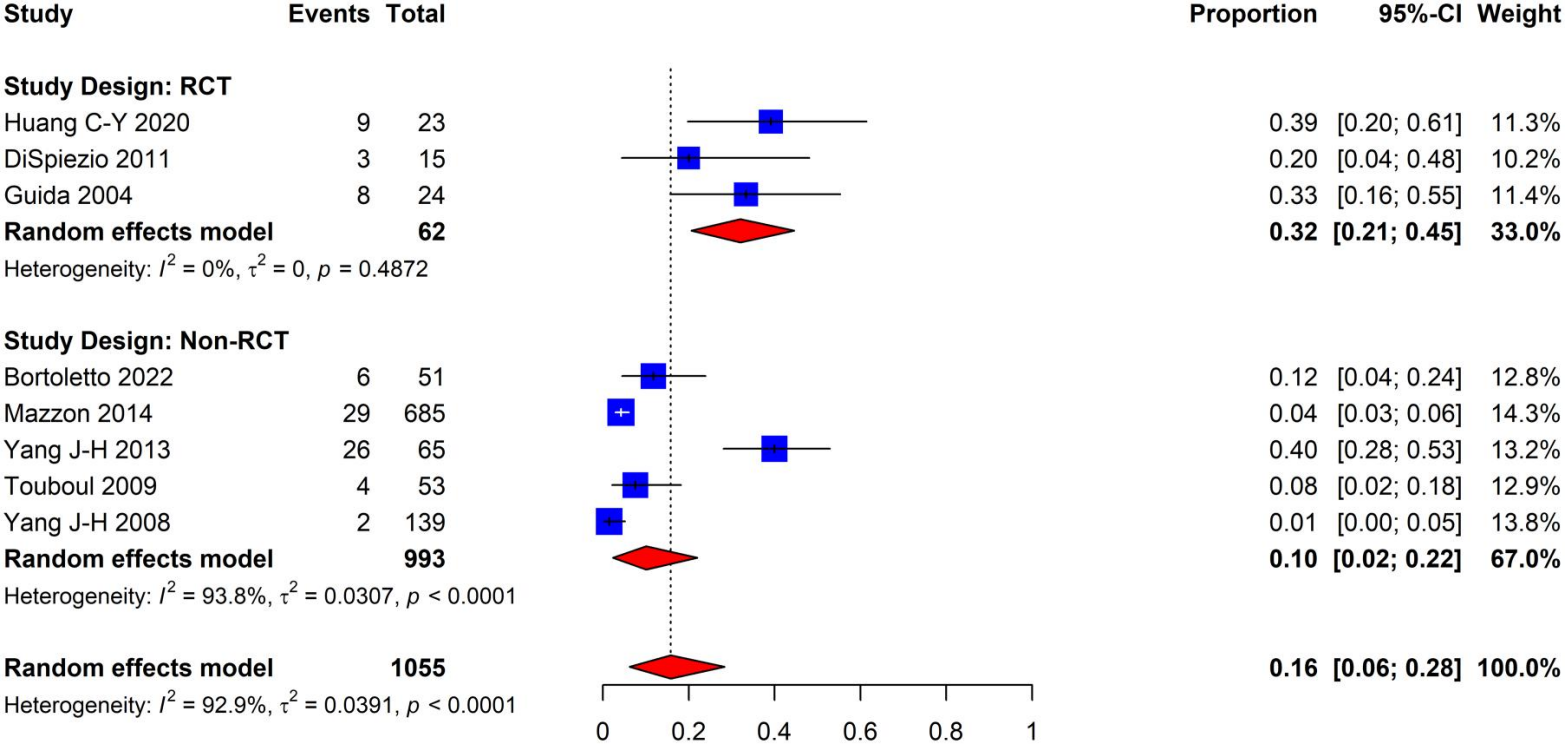
**Pooled results of the meta-analysis for the incidence of IUAs following hysteroscopic myomectomy.** IUAs, intrauterine adhesions; RCT, randomized controlled trial; τ, tau. Events include cases where IUAs were found at second-look hysteroscopy. Proportion refers to the risk of finding IUAs. All cases could have received antibiotics and/or estrogen-based hormone therapy.

The pooled incidence of new-onset IUAs post hysteroscopic myomectomy was 16% (95% CI: 6–28%; eight studies, *I*^2^ = 93%, fair to good evidence quality) (Fig. 3). Within the subgroups of studies conducted using either an RCT or non-RCT design, the incidence was 32% (95% CI: 21–45%; three studies, *I*^2^ = 0%, fair to good evidence quality) and 10% (95% CI: 2–22%; five studies, *I*^2^ = 94%, fair to good evidence quality), respectively. The non-RCT subgroup had a significantly high degree of heterogeneity.

**Figure 4.**
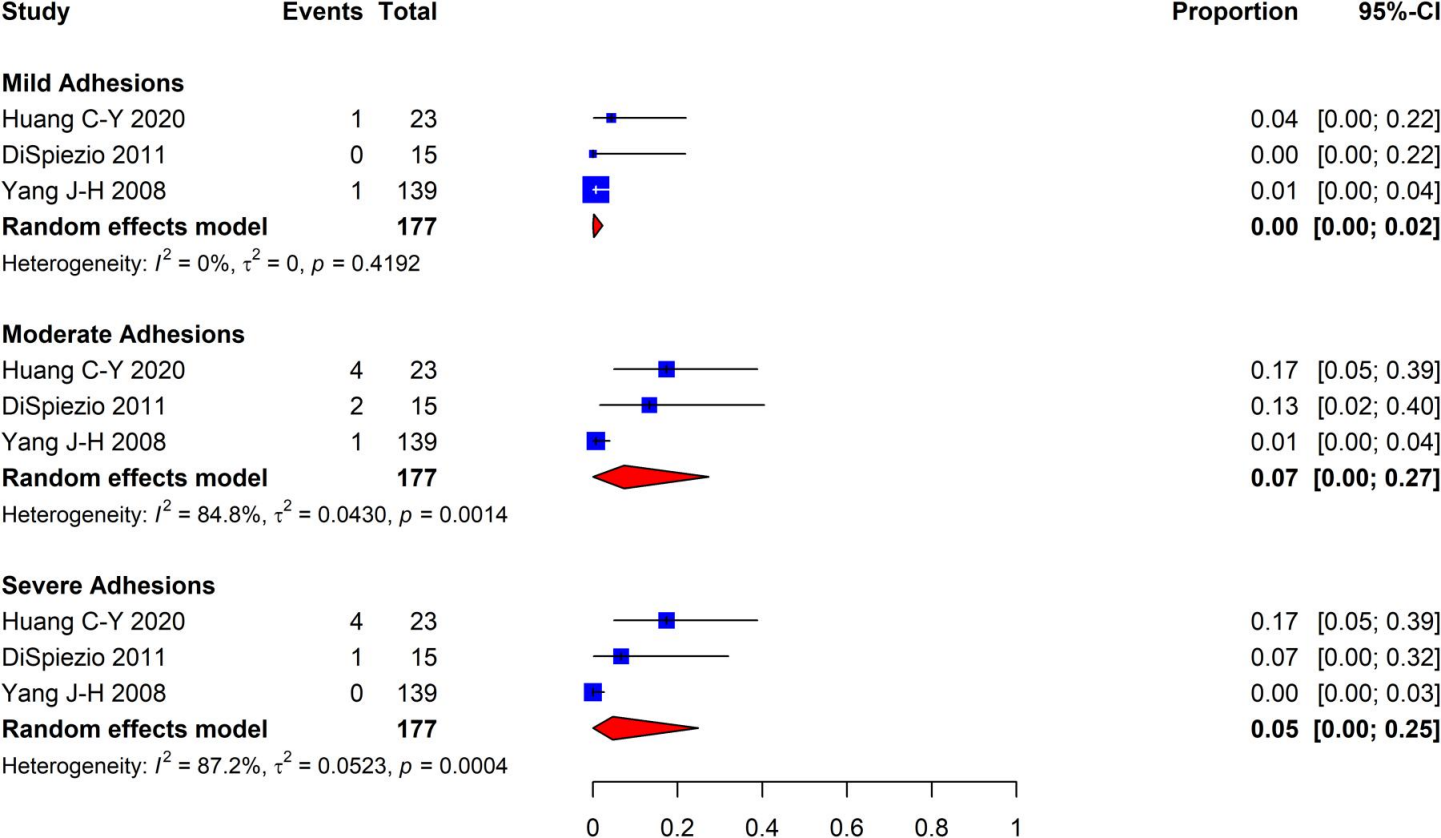
**Pooled results of the meta-analysis for the severity of IUAs following hysteroscopic myomectomy, all study types.** IUAs, intrauterine adhesions; τ, tau. Events are cases with the corresponding degree of IUA severity identified at second-look hysteroscopy. Proportion refers to the risk of finding IUAs by category of adhesion severity using the AFS classification system. All cases could have received antibiotics and/or estrogen-based hormone therapy.

As a subsequent analysis, we meta-analyzed the included studies based on adhesion severity to estimate the proportion of post-myomectomy patients with mild, moderate, and severe adhesions as per the AFS classification system. As shown in Fig. 4, the incidence of mild IUAs was determined to be 0% (95% CI: 0–2%; three studies, *I*^2^ = 0%, fair to good evidence quality) and 7% for moderate IUAs (95% CI: 0–27%; three studies, *I*^2^ = 85%, fair to good evidence quality), while the incidence of severe IUAs was 5% (95% CI: 0–25%; three studies, *I*^2^ = 87%, fair to good evidence quality). There were variable degrees of heterogeneity within groups.

#### Incidence of IUAs following abdominal myomectomy (laparoscopic or laparotomic)

**Figure 5.**
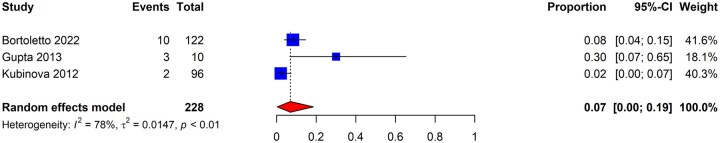
**Pooled results of the meta-analysis for the incidence of IUAs following abdominal myomectomy (laparoscopic and laparotomic), all study types.** IUA, intrauterine adhesions; τ, tau. Events are cases where IUAs were found at second-look hysteroscopy. Proportion refers to the risk of finding IUAs. Bortoletto *et al.* (2022) retrospectively evaluated a cohort of patients post laparoscopic myomectomy, whereas Gupta *et al.* (2013) retrospectively evaluated subjects post laparotomic myomectomy. Kubinova *et al.* (2012) was a prospective study that evaluated patients after either myomectomy approach. All cases could have received antibiotics and/or estrogen-based hormone therapy.

Only three eligible studies evaluated the incidence of new-onset IUAs amongst patients undergoing abdominal myomectomy (Kubinova *et al.*, 2012; Gupta *et al.*, 2013; Bortoletto *et al.*, 2022). None of the studies were randomized trials; two were retrospective, and one was prospective. Bortoletto *et al.* (2022) evaluated a cohort of patients post laparoscopic myomectomy, whereas Gupta *et al.* (2013) evaluated subjects post laparotomic myomectomy. Kubinova *et al.* (2012) was a prospective study that evaluated patients after either myomectomy approach. No studies were excluded from the meta-analysis. As presented in Fig. 5, the overall incidence of new-onset IUAs post abdominal myomectomy was 7% (95% CI: 0–19%; three studies, *I*^2^ = 78%, fair evidence quality). There was significant within-study heterogeneity amongst the included studies. The study set was inadequate for conducting any additional analyses in this subgroup, such as by myoma phenotype. Unfortunately, insufficient studies reported on the severity of adhesions following abdominal myomectomy, and thus, this outcome could not be evaluated.

#### Incidence of IUAs following removal of RPOC post delivery (postpartum)

**Figure 6.**
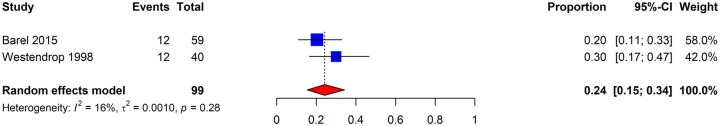
**Pooled results of the meta-analysis for the incidence of IUAs following removal of retained products of conception post-delivery (postpartum), all study types.** IUAs, intrauterine adhesions; τ, tau. Barel *et al.* (2015) retrospectively evaluated subjects who exclusively underwent a hysteroscopic approach to removal of RPOC. Westendorp *et al.* (1998) retrospectively evaluated patients who underwent dilation with sharp curettage. Events include cases where IUAs were found at second-look hysteroscopy. Proportion refers to the risk of finding IUAs. All cases could have received antibiotics and/or estrogen-based hormone therapy.

The review identified two eligible studies evaluating the incidence of new-onset IUAs in patients undergoing surgical intervention for suspected retained products following vaginal or cesarean delivery (Westendorp *et al.*, 1998; Barel *et al.*, 2015). One study was excluded due to the duplication of findings compared to those reported in Barel *et al.* (2015) (Supplementary Table S5). Figure 6 shows that the overall incidence of new-onset IUA post RPOC was 24% (95% CI: 15–34%; two studies, *I*^2^ = 16%, fair evidence quality). Both included studies were conducted using a retrospective study design. Barel *et al.* (2015) evaluated a cohort of patients who exclusively underwent a hysteroscopic approach to removal of RPOC. In contrast, Westendorp *et al.* (1998) included patients who exclusively underwent dilation and sharp curettage. The included studies had minimal within-study heterogeneity. The study set was inadequate for conducting any additional analyses in this subgroup, such as analysis by surgical technique.

#### Incidence of IUAs following removal of POC in the first trimester

**Figure 7.**
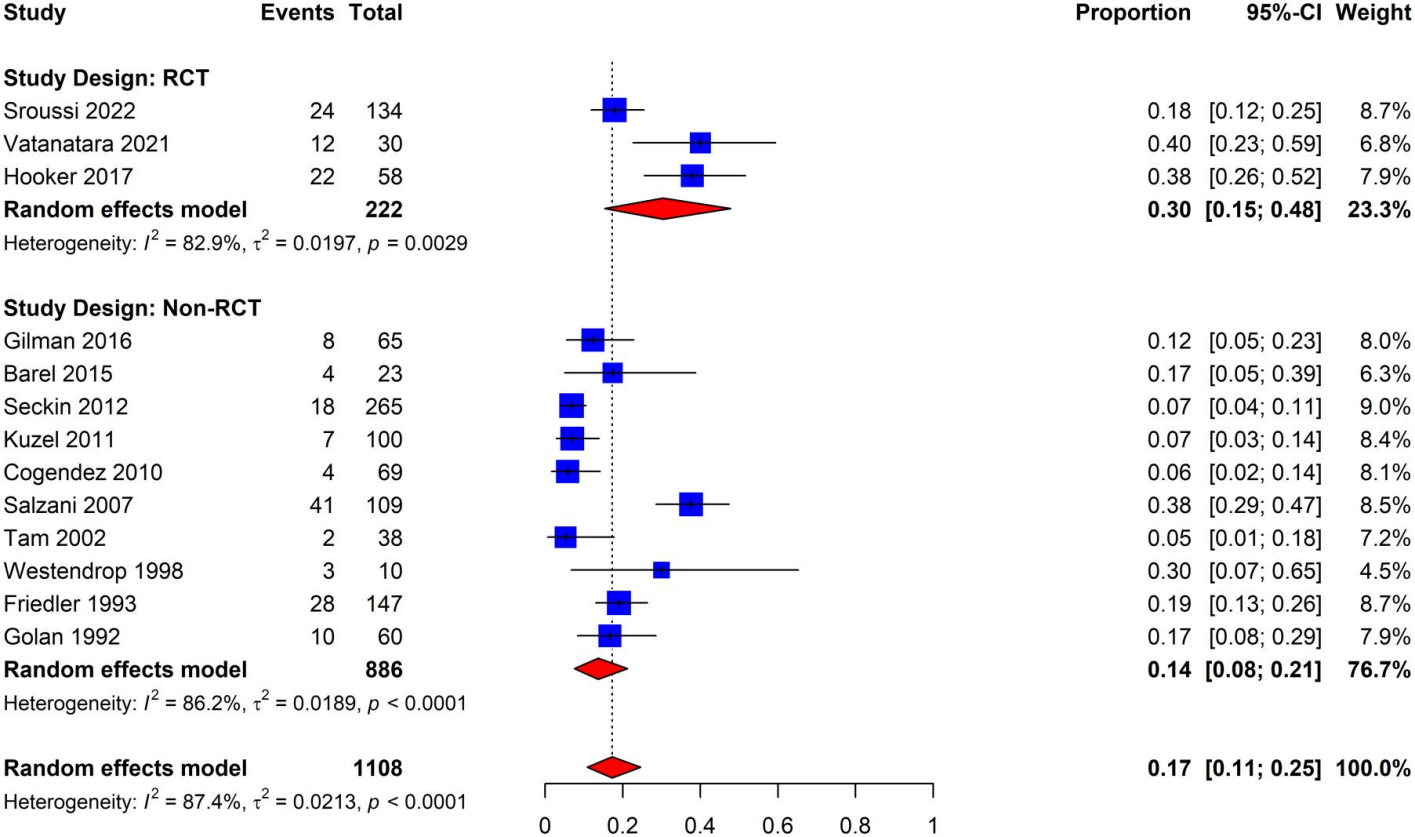
**Pooled results of the meta-analysis for the incidence of IUAs following removal of products of conception in the first trimester.** IUAs, intrauterine adhesions; RCT, randomized controlled trial; τ, tau. Events include cases where IUAs were found at second-look hysteroscopy. Proportion refers to the risk of finding IUAs. All patients experienced spontaneous pregnancy loss. All cases could have received antibiotics and/or estrogen-based hormone therapy.

The review identified 13 eligible studies to evaluate the incidence of IUAs in patients undergoing removal of POC following spontaneous pregnancy loss (three and 10 using RCTs and non-RCT study designs, respectively) (Golan *et al.*, 1992; Friedler *et al.*, 1993; Westendorp *et al.*, 1998; Tam *et al.*, 2002; Salzani *et al.*, 2007; Cogendez *et al.*, 2011; Kuzel *et al.*, 2011; Seckin *et al.*, 2012; Barel *et al.*, 2015; Gilman *et al.*, 2016; Hooker *et al.*, 2017; Vatanatara *et al.*, 2021; Sroussi *et al.*, 2022). No studies that reported the outcome of interest in the identified population were excluded from the meta-analysis. Studies that reported on patients who underwent one or more surgical procedures to remove POC were included. We combined the non-adjuvant arms (inclusive of antibiotics and/or estrogen-based hormone therapy use) to determine the pooled incidence and then separately analyzed studies based on study design (RCTs versus non-RCTs). As presented in Fig. 7, the pooled incidence of IUAs post first trimester evacuation of POC was 17% (95% CI: 11–25%; 13 studies, *I^2^* = 87%, poor to good evidence quality). Within the subgroups of studies conducted using the RCT and non-RCT designs, the incidence was determined to be 30% (95% CI: 15–48%; 3 studies, *I^2^* = 83%, poor to good evidence quality) and 14% (95% CI: 8–21%; 10 studies, *I^2^* = 86%, poor to fair evidence quality), respectively. The RCTs had minimal within-study heterogeneity, while we observed greater heterogeneity among non-RCT studies. We did not identify any peer-reviewed studies evaluating the incidence of IUAs following elective termination of a viable first trimester pregnancy.

**Figure 8.**
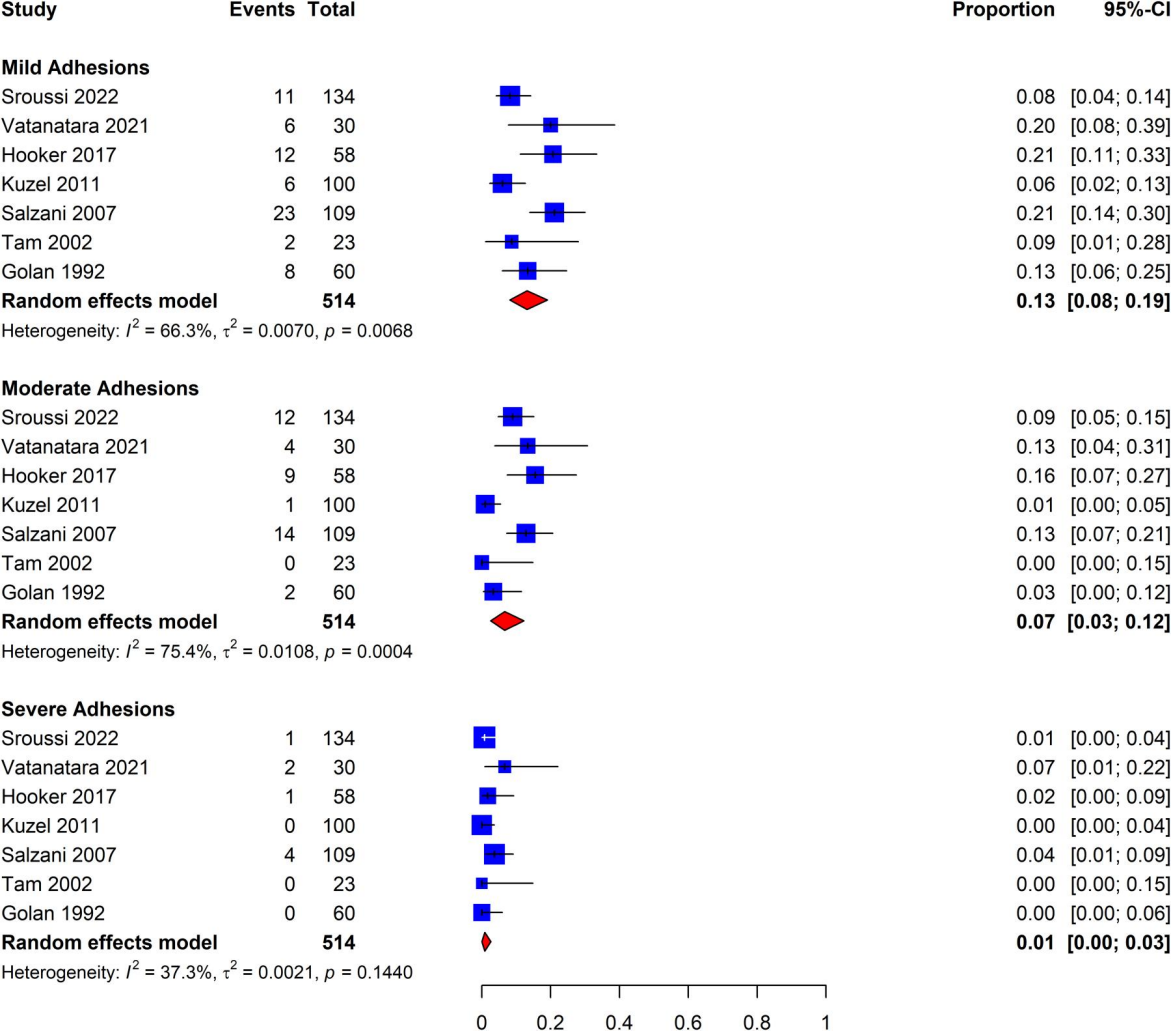
**Pooled results of the meta-analysis for the severity of IUAs following removal of products of conception in the first trimester, all study types.** IUAs, intrauterine adhesions; τ, tau. Events are cases with the corresponding degree of IUA severity identified at second-look hysteroscopy. Proportion refers to the risk of finding IUAs by category of adhesion severity using the AFS classification system. All cases could have received antibiotics and/or estrogen-based hormone therapy.

The study set was inadequate for conducting additional analyses in this subgroup; we were unable to distinguish outcomes between patients with scheduled and unscheduled (‘urgent’ or ‘emergent’) procedures. The analysis for severity of IUAs amongst patients who underwent removal of POC in the first trimester following spontaneous pregnancy loss revealed that the proportion of patients diagnosed with mild, moderate, and severe IUAs as per the AFS classification system was 13% (95% CI: 8–19%; seven studies, *I*^2^ = 66%, poor to good evidence quality), 7% (95% CI: 3–12%; seven studies, *I*^2^ = 75%, poor to good evidence quality), and 1% (95% CI: 0–3%; seven studies, *I*^2^ = 37%, poor to good evidence quality), respectively (Fig. 8).

### Recurrence of IUAs following hysteroscopic adhesiolysis

**Figure 9.**
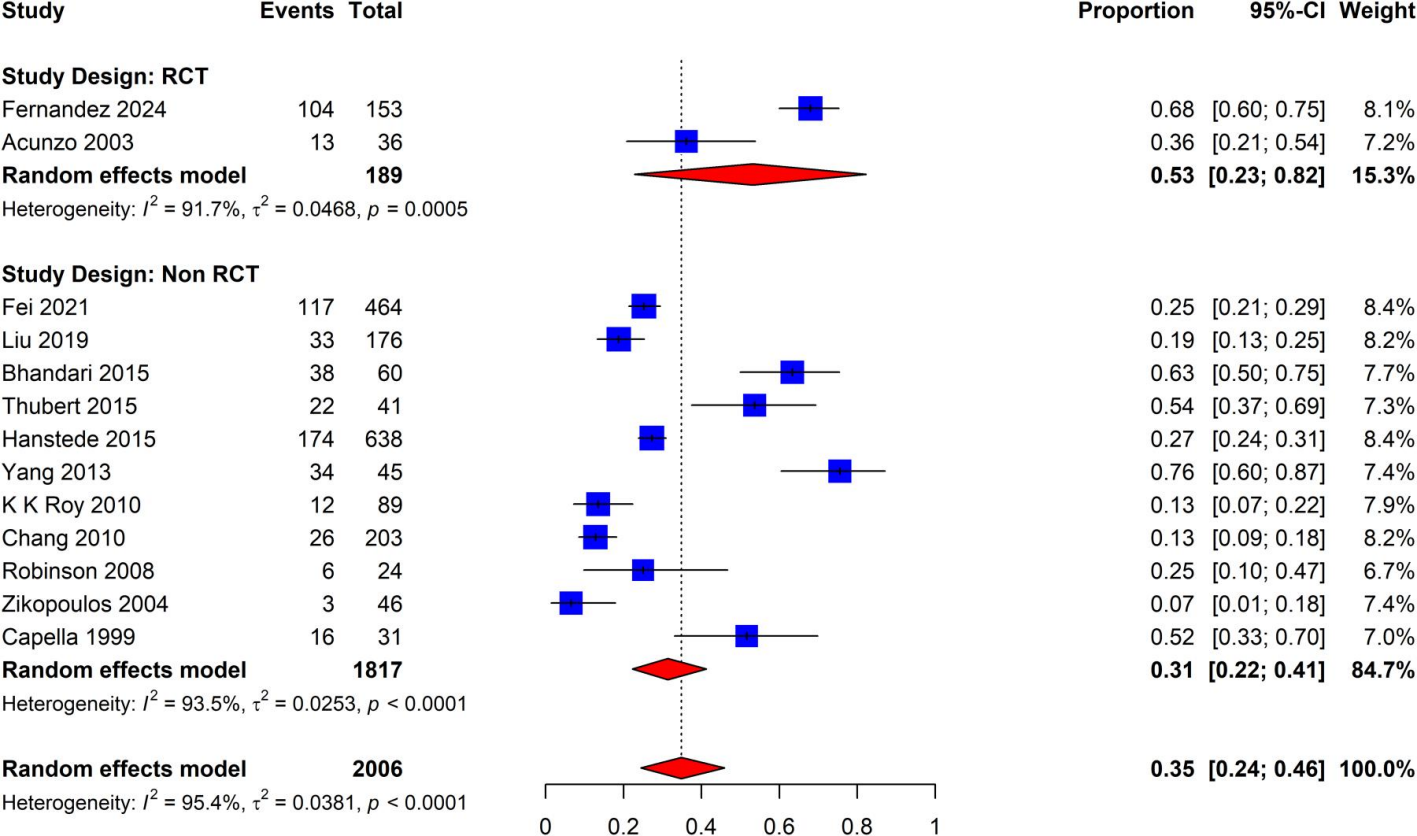
**Pooled results of the meta-analysis for the recurrence of IUAs following hysteroscopic adhesiolysis.** IUAs, intrauterine adhesions; RCT, randomized controlled trial; τ, tau. Events are cases where recurrent IUAs were identified at second-look hysteroscopy. Proportion refers to the risk of finding IUAs. All cases could have received antibiotics and/or estrogen-based hormone therapy.

The review identified 50 eligible studies that evaluated the recurrence of IUA following hysteroscopic adhesiolysis. Of those, only 13 included a cohort without adjuvant therapies and could be used to determine the IUA recurrence rate (two and 11 using RCT and non-RCT study designs, respectively) (Capella-Allouc *et al.*, 1999; Acunzo *et al.*, 2003; Zikopoulos *et al.*, 2004; Robinson *et al.*, 2008; Chang *et al.* 2010; Roy *et al.*, 2010; Yang *et al.*, 2013; Bhandari *et al.*, 2015; Hanstede *et al.*, 2015; Thubert *et al.*, 2015; Liu *et al.*, 2019a; Fei *et al.*, 2021; Fernandez *et al.*, 2024). There were four studies excluded from the meta-analysis that did not utilize hysteroscopy for evaluation or had inadequate methodology (Supplementary Table S6). We combined the non-adjuvant arms (inclusive of antibiotics and/or estrogen-based hormone therapy use) to determine the pooled recurrence rate and then separately analyzed studies based on study design (RCT versus non-RCTs). As presented in Fig. 9, the pooled recurrence rate in patients following adhesiolysis without adjuvant therapies was 35% (95% CI: 24–46%; 13 studies, *I*^2^ = 95%, poor to good evidence quality). Within the subgroups of studies conducted using either RCT or non-RCT design, the incidence was 53% (95% CI: 23–82%; two studies, *I*^2^ = 92%, good evidence quality) and 31% (95% CI: 22–41%; 11 studies, *I*^2^ = 94%, poor to good evidence quality), respectively.

**Figure 10.**
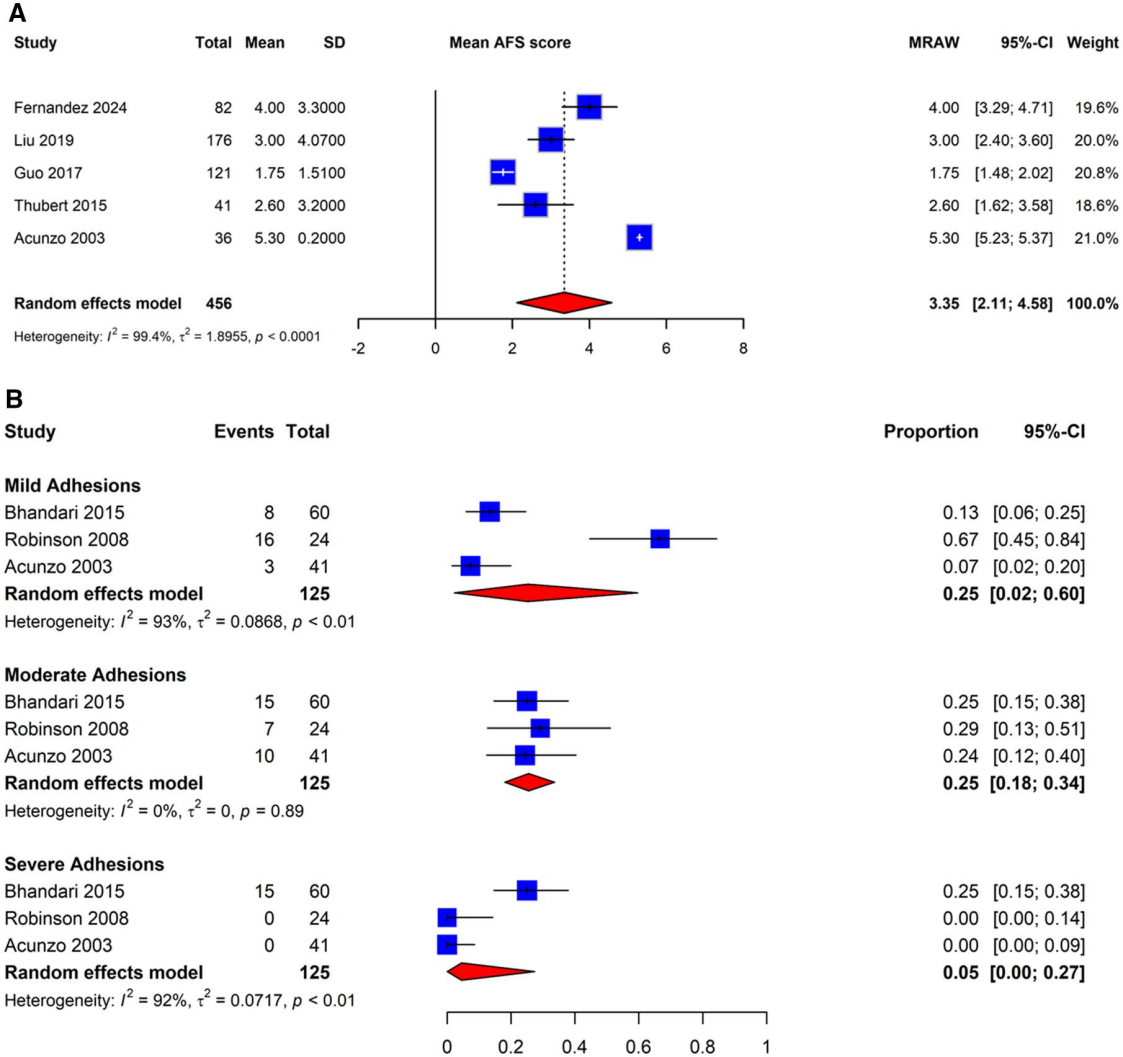
**Pooled results of the meta-analysis for the severity of IUA following hysteroscopic adhesiolysis, all study types.** (**A**) Pooled results of the meta-analysis for the mean IUA severity following hysteroscopic adhesiolysis utilizing the AFS classification system, all study types. IUAs, intrauterine adhesions; AFS, American Fertility Society; SD, standard deviation; MRAW, raw mean. (**B**) Pooled results of the meta-analysis for categorical IUA severity following hysteroscopic adhesiolysis, all study types. τ, tau. Each study utilized a different classification system: Acunzo *et al.* (2003) utilized AFS, Robinson *et al.* (2008) utilized March, and Bhandari *et al.* (2015) utilized ESGE. To allow for meta-analyses of studies using different classification systems, the severities were re-categorized as mild, moderate, or severe following the protocol described by Hooker *et al.* (2014). Events are cases with the corresponding degree of IUA severity identified at second-look hysteroscopy. Proportion refers to the risk of finding IUAs by category of adhesion severity. All cases could have received antibiotics and/or estrogen-based hormone therapy.

We meta-analyzed the included studies that reported adhesion severity at the time of second-look hysteroscopy. Of the 13 studies with non-adjuvant arms, only five were adequate for meta-analysis (Acunzo *et al.*, 2003; Thubert *et al.*, 2015; Guo *et al.*, 2017; Liu *et al.*, 2019a; Fernandez *et al.*, 2024); all utilized the AFS classification system to provide numerical severity data to estimate the proportion of post-adhesiolysis patients with mild (Stage 1: scoring from 1 to 4), moderate (Stage 2: scoring from 5 to 8), and severe adhesions (Stage 3: scoring ≥9). As shown in Fig. 10A, the mean AFS score of recurrent IUAs post hysteroscopic adhesiolysis was 3.35 (95% CI: 2.11–4.58; five studies, *I*^2^ = 99%, poor to good evidence quality), corresponding to AFS Stage 1 (mild) adhesions.

Additionally, only three studies with non-adjuvant arms reported on categorical IUA severity at the time of second-look hysteroscopy were adequate for meta-analysis (Acunzo *et al.*, 2003; Robinson *et al.*, 2008; Bhandari *et al.*, 2015); each study utilized a different classification system (AFS, March, and ESGE, respectively). To allow for meta-analyses of the studies, the severities were reconciled and re-categorized as mild, moderate, or severe using the protocol described by Hooker *et al.* (2014). As shown in Fig. 10B, the proportion of patients with mild adhesions was determined to be 25% (95% CI: 2–60%; three studies, *I*^2^ = 93%, fair to good evidence quality); for moderate adhesions, it was 25% (95% CI: 18–34%; three studies, *I*^2^ = 0%, fair to good evidence quality), and for severe adhesions, it was 5% (95% CI: 0–27%; three studies, *I*^2^ = 92%, fair to good evidence quality). The study set was inadequate for conducting additional analyses in this subgroup. Surgical techniques were often insufficiently described in the literature; thus, we did not find adequate studies to estimate the pooled recurrence of IUAs based on surgical technique, and no associations can be made between the severity of IUAs and the surgical technique used.

### Comparative effectiveness of adjuvant therapies for IUA prevention

In addition to the proportional analysis of the incidence and recurrence of IUAs, we also investigated the comparative effectiveness of adjuvant therapies in IUA prevention among the primary and secondary prevention subgroups. For these analyses, we only included RCT study designs with at least two arms, comparing an adjuvant to either no adjuvant, another adjuvant, or a combination of adjuvants.

#### Primary prevention of IUAs

The review identified a range of randomized trials that have evaluated the efficacy of adjuvant therapies for primary prevention of IUAs following potentially adhesiogenic procedures. In this analysis, we identified eight randomized trials that exclusively compared adjuvants with a non-adjuvant arm (inclusive of antibiotics and/or estrogen-based hormone therapy use) (Guida *et al.*, 2004; Tonguc *et al.*, 2010; Di Spiezio Sardo *et al.*, 2011; Hooker *et al.*, 2017; Huang *et al.*, 2020a; Tafti *et al.*, 2021; Vatanatara *et al.*, 2021; Sroussi *et al.*, 2022). Three studies were excluded from the meta-analysis that did not meet this criterion (Supplementary Table S7). Summary data on included studies for meta-analysis of primary prevention can be found in Supplementary Table S8. Of the eight studies meeting inclusion criteria, three evaluated a cohort of patients post removal of POC in the first trimester following spontaneous pregnancy loss, three post hysteroscopic myomectomy, and four post hysteroscopic metroplasty; gel was utilized as the adjuvant therapy in all these studies except for one (Tonguc *et al.*, 2010).

**Figure 11.**
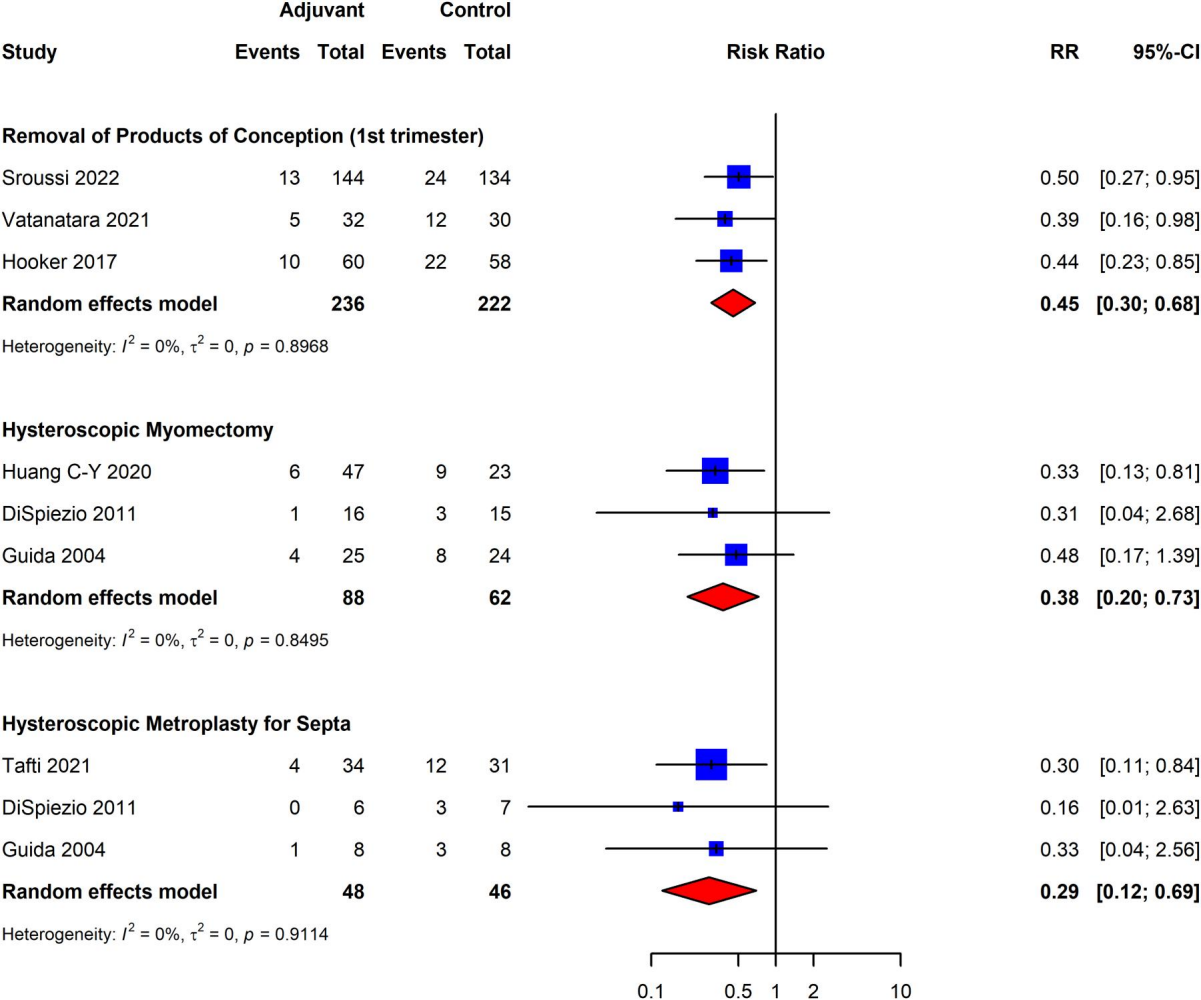
**Pooled results of the meta-analysis for the comparative effectiveness of gel adjuvant therapies versus no adjuvant for primary prevention of adhesions following potentially adhesiogenic procedures (RCT study design only).** IUAs, intrauterine adhesions; RCT, randomized controlled trial; RR, risk ratio; τ, tau. Events are cases where IUAs were identified at second look hysteroscopy. Adjuvant indicates cases receiving gel adjuvant. Control refers to cases receiving no adjuvant. While various biodegradable gel prophylactic therapies were utilized in these studies, the addition of hyaluronic acid as a component of the gel barrier was noted in all studies except for Di Spiezio Sardo *et al.* (2011). All cases could have received antibiotics and/or estrogen-based hormone therapy.

We executed a separate analysis for each potentially adhesiogenic procedure. Figure 11 presents the data selectively for the seven randomized trials that utilized gel as the adjuvant therapy, given the Tonguc *et al.* (2010) study was an outlier that evaluated the use of an IUD as adjuvant therapy and significantly altered the RR ratio within the hysteroscopic metroplasty subgroup (data for all four trials within the metroplasty subgroup are presented in Supplementary Fig. S1). As presented in Fig. 11, the RR of *de novo* IUA formation for patients undergoing primary prophylaxis with gel barrier adjuvants was 0.45 (95% CI: 0.30–0.68; three studies, *I*^2^ = 0%, poor to good evidence quality) following removal of POC in the first trimester for spontaneous pregnancy loss, 0.38 (95% CI: 0.20–0.73; three studies, *I*^2^ = 0%, fair evidence quality) following hysteroscopic myomectomy, and 0.29 (95% CI: 0.12–0.69; three studies, *I*^2^ = 0%, fair to good evidence quality) following hysteroscopic metroplasty for septa. While various biodegradable gel prophylactic therapies were utilized in these studies, the addition of hyaluronic acid as a component of the gel barrier was noted in all studies except for one (Di Spiezio Sardo *et al.*, 2011). There were inadequate studies reporting the severity of adhesions following adhesiolysis utilizing adjuvant therapy; thus, this outcome could not be evaluated. Furthermore, we did not find adequate studies to estimate the pooled recurrence of IUAs based on surgical technique.

#### Secondary prevention of IUAs

We analyzed the risk of IUA recurrence in the secondary prevention groups, comparing adjuvant therapies. We identified 18 randomized trials that evaluated the efficacy of adjuvant therapies in secondary prevention of IUAs following hysteroscopic adhesiolysis (Acunzo *et al.*, 2003; Lin *et al.*, 2015; Xiao *et al.*, 2015; Gan *et al.*, 2017; Shi *et al.*, 2019; Yang *et al.*, 2020, 2022b; Wang *et al.*, 2020b, 2022a,b; Huang *et al.*, 2020b; Pan *et al.*, 2021; Zhou *et al.*, 2021; Shen *et al.*, 2022; Guo *et al.*, 2022; Hanstede *et al.*, 2023; Fernandez *et al.*, 2024; Zhu *et al.*, 2024). There were 25 studies excluded due to either non-RCT design, lack of diagnostic hysteroscopy for patient evaluation, unspecified technique of adhesiolysis, or other reasons (Supplementary Table S9). Summary data on included studies for meta-analysis of secondary prevention can be found in Supplementary Table S10. To be eligible for inclusion, all studies must have reported on the rate of IUA recurrence. Unfortunately, there were inadequate studies reporting on the severity of adhesions following adhesiolysis utilizing adjuvant therapies, and thus, this outcome could not be evaluated. Furthermore, we did not find adequate studies to estimate the pooled recurrence of IUAs based on surgical technique.

**Figure 12.**
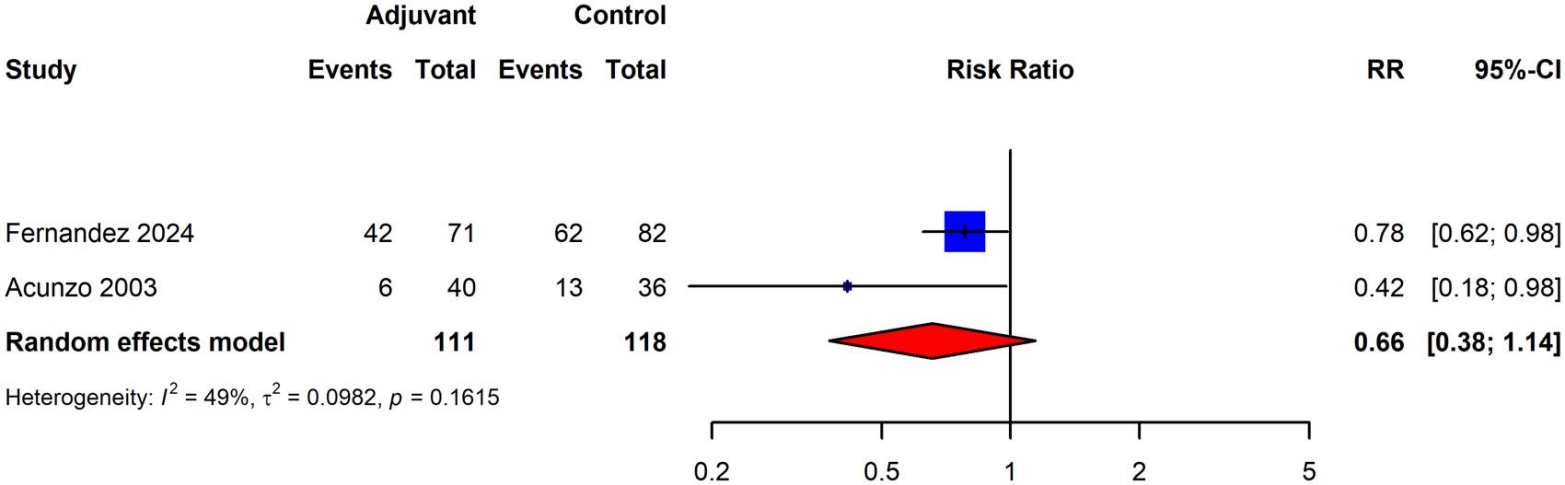
**Pooled results of the meta-analysis for the comparative effectiveness of biodegradable barriers versus no adjuvant for secondary prevention of adhesions following hysteroscopic adhesiolysis (RCT study design only).** IUAs, intrauterine adhesions; RCT, randomized controlled trial; RR, risk ratio; τ, tau. Adjuvant indicates cases receiving a biodegradable barrier. Acunzo *et al.* (2003) utilized a traditional gel adjuvant, while Fernandez *et al.* (2024) positioned a hydrophilic polymer film within the endometrial cavity that expands *in situ* to form a biodegradable barrier. Control refers to cases receiving no adjuvant. All cases could have received antibiotics and/or estrogen-based hormone therapies.

We identified only two RCTs (Acunzo *et al.*, 2003; Fernandez *et al.*, 2024) that exclusively compared a single adjuvant therapy to a non-adjuvant for secondary prevention of IUA recurrence post adhesiolysis; both utilized a biodegradable barrier for secondary prevention. Acunzo *et al.* (2003) utilized a traditional gel adjuvant, while Fernandez *et al.* (2024) positioned a hydrophilic polymer film within the endometrial cavity that expands *in situ* to form a biodegradable barrier. Based on our inclusion criteria, one otherwise eligible RCT that also utilized a biodegradable gel barrier (Mao *et al.*, 2020) was excluded due to the methodological description of the study not requiring post-adhesiolysis hysteroscopy to determine the presence and severity of IUAs. Furthermore, the study protocol also included a repeat injection of hyaluronic acid into the endometrial cavity 5–7 days following the initial adhesiolysis. The authors did not respond to two e-mail attempts to resolve this issue. The resulting meta-analysis demonstrated a risk ratio of 0.66 for recurrence of IUA post hysteroscopic adhesiolysis (two studies, *I*^2^ = 49%, good evidence quality), though the 95% CI of 0.38–1.14 did not allow for a conclusion of benefit to be made (Fig. 12). However, to assess the effect of including the Mao *et al.* (2020) study, a separate meta-analysis was conducted that demonstrated a risk ratio of 0.78 (three studies, *I*^2^ = 46%, fair to good evidence quality) but the 95% CI of 0.53–1.14 still did not allow for a conclusion of benefit to be made (Supplementary Fig. S2).

**Figure 13.**
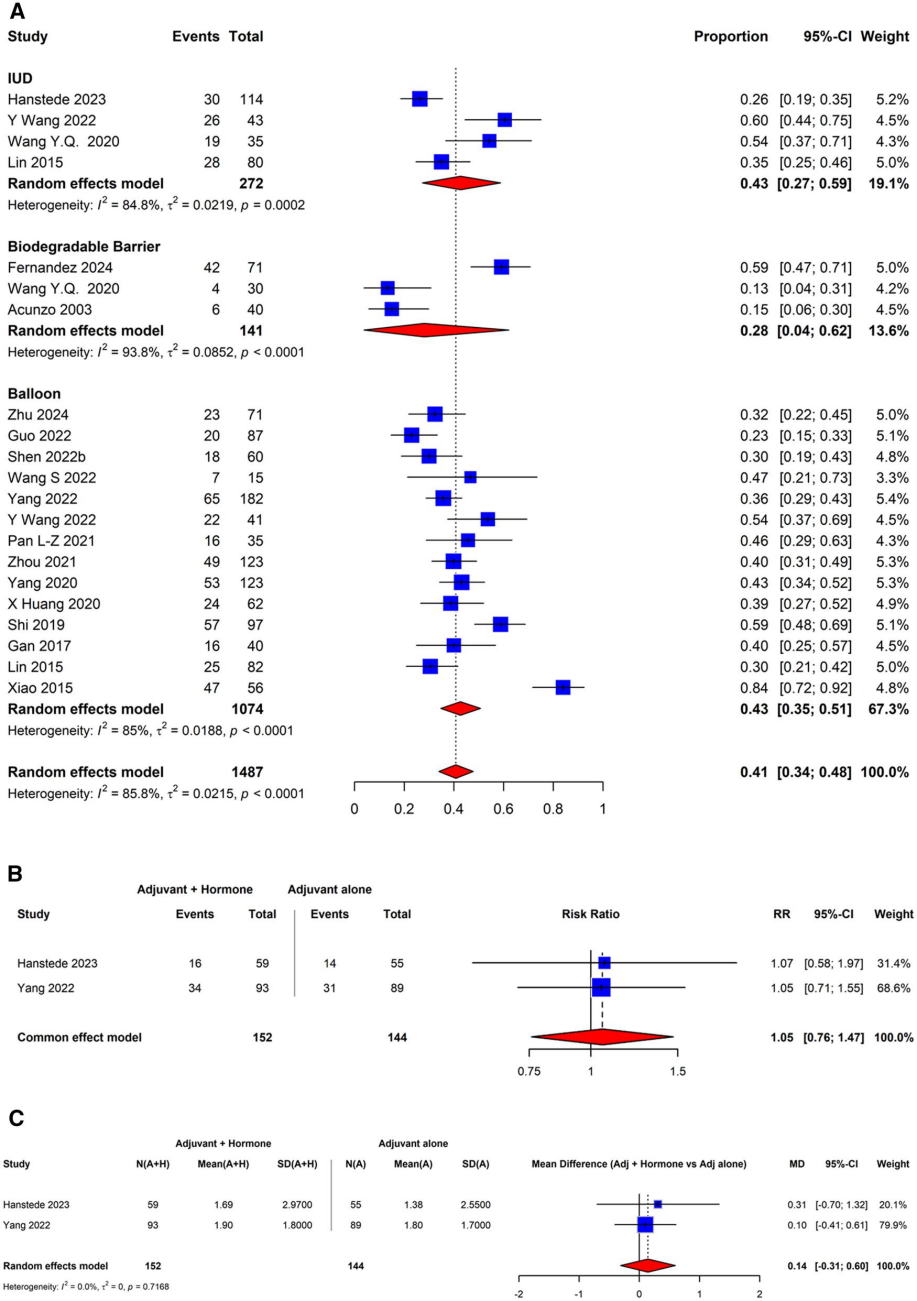
**Pooled results of the meta-analysis for the recurrence of IUAs following hysteroscopic adhesiolysis with adjuvant therapy for secondary prevention of adhesions (RCT study design only).** (**A**) Pooled recurrence of IUAs when using adjuvant therapies for secondary prevention of adhesions (RCT study design only). IUAs, intrauterine adhesions; RCT, randomized controlled trial; τ, tau. Events are cases where recurrent IUAs were identified at second-look hysteroscopy for each specified adjuvant category. Proportion refers to the risk of finding IUAs at second-look hysteroscopy by adjuvant type. Within the biodegradable barriers, Wang Y.Q. *et al.* (2020) and Acunzo *et al.* (2003) utilized a traditional gel adjuvant, while Fernandez *et al.* (2024) positioned a hydrophilic polymer film within the endometrial cavity that expands *in situ* to form a biodegradable barrier. All cases could have received antibiotics and/or estrogen-based hormone therapy. (**B**) Pooled recurrence of IUAs when using adjuvant therapies for secondary prevention with and without the concomitant use of systemic estrogens and progestins (RCT study design only). RR, risk ratio. Events on the left are cases where recurrent IUAs were identified at second-look hysteroscopy with the specified adjuvant along with systemic estrogen/progestin. Events on the right are cases where recurrent IUAs were identified at second-look hysteroscopy when the specified adjuvant was used without a systemic estrogen/progestin. The adjuvant used by Hanstede *et al.* (2023) was an inert (copper removed) ‘T-shaped’ intrauterine contraceptive device, while Yang *et al.* (2022b) used an intrauterine Foley balloon. (**C**) Pooled mean difference in the severity of recurrent IUAs when using adjuvant therapies with and without the concomitant use of systemic estrogens and progestins using the AFS classification system (RCT study design only). A, adjuvant; H, hormones—systemic estrogen/progestin; MD, mean difference; RCT, randomized controlled trial; τ, tau. The adjuvant used by Hanstede *et al.* (2023) was an inert ‘T-shaped’ intrauterine contraceptive device while Yang *et al.* (2022b) used an intrauterine Foley balloon.

A meta-analysis was performed using all 18 randomized trials that evaluated the efficacy of different adjuvant therapies in the secondary prevention of IUAs. The review identified three adjuvant therapy subgroups eligible for meta-analysis: IUD, biodegradable barriers, and intrauterine balloons. As presented in Fig. 13A, the IUA recurrence rate for patients undergoing secondary prophylaxis was the lowest among patients treated with biodegradable barriers, with an overall rate of 28% (95% CI: 4–62%; three studies, good evidence quality). The recurrence rate was 43% for both intrauterine balloon (95% CI: 35–51%; 14 studies, poor to good evidence quality) and IUD (95% CI: 27–59%; four studies, fair to good evidence quality) adjuvant therapies post adhesiolysis. We found a high degree of heterogeneity amongst the studies evaluating the intrauterine balloon adjuvants (*I*^2^ of 85%), IUDs (*I*^2^ of 85%), and biodegradable barriers (*I*^2^ of 94%).

Additional meta-analysis was performed on the two RCTs that compared the adjuvant use of barriers (IUD and intrauterine balloon, respectively) with and without concomitant use of systemic estrogens and progestins to evaluate the impact of these gonadal steroids on IUA recurrence following hysteroscopic adhesiolysis (Yang *et al.*, 2022b; Hanstede *et al.*, 2023). The pooled risk ratio of IUA recurrence after adhesiolysis was 1.05 (95% CI: 0.76–1.47; two studies, poor to good evidence quality), as presented in Fig. 13B. The same studies were subjected to meta-analysis to determine whether systemic estrogens and progestins impacted the severity of recurrent adhesions. As published data in the study by Hanstede *et al.* (2023) were not sufficient for analysis, we contacted the author, who was able to provide patient-level data on the baseline and follow-up of patients in her study. This analysis relied on the follow-up data for these two studies, as a change from baseline to follow-up was not originally reported, and only summary statistics were available from Yang *et al.* (2022b). This was deemed feasible based on guidance from Cochrane (Higgins *et al*., 2019) as well as statistical tests that found no difference in baseline scores. The results of this meta-analysis are displayed in Fig. 13C, with a mean difference of 0.14 (95% CI: −0.31 to 0.60; two studies, *I*^2^ = 0%, poor to good evidence quality). In summary, these results suggest that the use of post-adhesiolysis systemic estrogen and progestin regimens does not significantly affect the severity of recurrent adhesions.

We next sought to compare the effectiveness of different adjuvants for secondary prevention of IUA recurrence in a head-to-head fashion, grouping them according to the following categories: gel, IUD, intrauterine balloon, biologic agents (e.g. plasma-rich protein), amnion graft, and various combinations of adjuvant therapies. We identified a total of seven eligible studies; three evaluated a combination of gel with intrauterine balloon compared to intrauterine balloon alone (Xiao *et al.*, 2015; Zhou *et al.*, 2021; Guo *et al.*, 2022), two evaluated a combination of amnion graft with intrauterine balloon compared to intrauterine balloon alone (Gan *et al.*, 2017; Wang *et al.*, 2022a), and two evaluated intrauterine balloon compared to IUD as adjuvant therapies (Lin *et al.*, 2015; Wang *et al.*, 2022b). Unfortunately, we did not identify sufficient studies that exclusively compared other subgroups directly. Most head-to-head comparisons were impossible due to a lack of eligible studies making equivalent comparisons, leading to the exclusion of nine studies (Supplementary Table S11).

**Figure 14.**
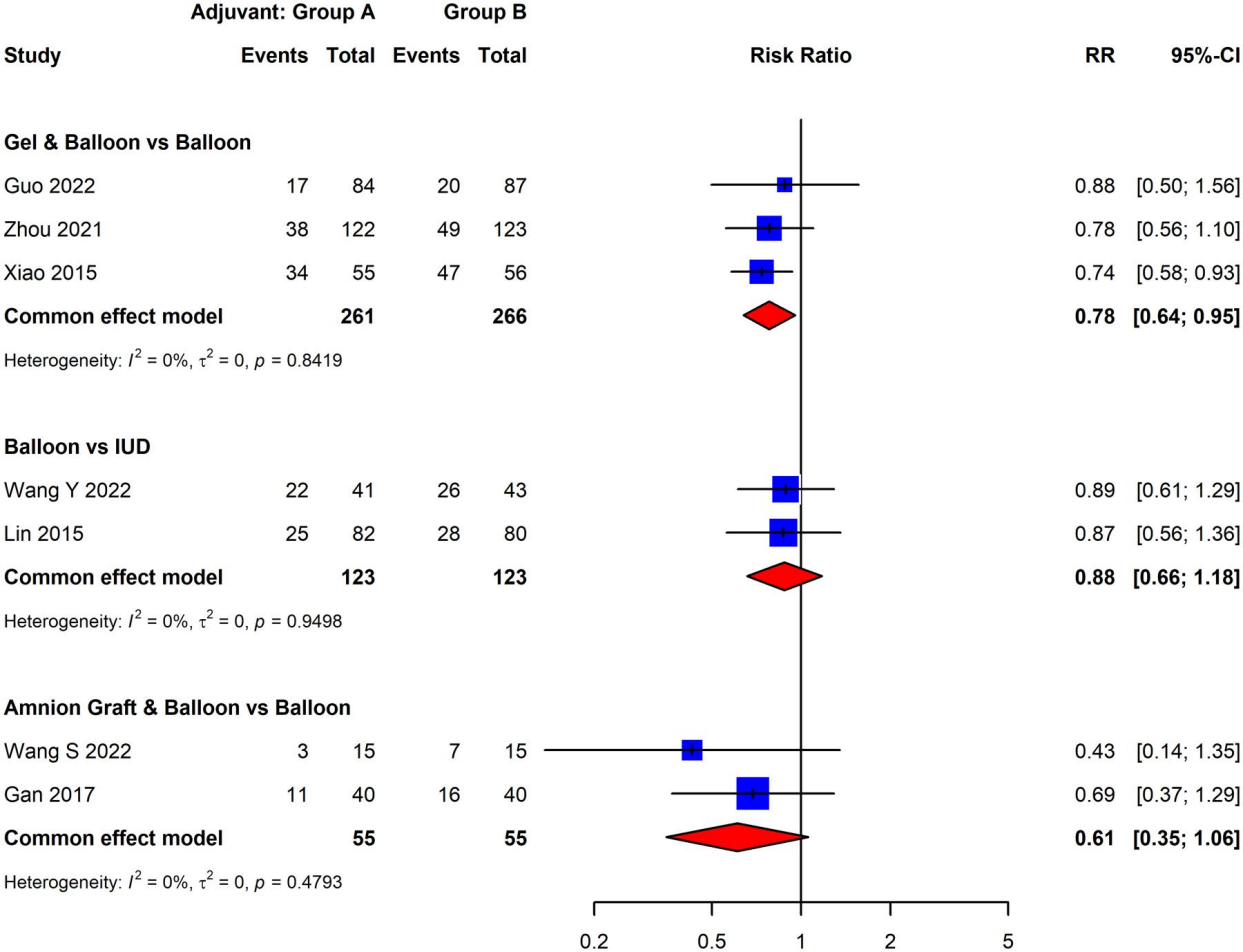
**Pooled results of the meta-analysis for the comparative effectiveness of different adjuvant therapies for secondary prevention of adhesions following hysteroscopic adhesiolysis (RCT study design only).** IUAs, intrauterine adhesions; RCT, randomized controlled trial; RR, risk ratio; τ, tau. Group A represents the first listed adjuvant while Group B represents the second listed adjuvant, e.g. in ‘Gel & Balloon vs Balloon’, Group A = Gel & Balloon and Group B = Balloon. Events are cases where recurrent IUAs were identified at second-look hysteroscopy for each specified category of adjuvant versus adjuvant. All cases could have received antibiotics and/or estrogen-based hormone therapy.

Figure 14 presents the meta-analyses for head-to-head adjuvant comparative efficacy that were feasible. The RR for the combination of gel with intrauterine balloon was found to be 0.78 (95% CI: 0.64–0.95; three studies, *I*^2^ = 0%, fair to good evidence quality), which corresponds to a RR reduction of 22% over intrauterine balloon alone, signaling marginally greater efficacy of intrauterine balloons when used in combination with gel adjuvants for secondary prevention of adhesion recurrence. In addition, we compared head-to-head intrauterine balloon adjuvants directly with IUD adjuvants and determined the RR to be 0.88 (95% CI: 0.66–1.18; two studies, *I*^2^ = 0%, fair evidence quality), which reflects that there is no statistically significant difference between these two groups of adjuvant therapies when used alone. Finally, a comparison of the combination of amnion graft with intrauterine balloon versus intrauterine balloon alone determined the RR to be 0.61 (95% CI: 0.35–1.06; two studies, *I*^2^ = 0%, fair to good evidence quality), which again reflects no statistically significant difference between these two groups of adjuvant therapies.

### Pregnancy outcomes among patients following adhesiolysis

#### Early pregnancy

We sought to identify studies evaluating the early pregnancy outcomes of women who became pregnant following adhesiolysis for IUAs. There were many studies that reported outcomes of interest, including intrauterine pregnancy, miscarriage, and ectopic pregnancy rates following hysteroscopic adhesiolysis. However, these data were of highly variable quality, and significant heterogeneity was demonstrated when attempting to group these studies for meta-analysis. There was a spectrum of gestational age groups, differing severity of adhesions at baseline, and variable combinations of non-specified, spontaneous, and assisted conception, including IVF and embryo transfer (IVF-ET)-based pregnancies among the study cohorts, which were typically not stratified by the method of conception. We also identified variations in adhesiolysis techniques and highly variable follow-up times among the studies. As such, the high heterogeneity among studies reporting early pregnancy outcomes prevented the performance of meta-analyses.

#### Obstetrical outcomes

A separate analysis was executed for the primary and secondary prevention groups to evaluate the obstetrical outcomes. The review did not identify sufficient RCTs (>1) that included outcome information on pregnancy complications following the selected uterine surgeries to allow meta-analysis. We also examined the comparative effectiveness of adjuvant therapies and non-adjuvant control groups. Still, we did not identify sufficient studies to execute comparative effectiveness meta-analyses.

**Figure 15.**
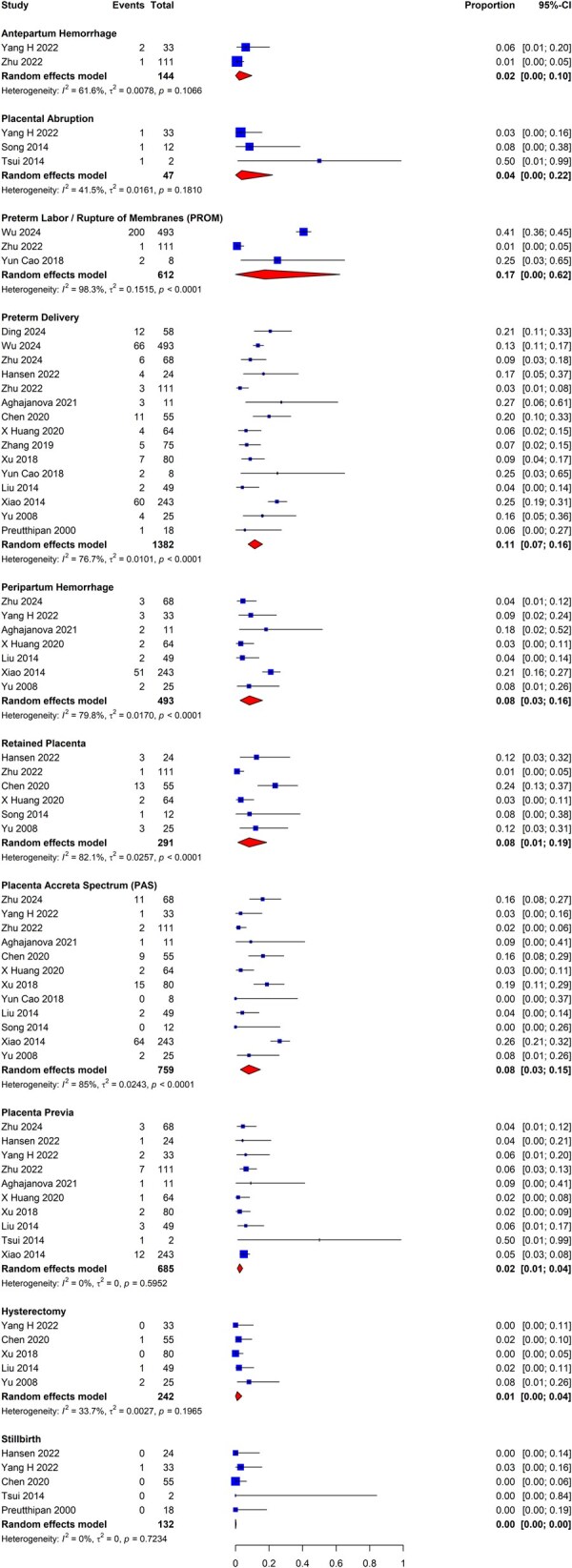
**Pooled results of the meta-analysis for the obstetrical outcomes in patients following hysteroscopic adhesiolysis with use of adjuvant therapies, all study types.** IUAs, intrauterine adhesions; PAS, placenta accreta spectrum; τ, tau. Events are cases with each specified obstetrical outcome. Proportion refers to the risk of the specified outcome. Each outcome was evaluated as a proportion of all births. Studies utilizied various adjuvant therapies, and those that included multiple treatment arms were pooled within each study. All cases could have received antibiotics and/or estrogen-based hormone therapy.

**Figure 16.**
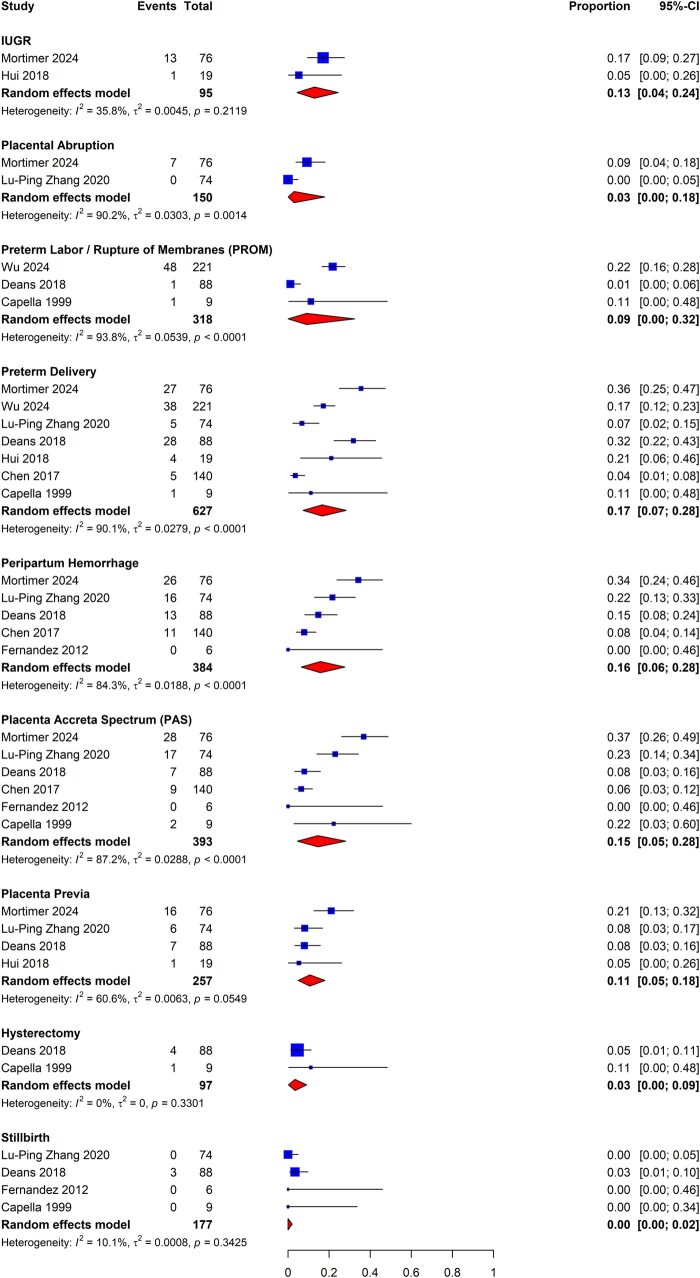
**Pooled results of the meta-analysis for the obstetrical outcomes in patients following hysteroscopic adhesiolysis with no adjuvants, all study types.** IUAs, intrauterine adhesions; PAS, placenta accreta spectrum; τ, tau. Events are cases with each specified obstetrical outcome. Proportion refers to the risk of the specified outcome. Each outcome was evaluated as a proportion of all births. Studies that included multiple treatment arms were pooled within each study. All cases could have received antibiotics and/or estrogen-based hormone therapy.

While meta-analyses of RCTs evaluating the comparative effectiveness of various techniques and interventions were not possible, we analyzed a total of 18 studies that reported obstetrical outcomes following hysteroscopic adhesiolysis with use of an adjuvant for secondary prevention (Fig. 15) (Preutthipan and Linasmita, 2000; Yu *et al.*, 2008a; Liu *et al.*, 2014; Song *et al.*, 2014; Tsui *et al.*, 2014; Xiao *et al.*, 2014; Cao *et al.*, 2018; Xu *et al.*, 2018; Zhang *et al.*, 2019; Huang *et al.*, 2020b; Aghajanova *et al.*, 2021; Hansen and Nohr, 2022; Unanyan *et al.*, 2022; Yang *et al.*, 2022a; Zhu *et al.*, 2022; Ding *et al.*, 2024; Wu *et al.*, 2024; Zhu *et al.*, 2024), and eight studies that reported on obstetrical outcomes following hysteroscopic adhesiolysis alone (Fig. 16) (Capella-Allouc *et al.*, 1999; Fernandez *et al.*, 2012; Chen *et al.*, 2017; Deans *et al.*, 2018; Hui *et al.*, 2018; Zhang *et al.*, 2020; Mortimer *et al.*, 2024; Wu *et al.*, 2024). There were 12 studies excluded from the meta-analysis due to several factors, including not separately reporting obstetrical outcomes for adjuvant and non-adjuvant groups, a mixed cohort of subjects, and absent reporting of the total number of subjects who had a live birth (Supplementary Table S12).

We found acceptable studies reporting on the following obstetrical outcomes following hysteroscopic adhesiolysis with use of an adjuvant for secondary IUA prevention: antepartum hemorrhage (n = 2), placental abruption (n = 3), preterm labor/rupture of membranes (PROM) (n = 3), preterm delivery (n = 15), peripartum hemorrhage (n = 7), retained placenta (n = 6), PAS, including placenta increta, percreta, and accreta (n = 12), placenta previa (n = 10), hysterectomy (n = 5), and stillbirth (n = 5). For patients who underwent hysteroscopic adhesiolysis without use of adjuvant therapies, we found acceptable studies reporting on IUGR (n = 2), placental abruption (n = 2), PROM (n = 3), preterm delivery (n = 7), peripartum hemorrhage (n = 5), PAS, including placenta increta, percreta, and accreta (n = 6), placenta previa (n = 4), hysterectomy (n = 2), and stillbirth (n = 4). Summary data on all included studies for meta-analysis of obstetrical outcomes following hysteroscopic adhesiolysis can be found in Supplementary Tables S13 and S14.

Each outcome above was evaluated as a proportion of all births. Studies that included multiple treatment arms were pooled within each study. As presented in Fig. 15, for the adjuvant therapy group, the rate of antepartum hemorrhage was 2% (95% CI: 0–10%; 2 studies, *I*^2^ = 62%, poor to fair evidence quality), placental abruption was 4% (95% CI: 0–22%; 3 studies, *I*^2^ = 42%, poor to fair evidence quality), PROM was 17% (95% CI: 0–62%; 3 studies, *I*^2^ = 98%, fair evidence quality), preterm delivery was 11% (95% CI: 0–16%; 15 studies, *I*^2^ = 77%, fair to good evidence quality), peripartum hemorrhage was 8% (95% CI: 3–16%; 7 studies, *I*^2^ = 80%, poor to good evidence quality), retained placenta was 8% (95% CI: 1–19%; 6 studies, *I*^2^ = 82%, fair to good evidence quality), PAS was 8% (95% CI: 3–15%; 12 studies, *I*^2^ = 85%, poor to good evidence quality), placenta previa was 2% (95% CI: 1–4%; 10 studies, *I*^2^ = 0%, poor to good evidence quality), hysterectomy was 1% (95% CI: 0–4%; 5 studies, *I*^2^ = 34%, poor to good evidence quality), and stillbirth was 0% (95% CI: 0–0%; 5 studies, *I*^2^ = 0%, poor to good evidence quality).

As reported in Fig. 16, for the non-adjuvant group, the rate of IUGR was 13% (95% CI: 4–24%; two studies, *I*^2^ = 36%, fair evidence quality), placental abruption was 3% (95% CI: 0–18%; two studies, *I*^2^ = 90%, fair evidence quality), PROM was 9% (95% CI: 0–32%; three studies, *I*^2^ = 94%, fair evidence quality), preterm delivery was 17% (95% CI: 7–28%; seven studies, *I*^2^ = 90%, fair evidence quality), peripartum hemorrhage was 16% (95% CI: 6–28%; five studies, *I*^2^ = 84%, fair evidence quality), PAS was 15% (95% CI: 5–28%; six studies, *I*^2^ = 87%, fair evidence quality), placenta previa was 11% (95% CI: 5–18%; four studies, *I*^2^ = 61%, fair evidence quality), hysterectomy was 3% (95% CI: 0–9%; two studies, *I*^2^ = 0%, fair evidence quality), and stillbirth was 0% (95% CI: 0–2%; four studies, *I*^2^ = 10%, fair evidence quality).

### Menstrual outcomes among patients following hysteroscopic adhesiolysis

The review identified four RCTs reporting the proportion of patients attaining normal menstruation pattern in women following hysteroscopic adhesiolysis with adjuvants (Amer *et al.*, 2010; Huang *et al.*, 2020b; Zhou *et al.*, 2021; Lisa *et al.*, 2025). For the proportion of patients attaining a normal menstruation pattern following hysteroscopic adhesiolysis without adjuvants, only one RCT was found (Guo *et al.*, 2017). A meta-analysis was therefore not performed for the non-adjuvant subgroup. We did not identify adequate studies reporting outcomes for ‘hypomenorrhea’ (interpreted as light menstrual bleeding) and ‘oligomenorrhea’ (interpreted as infrequent menstrual bleeding) to combine findings using meta-analysis. There were 27 studies excluded for several reasons, including non-RCT design, absence of reporting of the proportion of subjects with normal menstruation at baseline, and no description of menstrual outcomes following adhesiolysis (Supplementary Table S15).

**Figure 17.**
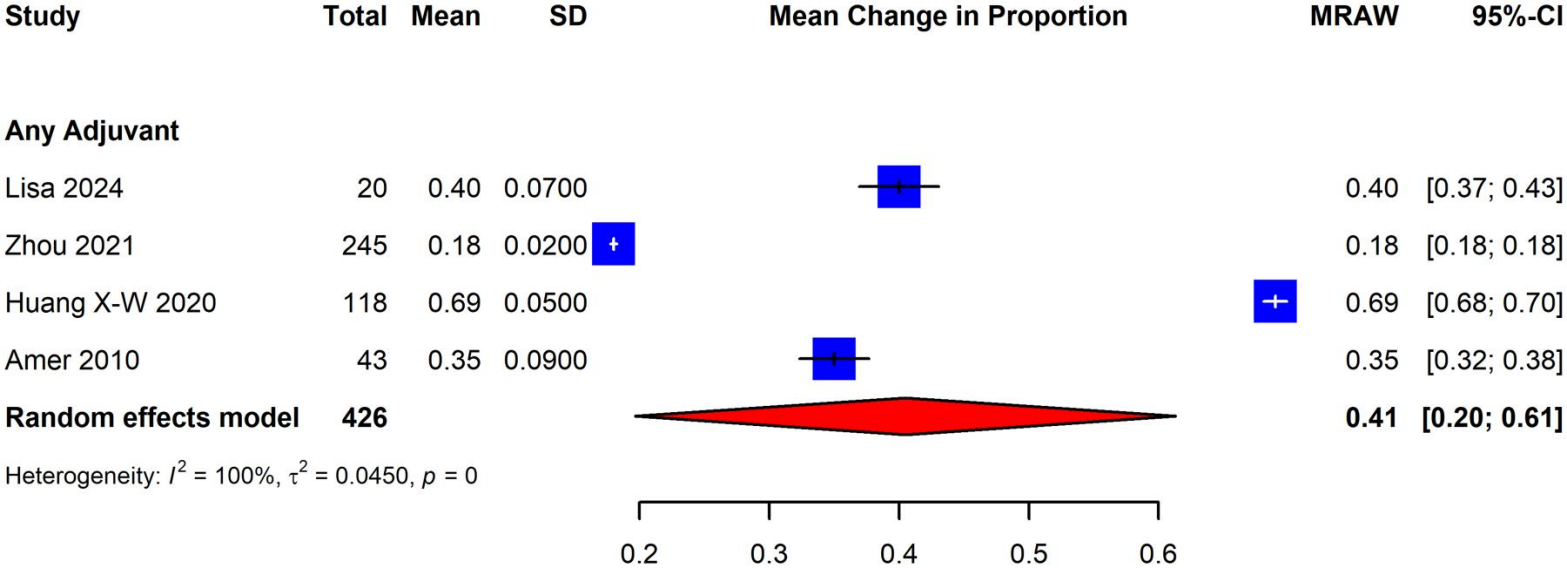
**Pooled results of the mean change in the proportion of patients attaining normal menstruation following hysteroscopic adhesiolysis (RCT study design only).** IUAs, intrauterine adhesions; MRAW, raw mean; τ, tau.

**Figure 18.**
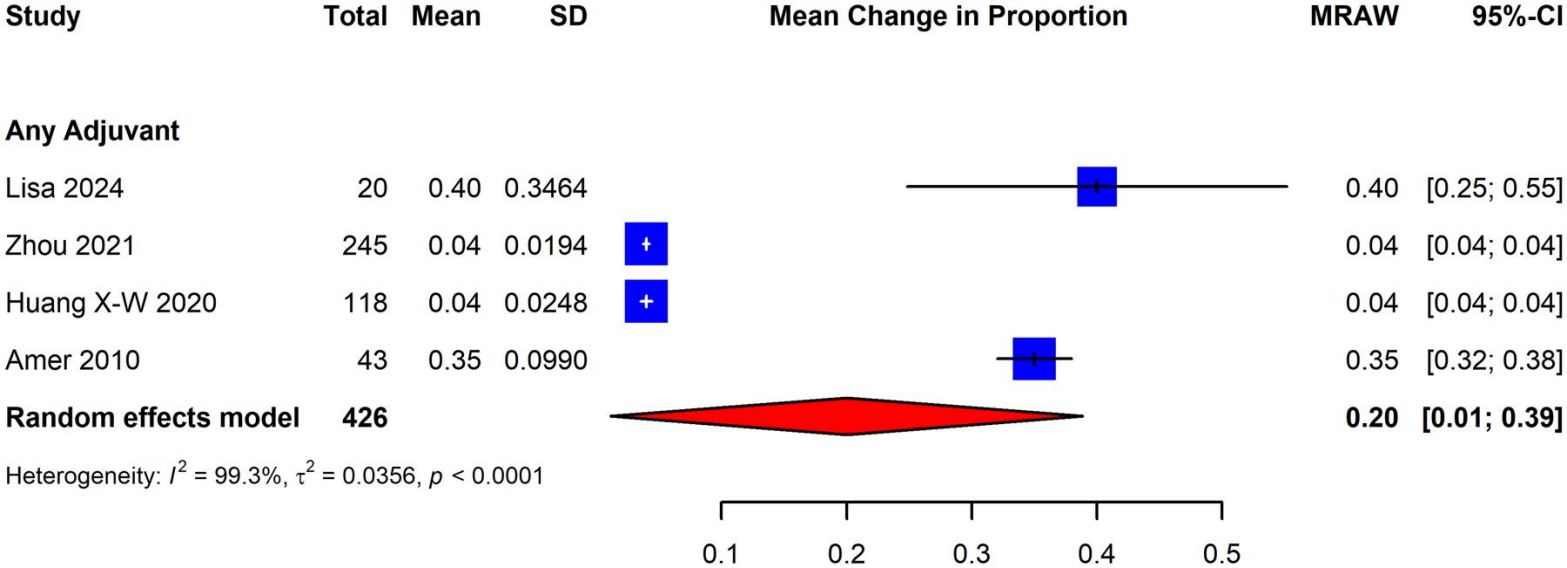
**Pooled results of the mean change in the proportion of patients with recovery of any menstruation from a baseline of amenorrhea following hysteroscopic adhesiolysis (RCT study design only).** IUAs, intrauterine adhesions; MRAW, raw mean; τ, tau.

We meta-analyzed the mean proportion of patients restoring normal menstruation at follow-up compared to the proportion of patients reporting normal menstruation at baseline. As shown in Fig. 17, the mean change in the proportion of patients with reported normal menstruation post adhesiolysis with use of adjuvant therapies was 0.41 (95% CI: 0.20–0.61; four studies, *I*^2^ = 100%, good evidence quality). Additionally, we looked at studies reporting analogous data on women with recovery of any menstruation from a baseline of amenorrhea following hysteroscopic adhesiolysis. Based on the pooled estimates from the four included studies (Amer *et al.*, 2010; Huang *et al.*, 2020b; Zhou *et al.*, 2021; Lisa *et al.*, 2025) (Fig. 18), the mean change in proportion of patients that resolved their amenorrhea post adhesiolysis with use of adjuvant therapies was found to be 0.20 (95% CI: 0.01–0.39; four studies, *I*^2^ = 99%, good evidence quality).

Overall, the positive change scores in both groups indicate that hysteroscopic adhesiolysis with the use of adjuvant therapies ameliorates adverse menstrual outcomes in women. However, we did not find sufficient studies in either group to evaluate non-adjuvant arms for comparison of effect over hysteroscopic adhesiolysis alone. Finally, there was a significant heterogeneity amongst the included studies in both these analyses, which indicates that fitting a random-effects model for our analysis was appropriate. There was a considerable difference in the effect sizes among the included studies.

### Changes in EMT among patients following adhesiolysis

We identified four studies, including three RCTs and one listed as a quasi-RCT, that evaluated the efficacy of adjuvants in improving EMT following hysteroscopic adhesiolysis (Pabuçcu *et al.*, 2019; Mao *et al.*, 2020; Wang *et al.*, 2020b; Zhu *et al.*, 2024). All studies reported the mean EMT (mm) at baseline; however, not all specified precisely when in the menstrual cycle the baseline EMT was measured or whether hormonal supplementation was in use. Post-procedure, Mao *et al.* (2020) measured the EMT on the day of embryo transfer, while the measurements of Wang *et al.* (2020b) were on Days 12–14 of the third post-procedure menstrual cycle, and Zhu *et al.* (2024) measured in the late proliferative phase. Pabuçcu *et al.* (2019) did not report on the timing of the measurements. Whereas three studies evaluated the effectiveness of gel barriers in increasing mean EMT in women with prior diagnosis of IUAs following hysteroscopic adhesiolysis, one compared Foley balloon with and without a bone marrow stem cell scaffold (Zhu *et al.*, 2024). Only Mao *et al.* (2020) compared the gel barrier to a non-adjuvant arm. The studies by Wang *et al.* (2020b) and Pabuçcu *et al.* (2019) compared patients treated with gel barriers to those treated with IUDs, as well as those treated with both gels and IUDs. Therefore, meta-analysis was not feasible due to differences in adjuvants used and the differences in the measurement timing of EMT.

## Discussion

### General considerations

Endometrial trauma from a variety of uterine surgical procedures has the potential to adversely affect menstrual function and fertility, and for those who conceive, it may be associated with a high prevalence of adverse pregnancy outcomes that can have substantial adverse effects on the woman, the fetus, and the subsequent newborn, as well as on healthcare resource utilization. While the results of this work generally support these observations, many limitations exist in these data. These include heterogeneity in study design, including variations in the nature and phenotype of the disease state, differences in the techniques used to perform procedures, variable constructs and use of post-surgical adjuvants designed to prevent or reduce the severity of IUAs, and inconsistency in the methods of assessing surgical outcomes. While our methodology required that baseline and post-surgical assessments of adhesions be performed using hysteroscopy, it was frequently difficult to determine if the surgeon performing the assessment was blinded to the intervention(s) being evaluated. It is also difficult to determine the degree of publication bias that may be present, as investigations that show no or minimal improvement in IUA metrics may not have been published.

This systematic review highlights the many nuanced but still important issues to consider when analyzing outcomes associated with different adjuvant therapies. For example, IUDs vary in shape, size, and with inert, hormonal, and/or metallic components. Intrauterine balloons also vary in size and shape, and the methodology may describe different volumes of fluid used for distension. Both IUDs and balloons can be left within the uterus for a wide range of days to weeks (a variable that can be called residence time), while balloons may be variably deflated and reinflated or removed and reintroduced. Biodegradable barriers such as gels and hydrophilic films distribute or expand to form a barrier between the surfaces of the uterine cavity, but can vary in the duration of time before they dissolve (residence time) and in their composition by including perceived inert but potentially therapeutic agents such as hyaluronic acid. Within the biological agent category, the application of plasma-rich protein, amnion graft, and instillation of stem cells can also vary in quantity, length of time, and method of application. Furthermore, any of these interventions may be used alone or in combination, a circumstance that was commonly encountered in this review. In many cases, there were insufficient data to make comparisons, and therefore, only proportional meta-analyses could be conducted to assess the incidence of adhesions and other outcomes among women with a common adjuvant therapy, including no adjuvant. This limitation should be recognized, and readers are discouraged from making comparative conclusions based on these proportional, non-comparative meta-analyses.

These issues are further complicated by the existence of a plethora of adhesion classification systems and the absence of well-defined and executed direct and surrogate methods for measuring the function of the endometrium. Moreover, the rigor in reporting fertility and pregnancy outcomes is highly variable, thereby limiting the ability to determine how, for example, various techniques for adhesiolysis, different anti-adhesion adjuvants, or agents designed to facilitate functional endometrial repair might also mitigate the incidence of subsequent infertility, recurrent pregnancy loss, and adverse peripartum outcomes.

Several systematic reviews and meta-analyses have been published in the literature over the last decade, generally examining a singular aspect of the overall clinical issue, including the use of one or a variety of adjuvants, as well as pregnancy (Hooker *et al.*, 2014; Healy *et al.*, 2016; Guo *et al.*, 2019; Albazee *et al.*, 2022; Chen *et al.*, 2022; Unanyan *et al.*, 2022; Korany *et al.*, 2022; Tang *et al.*, 2023; Luo *et al.*, 2024; Wang *et al.*, 2025) or obstetrical outcomes (Guo *et al.*, 2019; He *et al.*, 2023; Wenzhi *et al.*, 2023). The development and use of novel biotechnologies designed to initiate or facilitate endometrial regeneration has also been explored (Rodriguez-Eguren *et al.*, 2024; Chen *et al.*, 2022). For this work, we chose to review the evidence relating to these outcomes but also sought to evaluate the literature more extensively, identifying data on background prevalence, the incidence following a variety of procedures impacting the endometrial cavity, and the comparative effectiveness of different adjuvant therapies on primary and secondary prevention of IUAs, the phenotypes of pathology such as adhesion severity, septum phenotype, leiomyoma number, volume, and category, and evidence pertaining to the techniques used to perform uterine surgery for a variety of conditions. We chose not to include endometrial polypectomy in this review given the relative lack of publications and because the risks of IUAs related to the removal of polyps, in general, seemed to be extremely low. This comprehensive approach was considered essential to provide a global assessment of the available evidence and identify the evidence gaps in a way that would inform the clinical and academic community and direct future research. To describe the limitations of the available literature and our recommended approaches and strategies to address these limitations, we have developed a summary and needs assessment (Table 2) as part of this publication.

**Table 2.** Summary and needs assessment.

Issue			Findings/limitations	Recommendations
1	**Reframing and renaming:** **Do we have the right name for the condition?**		IUAs are a visible manifestation of damage to the endometrial basalis that has, to a variable degree, undergone a process of pathological repair. It is apparent that the division/removal of adhesions alone does not address the underlying endometrial damage given the high risk of recurrence of adhesions as well as associated persisting infertility. When pregnancy occurs, there remain increased risks of adverse pregnancy-related outcomes, including preterm labor and peripartum hemorrhage, often secondary to placenta accreta spectrum disorders.	Reframing this condition could provide a more conceptually accurate umbrella term. Options to consider include ‘endometrial trauma’ or ‘endometrial damage’. IUAs will continue to be of value as a surrogate outcome, but other outcomes that reflect endometrial integrity and function should be developed, validated, and used in trials.
2	**Prevalence and pathogenesis**		There is limited understanding of the worldwide background prevalence of adhesions among a general population with no reported history of uterine surgeries as well as the etiology of endometrial damage and related IUAs, including the impact of infection. While experts agree that uterine infection concomitant with endometrial trauma worsens the severity of IUA formation, the extent of this impact remains unknown. Given the global patterns of emigration and immigration, tuberculosis may be a contributor beyond the borders of countries where the disorder is known to be endemic.	Development, worldwide uptake, and access to systems designed to determine the prevalence and etiology of endometrial trauma and related IUAs are needed. In addition to surgical trauma, understanding how uterine infection impacts IUA formation, with development of diagnostic criteria for acute and chronic uterine infections that correlate with clinical outcomes, and exploration of potential therapeutic options for management, including the role of antibiotics. Understanding the relationship of image-guided procedures such as uterine artery embolization (UAE) and ablative therapies for leiomyomas (e.g. focused ultrasound, microwave, and radiofrequency electrical energy) impact IUA formation. It will also be essential to consider the context in which the endometrial trauma occurs. The hypoestrogenic postpartum patient may be especially susceptible. Heightened clinical awareness of the presence of tuberculosis in developed countries with substantial immigration from endemic areas.
3	**Incidence of IUAs following uterine surgical procedures**			
	a	Removal of POC—early pregnancy	While the incidence of IUAs following surgical evacuation of POC following spontaneous pregnancy loss in the first trimester based on RCTs was 30% (95% CI: 15–48%; three studies, *I*^2^ = 83%, poor to good evidence quality). The proportion of patients diagnosed with mild, moderate, and severe IUAs was 13% (95% CI: 8-19%; seven studies, *I*^2^ = 66%), 7% (95% CI: 3-12%; seven studies, *I*^2^ = 75%), and 1% (95% CI: 0-3%; seven studies, *I*^2^ = 37%), respectively. The differences in the risk of IUA formation following spontaneous miscarriage compared to emergent and elective evacuation of the uterus in early pregnancy remain unclear. We were unable to identify peer-reviewed studies that evaluated the incidence of IUAs in patients undergoing elective termination of a viable pregnancy.	Prevalence following evacuation of the uterus in different clinical situations is an important issue to resolve. Investigations should be designed to collect variables related to spontaneous pregnancy loss including elective versus emergent evacuation procedures. Furthermore, investigations of IUAs in patients undergoing elective termination of viable pregnancies should be undertaken. Recent advances in hysteroscopically directed removal of products of conception should continue to be investigated. Studies should be designed to measure both the incidence and the severity of IUAs, preferably with a consensus-based classification system. When measures designed to assess menstrual function are adequately defined and validated, they should be included in the outcomes.
	b	Removal of retained POC—postpartum	While data examining the risk of IUAs secondary to removal of RPOC after delivery are even more sparse than for early pregnancy, the estimate based on available RCT data is 24% (95% CI: 15–34%; two studies, *I*^2^ = 16%, fair evidence quality), a result with a high degree of variation. We could not find adequate studies examining the severity of such adhesions. There are inadequate data to inform our understanding of the severity of the trauma and related IUAs in this cohort.	All that applies to POC (including RPOC) in early pregnancy applies to postpartum management of RPOC, which is typically associated with some additional degree of acute uterine bleeding. The mechanics of extraction and the consequences of such procedures will vary depending on the timing of diagnosis relative to delivery. Study designs should consider all these aspects. While experts agree that uterine infection, such as postpartum endometritis concomitant with endometrial trauma worsens the severity of IUA formation, the extent of this impact remains unknown.
	c	Removal of a uterine septum	The most commonly treated Müllerian anomaly is the uterine septum, but there exists a spectrum of septum phenotypes ranging from those that are one cm in length to those that extend across the endometrial cavity through the cervical canal to the external os. There is controversy regarding the utility of removing the septum in improving relevant clinical outcomes. There is wide variation in the 25% (95% CI: 5–52%; four studies, *I*^2^ = 85%, fair to good evidence quality) incidence of IUAs based on RCTs following removal of a septum. There are inadequate data to inform our understanding of the severity of the trauma and related IUAs in this cohort. In addition to the differences in septum phenotype, there are substantial differences in the techniques used to remove septae, including transection versus resection and mechanical versus electrosurgical technique. We could find no studies comparing IUA outcomes based on septum phenotype, mechanical versus electrosurgical removal, or transection versus resection of the septum.	Incidence studies should be designed based on: - Septum phenotype, e.g. CONUTA classification. - Strategy: transection versus resection. - Technique: energy-based versus mechanical. Studies should report on the incidence of incomplete septum removal.
	d	Myomectomy	Data from RCTs suggest that the risk of IUAs following hysteroscopic myomectomy is 32% (95% CI: 21–45%; three studies, *I*^2^ = 0%, fair to good evidence quality). Our analysis demonstrates that the incidence of mild IUAs is 0% (95% CI: 0–2%; three studies, *I*^2^ = 0%, fair to good evidence quality), moderate IUAs is 7% (95% CI: 0–27%; three studies, *I*^2^ = 85%, fair to good evidence quality) and severe IUAs is 5% (95% CI: 0–25%; three studies, *I*^2^ = 87%, fair to good evidence quality). While it is widely perceived that removal of certain leiomyoma locations (lateral, cornual) and phenotypes (multiple and opposing) is associated with a higher incidence of IUA, we did not find adequate design or number of studies to perform meta-analyses. We were unable to identify studies comparing IUA outcomes based on myoma phenotype or by surgical techniques (mechanical versus electrosurgical removal). Our analysis of the risk of IUAs following abdominally performed (laparoscopic or laparotomic) myomectomy suggested a risk of 7% (95% CI: 0–19%; three studies, *I*^2^ = 78%, fair evidence quality) but was limited by study number and quality, as none of the studies were randomized trials.	For leiomyomas, it will be essential to evaluate the impact on IUAs of image-guided therapies such as UAE or radiofrequency ablation with various techniques, in addition to surgical correction with myomectomy. In addition to investigating the impact of surgical technique on IUA formation, techniques to hydrodissect along the pseudocapsule of the myoma should continue to be investigated. Studies should report on the incidence of incomplete myoma resection.
	e	Intrauterine Adhesiolysis	Data from RCTs suggest that, absent the use of adjuvant therapy, the risk of recurrence of IUAs following adhesiolysis is 53% (95% CI: 23–82%; two studies, *I^2^*=92%, good evidence quality). While data were less robust and included all study types, the risks of mild, moderate, and severe adhesions are 25% (95% CI: 2–60%; three studies, *I*^2^ = 93%, fair to good evidence quality), 25% (95% CI: 18–34%; three studies, *I*^2^ = 0%, fair to good evidence quality), and 5% (95% CI: 0–27%; three studies, *I*^2^ = 92%, fair to good evidence quality), respectively.	In addition to the use of hysteroscopy as the ‘gold standard’ assessment of adhesion outcomes, research designs should include both baseline and follow-up adhesion severity utilizing a standardized scoring method. It should also include comparisons of RF-based and cold mechanical techniques. Further studies investigating the value of repeat adhesiolysis as a strategy alone without adjuvant therapies should be undertaken. While it is understood that hysteroscopic assessment to determine the presence, location, and severity of IUAs should be the standard, appropriate intervals between adhesiolysis procedures have not yet been determined and should be investigated. Studies should report on the incidence of incomplete adhesiolysis.
4	**Comparative effectiveness of procedural techniques**			
	Any uterine procedure that violates the integrity of the basilar endometrium can result in IUA formation. Many procedural variables may affect the extent of trauma to the basalis and the degree of subsequent repair. The therapeutic role and prognostic value of early post-procedure interventions are unclear, including strategies such as the reinstallation of gels, the extended use of barriers (prolonged ‘residence’ time), or even the use of early second-look hysteroscopy.			Understanding the role of procedural techniques in the prevention and mitigation of endometrial trauma that leads to IUAs will require elucidation with appropriately designed investigation. Variables to consider include the value of early second-look hysteroscopy, the utility of prolonged residence time, and/or reinstillation or replacement of a given barrier. Preferably using RCT designs, there is a need for studies that clearly define disease/disorder phenotype, comparing surgical strategies, dissection techniques, and instrumentation for their impact on measurable surrogate outcomes for endometrial damage, as reflected in: - IUAs incidence and severity using a consensus-based system. - Menstrual outcomes. - Pregnancy rates. - Obstetrical outcomes. - Neonatal and pediatric outcomes.
	a	Removal of POC—early pregnancy	Aside from some limited studies comparing suction D&C to hysteroscopically directed removal of RPOC, there are extremely limited data on differences in surgical technique-related adhesiogenesis based on, for example, energy-based versus mechanical transection, dissection, or morcellation devices. Also absent are differences in meaningful outcomes, such as pregnancy rates and the incidence of adverse pregnancy-related outcomes.	Hysteroscopically-directed removal of products of conception compared to suction dilation and curettage, with and without ultrasound guidance, should continue to be investigated with randomized trials if possible and in concert with evaluation of adjuvants.
	b	Removal of retained POC—postpartum	Removal of RPOC post-partum presents several challenges that are different from early pregnancy cases. There are inadequate studies to inform meta-analysis and resulting guidance for clinicians, reflecting that study design and execution present challenges.	This category is challenging and can include a spectrum of clinical conditions including retained placenta in the delivery room, to delayed diagnosis weeks later. This will mean careful distinction of these different clinical situations since the appropriate interventions and mitigating strategies may vary. As with removal of RPOC in early pregnancy, hysteroscopically directed removal of products of conception compared to suction dilation and curettage, with and without ultrasound guidance, should continue to be investigated with randomized trials if possible and in concert with evaluation of adjuvants. When measures designed to assess menstrual function are adequately defined and validated, they should be included in the outcomes.
	c	Removal of a uterine septum	The review did not find studies that adequately controlled for septum classification, surgical strategy (transection versus resection), or technique (energy-based versus mechanical).	Studies should be designed based on: - Septum phenotype, e.g. CONUTA classification. - Strategy: transection versus resection. - Technique: energy based versus mechanical. Studies should report on the incidence of incomplete septum removal.
	d	Myomectomy	While some studies identified evaluating the impact of multiple myomectomy, including simultaneous removal of opposing leiomyomas, they were insufficient for meta-analyses. While multiple myomectomy can be staged for those lesions accessible hysteroscopically, such an approach does not feasibly apply when abdominal approaches are necessary, regardless of laparoscopic or laparotomic technique. Regardless, the leiomyoma phenotype (including size and location with respect to the endometrial cavity) are important variables in the design of comparative effectiveness research.	Studies should be designed based on: - Leiomyoma phenotype: size, number, FIGO system, Lasmar system. Denotation when multiple and when opposing or non-opposing. - Strategy: Pseudocapsule dissection technique, and single-step versus staged procedure. - Dissection technique: energy-based versus mechanical. - Removal technique: energy-based versus electromechanical. - Specimen extraction: spontaneous (residual tissue left in situ) or removed under direct visualization. Studies should report on the incidence of incomplete myoma resection. - For abdominal myomectomy (laparoscopic, laparotomic), the approach to endometrial repair should be reported (defect size, suture technique e.g. through endometrium or apposition by myometrial repair), along with suture type and caliber.
	e	Intrauterine adhesiolysis	No well-designed studies (RCTs) comparing mechanical to electrosurgical transection were found. However, our proportional meta-analysis, with all its limitations, suggested that there may not be a difference, particularly if RF energy is carefully applied and collateral thermal injury is minimized.	Studies should be designed based on: - IUA phenotype: consensus classification system for location, type, and extent. - Transection technique: mechanical, radiofrequency, or combined. - Adjuvant therapy versus no adjuvant: see below. Studies should report on the incidence of incomplete adhesiolysis.
5	**Comparative effectiveness of adjuvants**			
	a	Removal of POC—early pregnancy	Data from RCTs suggests that where surgical removal of POC is indicated in early pregnancy loss, there is evidence that intrauterine gels may reduce the presence and severity of IUAs compared to no gel (0.45; 95% CI: 0.30–0.68; three studies, *I*^2^ = 0%, poor to good evidence quality). However, there are inadequate data to determine the relative contribution of surgical techniques when compared to the adjuvant therapy, both on surrogate outcomes like IUA rate and severity and on relevant pregnancy-related outcomes.	It will be essential to design and execute comparative post-surgical adjuvant studies that consider relevant covariates (evacuation technique, POC volume) and the range of important outcomes, including IUAs, endometrial, and pregnancy outcomes. It should be remembered that the studies evaluating estrogen and progestin hormonal adjuvants were performed in women not recently pregnant, whereas in the context of a recent pregnancy and following delivery of a baby and placenta, women have circulating estradiol levels that are typically low.
	b	Removal of retained POC—post partum	There are inadequate studies to inform the best approach to removing RPOC following delivery. A challenge will be in the timing of intervention given that both hysteroscopically directed removal of RPOC as well as placement of an intrauterine adjuvant may be technically challenging shortly after obstetrical delivery.	Future research will require attention to all the issues raised for removal of POC in early pregnancy (as noted above) in addition to the challenges posed by the clinical context. In addition to surgical technique, the design of adjuvants (particularly barrier methods) will require creativity. The outcomes will be those as detailed above.
	c	Removal of a uterine septum	Data from RCTs suggests that there is a wide range of risk of de novo IUA formation following the removal of a septum and that gel barriers substantially reduced this risk (0.29; 95% CI: 0.12–0.69; three studies, *I*^2^ = 0%, fair to good evidence quality). It is unclear why there is such an overall difference in risk, but the septum category and removal strategy (resection versus transection) are likely candidates and must be considered in the design of future research.	For the various adjuvant categories, research designs should include septum phenotype, specification of technique (transection/division versus resection of the septum), and modality of correction (as either cold mechanical or RF-based).
	d	Myomectomy	The available high-quality studies evaluating the use of adjuvants for the primary prevention of de novo IUA formation after hysteroscopic myomectomy demonstrated a risk reduction with gel-based therapy of 0.38 (95% CI: 0.20–0.73; 3 studies, *I*^2^ = 0%, fair evidence quality). We found no suitable studies evaluating the utility of adjuvants following abdominal (laparoscopic, laparotomic) myomectomy.	For the various adjuvant categories, research designs should include myoma phenotype, specification of technique (pseudocapsule dissection), and modality of correction (as either cold mechanical or RF-based).
	e	Intrauterine adhesiolysis (secondary prevention)	Only two studies were found that directly compared an adjuvant therapy to no adjuvant (inclusive of antibiotics and/or estrogen-based hormonal therapies) following adhesiolysis; both were biodegradable type barriers. While our meta-analysis showed a reduction in risk, it did not reach significance. Amongst RCTs with study arms evaluating the impact of various adjuvant therapies for secondary prophylaxis of IUA recurrence, we identified three adjuvant subgroups (IUD, biodegradable barriers, and balloons) suitable for proportional meta-analyses. IUA recurrence was the lowest among patients treated with biodegradable barriers, with an overall rate of 28% (95% CI: 4–62%; three studies, *I*^2^ = 94%, good evidence quality). The recurrence rate was 43% for both intrauterine balloon (95% CI: 35–51%; 14 studies, *I*^2^ = 85%, poor to good evidence quality) and IUD (95% CI: 27–59%; four studies, *I*^2^ = 85%, fair to good evidence quality) adjuvant therapies post adhesiolysis. We encountered many differences in adjuvant study designs, including the type of adjuvant (mechanical barrier, gel-based barriers, tissue barriers, etc), residence time of the barrier, and frequency of reapplications. While the use of estrogen and progestin adjuvants was nearly ubiquitous, a meta-analysis of two RCTs demonstrated that systemic estrogen and progestin-based post-surgical adjuvant therapy does not change the risk or severity of post-adhesiolysis IUAs. Study outcomes were typically adhesion recurrence and, in some instances, severity, but relatively few investigated endometrial surrogates like thickness or other outcomes like glandular number, and molecular/receptor/genetic expressions are in their infancy. The use of growth factors and stem cells seems to show promise, but the literature is inadequate to adequately assess utility.	Evaluating the efficacy of adjuvants, including barriers, will require research designs that consider potential confounders that include dissection/transection surgical techniques, including RF-based or cold mechanical methods. For intrauterine adjuvants, in addition to the construct of the adjuvant itself, the residence time in the endometrial cavity should also be controlled. As noted above, the impact on measurable endometrial outcomes, including adhesion severity, should be performed consistently and carefully, and fertility and pregnancy-related outcomes, including the rates of peripartum adverse outcomes must be considered.
6	Endometrial outcomes		In this review, the most reported endometrial outcome was endometrial thickness compared to baseline as well as to cohorts of individuals with no known history of IUA. However, it was evident that endometrial thickness was inconsistently or inadequately presented, particularly with respect to technique and cycle timing. There were some early attempts to identify other endometrial markers, including gland openings, but we found no examples of other measures of endometrial function.	Endometrial outcomes should be further refined and developed, considering not only imaging and endometrial thickness but other indicators of intact structure and function. Future study designs should carefully consider cycle timing and the impact of endogenous versus exogenous estradiol, progesterone, phosphodiesterase inhibitors, and any other adjuvants that may be introduced in the post-adhesiolysis phase. Objective, standardized methods of reporting menstrual outcomes should be developed, validated, and utilized in surgically induced endometrial trauma research. Methods for evaluating the utility of local and/or systemic growth factors and stem cells will require development and validation.
7	Fertility outcomes		While many studies report fertility, there is a great deal of variation in the consideration of a host of potentially relevant outcomes. It is often difficult to determine the denominator (how many were trying to conceive) and the relative contributions of methods of conception and implantation— e.g. spontaneous, ovulation induction with or without insemination, IVF and embryo transfer, and the frequency of such interventions in each patient. Unfortunately, even the method of diagnosing pregnancy is not standardized.	Studies relating to fertility following intrauterine surgery must have an adequate duration of follow-up to identify meaningful outcome data, preferably multiple years following interventions. Research designs should include data on method(s) of conception/implantation and pregnancy detection. In addition to specifying the rates of ongoing pregnancy ≥20 weeks’ gestation, peripartum and neonatal outcomes should be included, as noted below.
8	Pregnancy Outcomes, including Obstetrical		It is apparent that in addition to adverse early pregnancy outcomes related to endometrial trauma and IUAs, there is a substantial risk of adverse outcomes later in pregnancy. However, comparative outcomes based on surgical technique and adjuvants are lacking. Our proportional meta-analyses demonstrated that, without the use of adjuvants, the rate of IUGR was 13% (95% CI: 4–24%; two studies, *I*^2^ = 36%, fair evidence quality), placental abruption was 3% (95% CI: 0–18%; two studies, *I*^2^ = 90%, fair evidence quality), PROM was 9% (95% CI: 0–32%; three studies, *I*^2^ = 94%, fair evidence quality), preterm delivery was 17% (95% CI: 7–28%; seven studies, *I*^2^ = 90%, fair evidence quality), peripartum hemorrhage was 16% (95% CI: 6–28%; five studies, *I^2^*=84%, fair evidence quality), PAS was 15% (95% CI: 5–28%; six studies, *I*^2^ = 87%, fair evidence quality), placenta previa was 11% (95% CI: 5–18%; four studies, *I*^2^ = 61%, fair evidence quality), hysterectomy was 3% (95% CI: 0–9%; two studies, *I*^2^ = 0%, fair evidence quality), and stillbirth was 0% (95% CI: 0–2%; four studies, *I*^2^ = 10%, fair evidence quality). These adverse outcomes, if accurate, burden women, their families, and the healthcare system(s) at large. Notably, we found the rates of these adverse obstetrical outcomes to be significantly lower among patients who achieved pregnancy after hysteroscopic adhesiolysis with the use of adjuvant therapies for secondary prophylaxis. The pooled estimated rate of preterm delivery in the adjuvant therapy group was 11% (95% CI: 0%-16%; 15 studies, *I*^2^ = 77%, fair to good evidence quality), compared to 17% post hysteroscopic adhesiolysis alone; the rate of PAS was 8% (95% CI: 3%-15%; 12 studies, *I*^2^ = 85%, poor to good evidence quality), compared to 15% post adhesiolysis alone; the rate of placenta previa was 2% (95% CI: 1%-4%; 10 studies, *I*^2^ = 0%, poor to good evidence quality), compared to 11% post adhesiolysis alone; the rate of peripartum hysterectomy was 1% (95% CI: 0%-4%; 5 studies, *I*^2^ = 34%, poor to good evidence quality), compared to 3% post adhesiolysis alone; and finally the rate of peripartum hemorrhage was 8% (95% CI: 3%-16%; 7 studies, *I*^2^ = 80%, poor to good evidence quality), compared to 16% post adhesiolysis alone.	The research design of any intervention should consider the following pregnancy-related outcomes throughout the entirety of pregnancy (from conception through delivery and postpartum course): - Fetal outcomes e.g. ectopic pregnancies, miscarriages. - Obstetrical outcomes, e.g. preterm labor, rupture of membranes, antepartum hemorrhage, placental abruption, placenta previa, and placenta accreta spectrum disorders. - Peripartum outcomes, e.g. peripartum hemorrhage and retained placenta. - Neonatal outcomes, e.g. fetal growth restriction and preterm delivery. - Pregnancy rates should be clearly defined to include those achieved via IVF/ART as well as include the percentage of patients that were trying to conceive within the overall cohort of evaluable subjects. - Rates, causes, and interventions required for the management of peripartum complications (e.g. blood transfusions and hysterectomy). - A national or international registry of patients conceiving following adhesiolysis might provide the optimal forum to build an evidence base regarding outcomes related to IUAs.
9	Classification system: we need a single consensus-based system.		In this review, there were several classification systems used in various studies, including some studies that utilized their own devised system. Such a circumstance impairs the comparative analysis process, including meta-analysis, and, consequently, progress in this crucial area of research.	There is a need for a single, consensus-based classification system for endometrial trauma and IUAs that considers all the known variables. Such a system should be able to be revised, as appropriate, to consider variables not yet well understood. Such an approach should be convened under the aegis of one or more internationally recognized and respected organizations, with participation by international and social stakeholders.
10	Impact of ‘non-surgical’ uterine procedures on endometrial trauma risk.		Over the past 30 years, several image-guided (‘non-surgical’) procedures have been developed for a variety of uterine conditions and clinical circumstances. These include uterine artery embolization (UAE) and energy-based ablation of adenomyosis and leiomyomas using focused ultrasound/microwave or radiofrequency electrical energy. While not included in the scope of this work, it is apparent that some of these, in particular, UAE, have been associated with endometrial trauma and IUAs. While initially indicated only for those individuals who have foregone future fertility, there is evidence that pregnancy can be achieved following these procedures, making evaluation of the endometrial impact an important component of their evaluation.	As suggested above, it will be necessary to evaluate the frequency, severity, and intervention-related variables related to image-guided techniques and other uterine procedures that could be defined as ‘non-surgical’. The general approach should mirror that recommended for traditional surgical procedures, including endometrial, fertility, and pregnancy-related outcomes throughout gestation.
11	Core Outcomes Set (COS) for investigators:		Characterization of many disorders and the interventions designed to address them can be helped by the codification of a Core Outcomes Set (COS). It would be appropriate to design a COS providing investigational guidance for the pathogenesis and interventions designed to minimize endometrial trauma and its adverse impacts, including IUAs. Such an approach would provide guidance for investigators, research funding organizations, and the editors of the journals charged with evaluating and publishing the evidence generated from appropriately designed research.	As with a consensus classification system, constructing a COS should be performed under the aegis of an appropriate, internationally respected entity or entities with participation by all the relevant stakeholders. Such stakeholders include not only investigators and journal editors, but relevant national and world societies related to women’s reproductive health and include appropriately selected non-medical participants. A process for review and appropriate revision of the COS is an integral part of the process.

IUA(s), intrauterine adhesion(s); CONUTA, congenital uterine anomalies—the working group of the European Society of Human Reproduction and Embryology (ESRE) and the European Society for Gynaecological Endoscopy (ESGE); COS, Core Outcomes Set; D&C, dilation and curettage; FIGO, Fédération Internationale de Gynécologie et Obstétrique (In English, the International Federation of Gynecology and Obstetrics); *I*^2^, measure of heterogeneity of study designs; Lasmar system, a system designed to estimate the case success rate for hysteroscopic myomectomy; POC, products of conception; PROM, premature rupture of membranes; RPOC, retained products of conception; RCTs, randomized controlled trials; UAE, uterine artery embolization.

### Prevalence and incidence

#### Background prevalence

It is understood that the background prevalence of IUAs is difficult to ascertain given the impractical nature of using diagnostic hysteroscopy to evaluate large populations of asymptomatic individuals with no previous history of intrauterine surgery. While several studies reported data on the prevalence of IUAs amongst at-risk groups such as those with “infertility”, the group was heterogenous and included patients with different underlying factors, including patients with recurrent pregnancy loss, mullerian anomalies, submucous lesions such as polyps and leiomyomas, as well as a percentage with previously diagnosed intrauterine adhesions. Due to these inconsistencies, the studies were inadequate for quantifying IUA prevalence through meta-analyses. Despite these limitations, a remarkable study from Italy reported the hysteroscopic evaluation of 922 women with abnormal uterine bleeding but with a previous normal pregnancy and no history of intrauterine surgery and found IUAs of unknown severity in 21, or ∼2% (Valli *et al.*, 2001). While this was the only study identified, it was the study design that most likely reflects the background prevalence of IUAs in a group of parous women without a previous intrauterine surgical procedure (the authors were directly contacted to confirm the control patients had no prior history of uterine surgeries, as this was not directly stated in the manuscript).

#### IUAs following pregnancy-related intrauterine procedures

Pregnancy-related procedures for removal of POC, including RPOC, are frequently performed in gynecological practice, but the clinical context can vary substantially based on features such as the gestational age at the time of the pregnancy loss, the time between the end of pregnancy and the treatment of POC, and the acuity of the presentation.

Of the 12 studies evaluating the incidence of IUAs after pregnancy-related intrauterine procedures, none reported the incidence specifically after second-trimester interventions. The overall incidence of IUAs after removing POC in the first trimester was 17% (95% CI: 10–26%; 13 studies, *I*^2^ = 87%, poor to good evidence quality) when including all study types, while the incidence was found to be 30% (95% CI: 15–48%; three studies, *I*^2^ = 83%, poor to good evidence quality) in studies utilizing an RCT design. Unfortunately, it was not possible to distinguish outcomes between scheduled and unscheduled (‘urgent’ or ‘emergent’) procedures, even though the circumstances and outcomes may differ for several reasons, including the specifics of the removal technique.

The overall incidence identified in this work is in line with the rate of 19% reported in a systematic review and meta-analysis of IUA risk following a first-trimester miscarriage (Hooker *et al.*, 2014). Previous miscarriage was a potential risk factor. D&C was used to treat most women, although the investigators were unable to determine whether curettage was performed by sharp, vacuum, or combined techniques. Repeat procedures were identified as the most critical risk factor for IUA formation.

No IUAs were encountered in women after a spontaneous miscarriage without any surgical intervention or in women treated medically, implying that D&C should be prevented as much as possible. Expectant and medical management should be discussed as appropriate alternatives for preventing D&C procedures and the basilar endometrial trauma that frequently results in adhesion formation. When the clinical circumstances make surgical removal of POC, including RPOC, a necessity, the unnecessary trauma potentially associated with sharp curettage should be avoided.

We did find evidence supporting the use of gel-based barriers following blind removal of POC. Application of hyaluronic acid following D&C for miscarriage appears to significantly reduce the risk of IUA formation (RR, 0.42 (95% CI: 0.25–0.72; two studies, *I*^2^ = 0%, poor to good evidence quality) (Hooker *et al.*, 2017; Vatanatara *et al.*, 2021; Sroussi *et al.*, 2022).

For patients undergoing surgical intervention for RPOC following vaginal or cesarean delivery, two studies demonstrated an IUA incidence of 24% (95% CI: 15–34%; two studies, *I*^2^ = 16%, fair evidence quality). Of note, there was heterogeneity in technique; in one study, the retained products were managed by blind D&C (Westendorp *et al.*, 1998) while in the other by hysteroscopically directed removal (Barel *et al.*, 2015).

It has been hypothesized that hysteroscopically directed removal of RPOC would reduce surgical trauma and related adhesions. We found only a single RCT comparing hysteroscopic to any blind technique for removing RPOC, thereby precluding meta-analysis. In this trial, the IUA incidence was 14.3% in the hysteroscopic cohort and 20.6% for those undergoing ultrasound-directed vacuum curettage, a difference that was not significant (Wagenaar *et al.*, 2023). These data differ from retrospective studies that have demonstrated variable rates and severity of postoperative adhesions comparing hysteroscopic and vacuum curettage (Rein *et al.*, 2011), one of which showed no differences in subsequent pregnancy outcomes (Wagenaar *et al.*, 2024). A systematic review comprising five cohorts that evaluated long-term complications and reproductive outcomes in women with suspected RPOC that were surgically or medically treated demonstrated D&C to be associated with a higher incidence of IUAs and incomplete removal when compared to a hysteroscopically directed technique (Hooker *et al*., 2016). Such evidence begs the performance of further well-designed prospective studies comparing these techniques.

#### IUAs following myomectomy

The results of our review demonstrate that the endometrial trauma associated with hysteroscopic myomectomy is associated with an increased risk of IUAs. A proportional meta-analysis of the eight identified comparative studies demonstrated that, without using adjuvants, the overall risk of de novo IUAs was 16% (CI: 6–28%; eight studies, fair to good evidence quality). We did find evidence that surgical barriers reduce the incidence of IUAs following hysteroscopic myomectomy (RR: 0.38; 95% CI: 0.20–0.73; three studies, *I*^2^ = 0%, fair evidence quality). However, we could not identify enough RCTs for meta-analysis evaluating potentially important factors contributing to this finding, such as dissection techniques, FIGO type, and leiomyoma number and volume.

Our review also found evidence suggesting that submucous myomectomy via an abdominal approach, either laparoscopic or laparotomic, may enhance the risk of IUAs (Gupta *et al.*, 2013; Bortoletto *et al.*, 2022), but there is relatively little evidence on potentially relevant variables, including breaching of the endometrium, removal of opposing submucous tumors, and the use and type of suture used, if any, to directly reapproximate the endometrium.

Especially given the prevalence of submucous leiomyomas and the need to design and deploy interventions that maintain or enhance fertility, it seems prudent to develop research protocols that provide us with better guidance on management techniques that minimize basilar endometrial trauma and the attendant risk of IUAs.

Our review did identify some clues that could inform the design and conduct of future studies. Comparative studies showed a strong relationship between IUAs and multiple simultaneous hysteroscopic myomectomies (Ikemoto *et al.*, 2022) when the leiomyomas lie opposite to each other (opposing) within the endometrial cavity, a finding supported by non-randomized but comparative studies (Yang *et al.*, 2008; Takasaki *et al.*, 2023). Such findings are consistent with the long-held but unproven notion that, in such instances, to optimize fertility, and without an effective method for preventing IUAs, hysteroscopic myomectomies of opposed myomas should be staged to allow healing of one endometrial surface before removing the tumor on the opposite side. While feasible in the hysteroscopic environment, especially given the low surgical morbidity and cost, such a strategy is much more difficult when an abdominal approach is necessary. Other outcomes of interest would include the influence of leiomyoma volume, number, and FIGO type. Another potentially relevant variable is the myomectomy technique itself, including, where needed, dissection techniques for those lesions involving the myometrium, including attention to the pseudocapsule and the tools used for such dissection, especially energy-based versus pure mechanical (‘cold’) dissecting instruments.

The post-intervention rate of IUA formation, or reformation, is a surrogate for the actual outcomes of interest that include fertility and pregnancy outcomes, including preterm labor and the risk of adverse peripartum events such as PAS, postpartum hemorrhage, and hysterectomy. It is understood that trials of this type are challenging to design, implement, and analyze. Still, such data are necessary to provide the evidence base necessary to inform clinicians, patients, and even payors, be they private or those representing government-funded national healthcare systems.

#### IUAs following hysteroscopic metroplasty for uterine septa

The results of our review demonstrate that endometrial trauma associated with hysteroscopically directed correction of intrauterine septae is associated with an increased risk of IUAs. Analyses of the eight non-adjuvant arms revealed a pooled incidence of 28% (CI: 13–46%; eight studies, *I*^2^ = 91%, fair to good evidence quality). However, there are issues relating to the varying phenotypes of such septae and the techniques that may substantially influence the risk and severity of the intrauterine trauma that underlies the postoperative IUA development. We were unable to find any guidance on postoperative adhesion risk based upon any of the septum classification systems, nor were there any analyzable data on the techniques of septum removal, including transection versus resection or ‘cold’ versus energy-based techniques. From an intervention perspective, our meta-analyses suggest that gel-based barriers can reduce the risk of IUAs post-septum removal (RR: 0.29; 95% CI: 0.12–0.69; three studies, *I*^2^ = 0%, fair to good evidence quality).

While septum correction itself is a technique that is under review for its appropriate indications, especially absent a history of recurrent first-trimester pregnancy loss (Krishnan *et al.*, 2021; Rikken *et al.*, 2021; Carrera *et al.*, 2022), it will undoubtedly continue to have a role for many women. Consequently, it will be essential to address the evidence gap regarding the septum phenotype and the strategies and techniques used for correction, including transection versus resection, the surgical endpoint of dissection, and the instrumentation used to perform the procedure (Unanyan *et al.*, 2022). Furthermore, the role of intrauterine barriers in these varying clinical and procedural circumstances requires clarification with the evidence resulting from appropriately designed comparative studies.

### Endometrial repair and IUA recurrence following hysteroscopic adhesiolysis

The formation of IUAs seems to be the ultimate result of a process thought to include an abnormal response to inflammation, resulting in increased extracellular matrix production. The inflammation not only directly damages the endometrium but also releases factors that stimulate fibrosis (Hooker *et al.*, 2022). The risk of recurrent IUA formation after hysteroscopic adhesiolysis can be attributed to a few factors, at least theoretically. These include the baseline extent of damage to the underlying basalis layer, the surgical technique used, the adequacy (‘completeness’) of adhesiolysis, the presence or absence of infection (i.e. endometritis), the extent of apposition of the denuded surfaces of the endometrial cavity, and the availability of the stem cells and other factors necessary for the process of functional basilar endometrial repair. There is evidence to suggest that inflammation, such as infection during repair post-injury, is associated with increased risks of endometrial fibrosis, resulting in a greater likelihood of adhesion recurrence and poor reproductive outcomes (Liu *et al.*, 2019b).

The extent of damage to the underlying basalis is, at least currently, challenging to evaluate in each individual but is probably a key factor contributing to both the development of IUAs and, even if adhesiolysis is performed, may be a major contributor to adverse pregnancy outcomes, including those that relate to abnormal placentation. The use of stem cells to regenerate the basalis layer has been studied in the last 15 years. However, there are few randomized trials with clinically relevant outcomes, including the achievement of term delivery and the rate of adverse events such as peripartum hemorrhage. For example, a recent meta-analysis of stem cell therapy included 10 studies, 2 of which were reports of the same population years apart, and 1 study had just one patient. None of these studies met our inclusion criteria, and, therefore, we cannot comment on the effectiveness of stem cell therapy for facilitating basilar endometrial repair and inhibiting IUS reformation following adhesiolysis (Chen *et al.*, 2022).

The most appropriate surgical instruments and techniques for hysteroscopic adhesiolysis have long been debated. Experts have argued that using ‘cold’ hysteroscopic scissors, thereby minimizing or, preferably, avoiding energy-based dissection, is essential to prevent or reduce further damage to the basilar endometrium and adjacent stem cells because of the perceived extent of collateral thermal injury. However, in this review, our pooled data comparison did not suggest differences in adhesion-related outcomes based on mechanical or energy-based transection. A more objective and comparative analysis of this variable was challenging because no study used the surgical modality as the primary endpoint for comparison, and more importantly, a head-to-head comparison between the use of energy and cold dissection with scissors was not performed in any study.

The adequacy of adhesiolysis is likely another important factor contributing to successful fertility- and pregnancy-related outcomes, but it is one that was difficult to evaluate in this review. It seems reasonable to assume that suboptimal normalization of the endometrial cavity would be associated with suboptimal clinical outcomes. However, most studies were performed with follow-up (‘second-look’) hysteroscopy and, if necessary, additional adhesiolysis until the cavity was ‘normalized’; no studies compared optimal to suboptimal adhesiolysis. Consequently, no conclusions from the studies included in this systematic review can be made about this variable.

Early post-adhesiolysis second-look hysteroscopy may itself have a role in interruption of the pathogenesis of recurrent IUAs. Early hysteroscopic assessment allows the detection and treatment of reforming IUAs in their early stages, thereby interrupting and potentially avoiding consolidation of the adhesive process (Panayotidis *et al.*, 2009). Whether there are other means by which this process may be interrupted is unclear. However, we did see examples of studies that intermittently dilated balloons (Shi *et al.*, 2019) or reapplied gels (Mao *et al.*, 2020) at some period following the adhesiolysis.

Separating the surfaces of the endometrial cavity with barriers positioned immediately following adhesiolysis has been the subject of many studies evaluating a spectrum of biodegradable barriers and intrauterine devices. Ideally, the first step would be a comparison of adjuvant use of a given intervention following adhesiolysis to no adjuvant, measuring the rate and severity of adhesion recurrence and clinically relevant pregnancy outcomes, including those related to obstetrical delivery, the peripartum experience, and the neonate, especially those born prematurely. The second step would involve comparing individual adjuvants with each other, alone or in combination, to identify the optimal approach.

In this review, we were able to find only two RCTs that met our criteria with which to perform comparative analyses of post-adhesiolysis recurrence with and without adjuvant therapies. While both utilized biodegradable barriers, the two barriers were not identical; one employed a gel containing hyaluronic acid (Acunzo *et al.*, 2003) and the other a hydrophilic biodegradable film (Fernandez *et al.*, 2024). We determined that the design and residence time of the two barriers (days to a week) were similar enough to allow meta-analysis that showed a non-significant decrease in the adhesion recurrence rate. Adding the Mao *et al.* (2020) study that had been excluded did not materially change this result.

Given the plethora of randomized studies of adjuvants for secondary prevention that did not have ‘no adjuvant’ arms, we further analyzed pooled results of the RCTs. This approach, while potentially informative, has many inherent limitations. The pooled estimates for randomized trials suggested the rate of IUA recurrence without adjuvants to be 35% (95% CI: 24–46%; 13 studies, *I*^2^ = 95%, poor to good evidence quality) and to be 28% (95% CI: 4–62%; three studies, *I*^2^ = 94%, good evidence quality) following the use of biodegradable barriers. Notably, our pooled rate of recurrence rates with intrauterine balloon was 43% (95% CI: 35–51%; 14 studies, *I*^2^ = 85%, poor to good evidence quality).

One can only speculate on the rationale behind the apparent difference between biodegradable barriers and balloon adhesion-related outcomes suggested by these pooled data. While gels theoretically distribute to all regions of endometrial cavities that have infinite individual variations in shape, size, and contour, the area of the cavity occupied by a balloon may be somewhat limited by the design, regardless of the ovoid, heart, or triangular shape of the device. It is also possible that the removal of the balloon itself can promote injury and recurrence of the adhesions; gels are degraded and/or expelled over time and do not require removal.

Examination of the studies demonstrates a substantial degree of heterogeneity that, in part, reflects differences in study design, including variable volumes of gel and shapes and sizes of balloons and their resident time post-surgery. In some instances, balloons were inserted more than once, and gels were reapplied up to a week after the original use of the adjuvant agent. To make reasonable and informed conclusions, there is a need for actual comparative effectiveness studies of these adjuvants using similar study designs.

A criticism of our methodology could be our decision to disregard gonadal steroids and antibiotics as variables for analysis. There were two general reasons for this decision. One is that using such agents was so common that they were essentially considered standard of care. Considering them as independent variables would preclude or severely limit many of the analyses performed. This reason alone would not justify their exclusion. However, we found two RCTs that evaluated barriers with and without the use of estrogen-based adjuvants, with or without a progestin, suggesting that such adjuvants have no impact on the recurrence rate and severity of adhesions following hysteroscopic adhesiolysis (AAGL-ESGE, 2017; Yang *et al.*, 2022b; Hanstede *et al.*, 2023). Indeed, Hanstede *et al.* also demonstrated no difference in pregnancy and obstetrical outcomes in the 3 years following the adhesiolysis. Consequently, we decided to include control groups with or without antibiotics or gonadal steroid administration and the control arms in studies that used no such adjuvants.

### Pregnancy outcomes among patients following adhesiolysis

In this review, we demonstrate that while treating IUAs with adhesiolysis can normalize the appearance of the endometrial cavity, the underlying damage to the endometrium remains a factor that may adversely impact subsequent obstetrical outcomes. For the vast majority of patients, the ultimate goal of treating IUAs is not only to conceive but also to complete a pregnancy that results in both a healthy infant and mother. Of the subset of studies that included information on pregnancy complications, we identified high rates of preterm delivery and placental abnormalities, including PAS, likely contributors to the increased risk of peripartum hemorrhage and hysterectomy. We estimate the pooled rate of preterm delivery among patients who achieved pregnancy post-hysteroscopic adhesiolysis to be 17% (95% CI: 7–28%; seven studies, *I*^2^ = 90%, fair evidence quality), which is nearly double the estimated global preterm delivery rate of 9.8% (Ohuma *et al.*, 2023). We found the rate of PAS to be 15% (95% CI: 5–28%; six studies, *I*^2^ = 87%, fair evidence quality) in pregnancies post adhesiolysis, whereas the estimated pooled global prevalence of PAS in pregnancies is much lower at 0.17%, though there is considerable heterogeneity in studies and the reported range is wide due to significant diagnostic challenges (Jauniaux *et al.*, 2019a). We determined the rate of placenta previa to be 11% (95% CI: 5–18%; four studies, *I*^2^ = 61%, fair evidence quality) among patients who achieved pregnancy post adhesiolysis, which again is comparatively much higher than the estimated pooled global placenta previa prevalence of 0.56% (Jauniaux *et al.*, 2019b). In addition, we determined the rate of peripartum hysterectomy to be 3% (95% CI: 0–9%; two studies, *I*^2^ = 0%, fair evidence quality), whereas, comparatively, the estimated global rate of emergent peripartum hysterectomy is only 0.11% (Kallianidis *et al.*, 2023).

Notably, we found the rates of these adverse obstetrical outcomes to be significantly lower among patients who achieved pregnancy after hysteroscopic adhesiolysis with the use of adjuvant therapies for secondary prophylaxis. The pooled estimated rate of preterm delivery in the adjuvant therapy group was 11% (95% CI: 0%-16%; 15 studies, *I*^2^ = 77%, fair to good evidence quality), compared to 17% post hysteroscopic adhesiolysis alone; the rate of PAS was 8% (95% CI: 3%-15%; 12 studies, *I*^2^ = 85%, poor to good evidence quality), compared to 15% post adhesiolysis alone; the rate of placenta previa was 2% (95% CI: 1%-4%; 10 studies, *I*^2^ = 0%, poor to good evidence quality), compared to 11% post adhesiolysis alone; the rate of peripartum hysterectomy was 1% (95% CI: 0%-4%; 5 studies, *I*^2^ = 34%, poor to good evidence quality), compared to 3% post adhesiolysis alone; and finally the rate of peripartum hemorrhage was 8% (95% CI: 3%-16%; 7 studies, *I*^2^ = 80%, poor to good evidence quality), compared to 16% post adhesiolysis alone. These results highlight the potentially significant personal and public health costs associated with IUAs and their treatment and should inform patient counseling. Moreover, the potential for obstetrical and neonatal complications should be considered by providers caring for patients with a history of prior intrauterine adhesiolysis. A limitation of our results is that only a subset of studies provided information on peripartum complications, with many studies reporting conceptions or even the number of live births without additional obstetrical details. Given the serious nature of the complications associated with pregnancy following adhesiolysis, such omissions are of concern, making it critical that future studies rigorously capture and report these outcomes.

Several questions remain regarding peripartum complications, and these questions constitute essential goals for future studies. Among these include whether there are patient-related factors identifiable before conception that would indicate the highest risk for adverse outcomes and whether there are treatment strategies that further mitigate risk. This information could add nuance to patient counseling and inform obstetrical care strategies. A national or international registry of patients conceiving following adhesiolysis might provide the optimal forum to build an evidence base regarding outcomes related to this condition. Finally, future studies should also seek to better understand the implications of IUAs and subsequent adhesiolysis for overall healthcare expenditures, including pregnancy and neonatal care, as such information could motivate additional funding and research targeting new strategies for prevention and treatment.

### Menstrual outcomes among patients following hysteroscopic adhesiolysis

Changes to menstrual function because of basilar endometrial trauma may be the only symptom experienced by affected women and can range from relative reductions in duration or volume of blood loss to amenorrhea. Our meta-analyses showed that hysteroscopic adhesiolysis with the use of adjuvant therapies is relatively effective in restoring menstrual function. However, we were unable to compare the effect of hysteroscopic adhesiolysis alone. As with other outcomes, limitations in study design and the lack of consistency in the IUA classification systems used in studies prevented the evaluation of menstrual outcomes based on adhesion phenotype.

### Assessing endometrial function among patients following adhesiolysis

EMT, as measured by transvaginal ultrasound, is often reduced in patients with IUAs, presumably reflecting the downstream impact of endometrial trauma on the functional capacity of the endometrium. The pathogenesis of this change may include fibrosis of the supporting arterial blood supply and depletion, or loss, of the endometrial stem cells located in the basilar endometrium and adjacent superficial myometrium (Hooker *et al.*, 2022). Individually or collectively, these and other less well-defined factors likely contribute to the reduction in fertility. Consequently, the measurement of EMT may be a reasonable surrogate for the capacity of the endometrium to support the establishment and maintenance of a successful pregnancy. We were unable to conduct a meta-analysis for the EMT outcome due to insufficient studies with comparable designs and sufficient rigor. However, the pooled results reported by this review indicate that a small degree of improvement, ∼0.4 mm, can be achieved by using a gel barrier following adhesiolysis compared to no adjuvant.

Menstrual cycle timing is critical when measuring EMT because of the gonadal steroid-related dynamic nature of endometrial growth across the menstrual phases and/or in relation to exogenous hormonal exposure (Craciunas *et al.*, 2019; Mao *et al.*, 2020). Consequently, research protocols should require EMT measurement at explicitly stated stages of the menstrual cycle or after a specified dose and duration of exogenous estrogen, progesterone, or other pharmaceutical exposure. Other issues to consider include antibiotic use and the prescription of phosphodiesterase inhibitors. In our review, we were able to identify only two studies that specified consistent timing for the measurement of EMT (Mao *et al.*, 2020; Wang *et al.*, 2020b), highlighting a need for additional studies that capture this outcome with sufficient rigor.

Questions regarding the reliability and predictive power of EMT measurement are not new (Craciunas *et al.*, 2019), and this circumstance has led to the search for other and more refined methods of evaluating endometrial function. Histological evaluation of the endometrium has been reported, specifically documentation of an increase in tubular glands following adhesiolysis (Mao *et al.*, 2020). Other markers being explored include endometrial pattern, endometrial volume, Doppler indices, endometrial wave-like activity, and various molecular expressions (Craciunas *et al.*, 2019). The development of well-designed and innovative approaches to evaluating endometrial function will improve the ability of investigators to meaningfully test the efficacy of various intrauterine procedures, techniques, and adjuvants.

## Conclusions and implications for practice

Patients undergoing uterine surgery involving the endometrial cavity are exposed to the risk of basilar endometrial trauma that is poorly healed with related IUAs, and this circumstance may be especially prevalent following pregnancy-related procedures. For many, these outcomes are asymptomatic or minimally symptomatic until they try to conceive, when infertility may be realized. While IUAs can be addressed with surgical transection, fertility challenges may persist, and even if pregnancy ensues, a variety of obstetrical and neonatal complications may occur. This latter point, typically experienced by those who have undergone adhesiolysis, reflects the fact that the adverse impact of endometrial damage can persist even after the successful treatment of adhesions. The goal of adjuvant therapies is to facilitate functional endometrial repair, thereby preventing the formation or, for adhesiolysis, the reformation of IUAs following intrauterine surgery. In this systematic review, we evaluated the evidence reporting the performance of these agents in several clinical contexts. While none performed as well as hoped, the data on gels indicate that these agents may be superior to other barriers, such as balloons and IUDs, when IUA incidence or recurrence is the outcome. However, study designs precluded adequate evaluation of many of the techniques and adjuvants, and outcomes related to IUA severity, infertility, and pregnancy were largely impossible to analyze satisfactorily.

Where data were insufficient to make conclusions, the literature review and, where feasible, meta-analyses serve to identify gaps in our knowledge of the associations between certain procedure types, IUA formation, and the clinical outcomes of interest that particularly relate to infertility and adverse obstetrical and even neonatal outcomes. These represent areas wherein more intensive but carefully executed research and development are required. We further describe these issues in the summary and needs assessment (Table 2).

The concept of enduring, traumatically induced damage to the basilar endometrium has several implications. Prevention or minimization of such trauma should be a goal that may be achieved by avoiding surgery where appropriate, by using techniques that minimize surgical trauma, and by designing adjuvants that facilitate complete and functional repair of the basilar endometrium following surgery. It also means that reliable measures of endometrial function should be developed and then validated to be used as outcomes in trials of techniques and devices designed to address this somewhat vexing clinical problem. Finally, it is apparent that a standardized classification system and a set of core outcomes are glaring unmet needs that will have to be addressed to optimize future research and development.

## Supplementary Material

dmaf019_Supplementary_Data

## Data Availability

The data extracted and used for this study are on file and available upon request to the corresponding authors.
