# Supplementary material for: The epidemiology, clinical burden, and prevention of intrauterine adhesions (IUAs) related to surgically induced endometrial trauma: a systematic literature review and selective meta-analyses
Source: Hum Reprod Update. 2025 Sep 7;31(6):588–625. doi: 10.1093/humupd/dmaf019 (PMC12584913; doi:10.1093/humupd/dmaf019)
Supplement: dmaf019_Supplementary_Data [file dmaf019_supplementary_data.pdf]

Title: The epidemiology, clinical burden, and prevention of intrauterine adhesions (IUAs) related to surgically induced endometrial trauma: a systematic literature review and selective meta-analyses

Running title: Surgically Associated Endometrial Trauma and IUAs

Malcolm G. Munro[1, 2] *, Christina A. Salazar[2.3]*, Bala Bhagavath[2.4], Mark H. Emanuel, PhD[2.5], Heather G. Huddleston[2,6], Dhruv Sobti[7], Ajit K. Jaiswal[7], Rachel Gamburg[7], Jatinder Kumar[7], Coby Martin[7], and Angelo B. Hooker[2.8]., for the Women's Health Research Collaborative's Endometrial Trauma Group.

*Co- First Author

[1]David Geffen School of Medicine at UCLA, University of California Los Angeles, Department of Obstetrics & Gynecology, Los Angeles, California, USA 90025.

[2]Women's Health Research Collaborative, New York, New York, USA
[3]University of Texas at Austin Dell Medical School, Department of Women's Health, Austin, Texas, 78712 USA.

[4]Department of Obstetrics & Gynecology, Division of Reproductive Endocrinology and Infertility, University of Wisconsin-Madison, Madison, Wisconsin, USA, 53706

[5]University Medical Center, Department of Gynecology and Reproductive Health, Utrecht, the Netherlands 3584 CX.

[6]Department of Obstetrics, Gynecology & Reproductive Sciences, Division of Reproductive Endocrinology and Infertility, the University of California, San Francisco, San Francisco, California, USA, 94143.

[7]Axtria Inc, Berkeley Heights, New Jersey, USA, 07922.
[8]Zaans Medical Center (ZMC), Department of Obstetrics and Gynaecology, Zaandam, the Netherlands, 1500 EE.

Table of contents
Supplementary Figure S1. Pooled results of the meta-analysis for the comparative effectiveness of adjuvant therapies versus no adjuvant for primary prevention of adhesions following hysteroscopic metroplasty for septa (RCT study design only).
Supplementary Figure S2. Pooled results of the meta-analysis for the comparative effectiveness of gel adjuvant therapies versus no adjuvant for secondary prevention of adhesions following potentially adhesiogenic procedures (RCT study design only).
Supplementary Table S1. PubMed database search and terms.
Supplementary Table S2. Embase database search and terms.
Supplementary Table S3. Studies that were excluded from the meta-analysis of the incidence of IUAs following hysteroscopic metroplasty for septa**.**
Supplementary Table S4. Studies that were excluded from the meta-analysis of the incidence of IUAs following hysteroscopic myomectomy.
Supplementary Table S5. Studies that were excluded from the meta-analysis of the incidence of IUAs following the removal of retained products of conception post-delivery (postpartum).
Supplementary Table S6. Studies that were excluded from the meta-analysis of the recurrence of IUAs among patients following adhesiolysis.
Supplementary Table S7. Studies that were excluded from the meta-analysis of primary prevention adjuvants.
Supplementary Table S8. Summary data on the studies included in the meta-analysis of primary prevention adjuvants.
Supplementary Table S9. Studies excluded from the meta-analysis of secondary prevention adjuvants.
Supplementary Table S10. Summary data on studies included for the meta-analysis of secondary prevention adjuvants.

Supplementary Table S11. Studies excluded from the meta-analysis of head-to-head comparisons of adjuvant therapies for secondary prevention.
Supplementary Table S12. Studies excluded from the meta-analysis of obstetrical outcomes among patients following adhesiolysis.
Supplementary Table S13. Summary data on studies included in the meta-analysis of obstetrical outcomes following hysteroscopic adhesiolysis with adjuvants**.**
Supplementary Table S14. Summary data on studies included in the meta-analysis of obstetrical outcomes following hysteroscopic adhesiolysis without use of adjuvants.
Supplementary Table S15. Studies excluded from the meta-analysis of menstrual outcomes among patients following hysteroscopic adhesiolysis.

Supplementary Figure S1

Pooled results of the meta-analysis for the comparative effectiveness of adjuvant therapies versus no adjuvant for primary prevention of adhesions following hysteroscopic metroplasty for uterine septa (RCT study design only)

	Adjuvant		Control			
Study	Events	Total	Events	Total	RR	95%-CI
**Hysteroscopic Metroplasty for Septa**						
Tafti 2021	4	34	12	31	0.30	[0.11; 0.84]
DiSpiezio 2011	0	6	3	7	0.16	[0.01; 2.63]
Tonguc 2010	5	44	1	35	3.98	[0.49; 32.50]
Guida 2004	1	8	3	8	0.33	[0.04; 2.56]
**Random effects model**		**92**		**81**	**0.50**	**[0.14; 1.77]**

Heterogeneity: $I^2 = 43.1\%$, $\tau^2 = 0.7779$, $p = 0.1530$

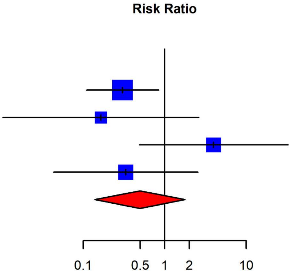

CI, confidence interval; IUAs, intrauterine adhesions; RCT, randomized controlled trial; RR, risk ratio; t, tau. Events are cases with intrauterine adhesions identified at second-look hysteroscopy.

Supplementary Figure S2

Pooled results of the meta-analysis for the comparative effectiveness of gel adjuvant therapies versus no adjuvant for secondary prevention of adhesions following potentially adhesiogenic procedures (RCT study design only).

Study	Adjuvant Events	Adjuvant Total	Control Events	Control Total	RR	95%-CI
**Adhesiolysis**						
Fernandez 2024	42	71	62	82	0.78	[0.62; 0.98]
Mao 2020	21	29	24	32	0.97	[0.71; 1.30]
Acunzo 2003	6	40	13	36	0.42	[0.18; 0.98]
**Random effects model**		**140**		**150**	**0.78**	**[0.53; 1.14]**

Heterogeneity: $I^2 = 46.2\%$, $\tau^2 = 0.0708$, $p = 0.1558$

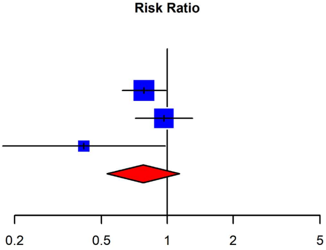

CI, confidence interval; IUAs, intrauterine adhesions; RCT, randomized controlled trial; RR, risk ratio; t, tau. Events are cases with intrauterine adhesions identified at second-look hysteroscopy.

Supplementary Table S1. PubMed database search and terms.

#	Search Terms	# Hits (16 Dec 2022)	# Hits (8 Nov 2024)
1	asherman[All Fields] OR intrauterine adhesions[All Fields] OR intrauterine synechiae[All Fields] OR (intrauterin*[All Fields] AND adhes*[All Fields]) OR "endometrial trauma"	2,137	2,498
2	adhesiolysis[Title/Abstract] OR synechiolysis[Title/Abstract] OR Versapoint[Title/Abstract] OR "Uterine Artery Occlusion" OR "Uterine Artery Embolization" OR "Uterine fibroid ablation" OR ((("laparoscopic myomectomy"[Title/Abstract] OR "robotic myomectomy"[Title/Abstract] OR "Laparotomic Myomectomy"[Title/Abstract] OR "Open Myomectomy"[Title/Abstract] OR "Abdominal Myomectomy"[Title/Abstract] OR Sonocision[Title/Abstract] OR Harmonic[Title/Abstract]) OR ("Focused Ultrasound"[Title/Abstract] OR HIFU[Title/Abstract] OR Sonata[Title/Abstract] OR Accessa[Title/Abstract] OR Halt[Title/Abstract])) AND (leiomyomas OR fibroid* OR myomas))	6,060	6,777
3	1 AND 2	263	312

4	(epidemiology[MeSH Terms] OR epidemiology[Title/Abstract] OR incidence[MeSH Terms] OR incidence[Title/Abstract] OR prevalence[MeSH Terms] OR Prevalence[Title/Abstract] OR mortality[Title/Abstract] OR death[Title/Abstract] OR morbidity[MeSH Terms] OR morbidity[Title/Abstract] OR morbidities[Title/Abstract] OR "risk factor"[Title/Abstract]) OR (fertility[Title/Abstract] OR infertility[Title/Abstract] OR reproduct*[Title/Abstract] OR IVF[Title/Abstract] OR "in vitro fertilization"[Title/Abstract] OR pregnancy[Title/Abstract] OR miscarriage[Title/Abstract] OR "live births"[Title/Abstract] OR menstr*[Title/Abstract] OR "embryo transfer" OR "assisted reproductive technologies"[Title/Abstract] OR "spontaneous abortion"[Title/Abstract] OR "recurrent abortion"[Title/Abstract] OR "recurrent pregnancy loss"[Title/Abstract] OR "placenta accreta"[Title/Abstract] OR "placenta increta"[Title/Abstract] OR "placenta accreta spectrum"[Title/Abstract] OR "endometrial thickness"[Title/Abstract] OR "postpartum hemorrhage"[Title/Abstract] OR "postpartum haemorrhage"[Title/Abstract] OR hypomenorrhea[Title/Abstract] OR hypomenorrhoea[Title/Abstract] OR hypermenorrhea[Title/Abstract] OR hypermenorrhoea[Title/Abstract] OR hypermenorrhea[Title/Abstract] OR hypermenorrhoea[Title/Abstract] OR amenorrhea[Title/Abstract] OR amenorrhoea[Title/Abstract])	4,279,591	4,796,301
5	economic*[Title/Abstract] OR cost*[Title/Abstract] OR pharmacoeconomic[Title/Abstract] OR price*[Title/Abstract] OR financ*[Title/Abstract] OR reimburs*[Title/Abstract] OR cost of illness[Title/Abstract] OR economic model*[Title/Abstract] OR cost benefit*[Title/Abstract] OR cost effect*[Title/Abstract] OR cost utili*[Title/Abstract] OR resource utili*[Title/Abstract] OR health utili*[Title/Abstract] OR expenditures[MeSH Terms] OR expenditure*[Title/Abstract] OR productivity[MeSH Terms] OR budget[MeSH Terms] OR budget*[Title/Abstract] OR readmission*[Title/Abstract] OR resource use*[Title/Abstract] OR claim*[Title/Abstract] OR diagnostic cost*[Title/Abstract] OR procedure cost*[Title/Abstract]	1,396,230	1,613,792

6	quality of life[Title/Abstract] OR QOL[Title/Abstract] OR Quality adjusted life year[Title/Abstract] OR qaly[Title/Abstract] OR QALY[Title/Abstract] OR Disease adjusted life year OR DALY[Title/Abstract] OR quality adjust*[Title/Abstract] OR utility index[Title/Abstract] OR utilities index[Title/Abstract] OR utility valu*[Title/Abstract] OR utilities valu*[Title/Abstract] OR health utilit*[Title/Abstract] OR HUI[Title/Abstract] OR adversit*[Title/Abstract] OR satisf*[Title/Abstract] OR patient reported outcomes[Title/Abstract] OR well-being[Title/Abstract] OR well being[Title/Abstract] OR burden[Title/Abstract] OR activities of daily living[Title/Abstract] OR daily living activit*[Title/Abstract]	1,109,087	1,323,151
7	guideline[MeSH Terms] OR guideline[All Fields] OR recommendation*[All Fields] OR consensus statement*[All Fields] OR standard of care[MeSH Terms]	559,367	631,699
8	unmet need[All Fields] OR unmet-need[All Fields] OR data gap*[All Fields] OR data-gap[All Fields]	14,167	18,142
9	#4 OR #5 OR #6 OR #7 OR #8	6,472,155	7,334,364
10	#3 AND #9	231	278
11	#10 AND (humans[Filter] AND English[Filter])	170	201
12	Published Since Search Date: December 16, 2022 [conducted November 8, 2024]	-	29

Supplementary Table S2. Embase database search and terms.

#	Search Terms	# Hits (16 Dec 2022)	# Hits (8 Nov 2024)
1	‘Asherman’ OR ‘intrauterine adhesions’ OR ‘intrauterine synechiae’ OR (‘intrauterin*’ AND ‘adhes*’) OR ‘endometrial trauma’	4,382	5,051
2	adhesiolysis:ab,ti OR synechiolysis:ab,ti OR Versapoint:ab,ti OR ‘Uterine Artery Occlusion’ OR ‘Uterine Artery Embolization’ OR ‘Uterine fibroid ablation’ OR (((‘laparoscopic myomectomy’:ab,ti OR ‘robotic myomectomy’:ab,ti OR ‘Laparotomic Myomectomy’:ab,ti OR ‘Open Myomectomy’:ab,ti OR ‘Abdominal Myomectomy’:ab,ti OR Sonocision:ab,ti OR Harmonic:ab,ti) OR (‘Focused Ultrasound’:ab,ti OR HIFU:ab,ti OR Sonata:ab,ti OR Accessa:ab,ti OR Halt:ab,ti) AND (leiomyomas OR fibroid* OR myomas)))	11,040	12,311
3	1 AND 2	510	589
4	(epidemiology/exp OR epidemiology:ab,ti OR incidence/exp OR incidence:ab,ti OR prevalence/exp OR Prevalence:ab,ti OR mortality:ab,ti OR death:ab,ti OR morbidity/exp OR morbidity:ab,ti OR morbidities:ab,ti OR ‘risk factor’:ab,ti) OR (fertility:ab,ti OR infertility:ab,ti OR reproduct*:ab,ti OR IVF:ab,ti OR ‘in vitro fertilization’:ab,ti OR pregnancy:ab,ti OR miscarriage:ab,ti OR ‘live births’:ab,ti OR menstr*:ab,ti OR ‘embryo transfer’ OR ‘assisted reproductive technologies’:ab,ti OR ‘spontaneous abortion’:ab,ti OR ‘recurrent abortion’:ab,ti OR ‘recurrent pregnancy loss’:ab,ti OR ‘placenta accreta’:ab,ti OR ‘placenta increta’:ab,ti OR ‘placenta accreta spectrum’:ab,ti OR ‘endometrial thickness’:ab,ti OR ‘postpartum hemorrhage’:ab,ti OR ‘postpartum haemorrhage’:ab,ti OR hypomenorrhea:ab,ti OR hypomenorrhoea:ab,ti OR hypermenorrhea:ab,ti OR hypermenorrhoea:ab,ti OR hypermenorrhea:ab,ti OR hypermenorrhoea:ab,ti OR amenorrhea:ab,ti OR amenorrhoea:ab,ti)	7,422,025	8,420,546
5	economic*:ab,ti OR cost*:ab,ti OR pharmacoeconomic:ab,ti OR price*:ab,ti OR financ*:ab,ti OR reimburs*:ab,ti OR ‘cost of illness’:ab,ti OR ‘economic model*’:ab,ti OR ‘cost benefit*’:ab,ti OR ‘cost effect*’:ab,ti OR ‘cost utili*’:ab,ti OR ‘resource utili*’:ab,ti OR ‘health utili*’:ab,ti OR expenditures/exp OR expenditure*:ab,ti OR productivity/exp OR budget/exp OR budget*:ab,ti OR readmission*:ab,ti OR ‘resource use*’:ab,ti OR claim*:ab,ti OR ‘diagnostic cost*’:ab,ti OR ‘procedure cost*’:ab,ti	1,811,793	2,084,098

6	‘quality of life’:ab,ti OR QOL:ab,ti OR ‘Quality adjusted life year’:ab,ti OR qaly:ab,ti OR QALY:ab,ti OR ‘Disease adjusted life year’ OR DALY:ab,ti OR ‘quality adjust*’:ab,ti OR ‘utility index’:ab,ti OR ‘utilities index’:ab,ti OR ‘utility valu*’:ab,ti OR ‘utilities valu*’:ab,ti OR ‘health utilit*’:ab,ti OR HUI:ab,ti OR adversit*:ab,ti OR satisf*:ab,ti OR ‘patient reported outcomes’:ab,ti OR ‘well-being’:ab,ti OR ‘well being’:ab,ti OR burden:ab,ti OR ‘activities of daily living’:ab,ti OR ‘daily living activit*’:ab,ti	1,578,860	1,856,800
7	guideline/exp OR guideline OR recommendation* OR ‘consensus statement*’ OR ‘standard of care’/exp	4,343,031	5,202,370
8	‘unmet need’ OR ‘unmet-need’ OR ‘data gap*’ OR ‘data-gap’	234,25	30,326
9	#4 OR #5 OR #6 OR #7 OR #8	12,046,045	13,811,268
10	#3 AND #9	445	520
11	#3 AND #9 AND humans/lim	423	497
12	#3 AND #9 AND humans/lim AND English/lim	403	474
13	Records added to Embase: December 16, 2022 – November 8, 2024	-	81

Supplementary Table S3. Studies that were excluded from the meta-analysis of the incidence of IUAs following hysteroscopic metroplasty for septa.

Study	Reason for exclusion
Shen 2022	This is a retrospective study that looked at patients who underwent septoplasty and at the incidence of IUAs diagnosed at the time of metroplasty. It was not a post metroplasty study
Xue 2018	This is a retrospective study that was excluded due to inadequate description of the methodology for the formation of the three groups. There is no group that can be considered as a “no adjuvant arm” due to incomplete reporting of the data.

Supplementary Table S4. Studies that were excluded from the meta-analysis of the incidence of IUAs following hysteroscopic myomectomy.

Study	Reason for exclusion
Ikemoto 2022, Weyers 2022, Sebbag 2019	These studies did not evaluate myomectomy patients treated without adjuvants, which was an exclusion criterion for this analysis.
Bhandari 2016	This study reports on post myomectomy IUAs however the results were not separated by approach and thus were mixed between laparoscopic and hysteroscopic approaches.
Taskin 2000	This study was noted to have inadequate methodology and inaccurate reporting. It was not possible to discern the incidence of IUAs following myomectomy in this study.

Supplementary Table S5. Studies that were excluded from the meta-analysis of the incidence of IUAs following the removal of retained products of conception post-delivery (postpartum).

Study	Reason for exclusion
Smorgick 2014	This study contained duplicate findings of the same population described in Barel 2015, which included more detailed reporting.

Supplementary Table S6. Studies that were excluded from the meta-analysis of the recurrence of IUAs among patients following adhesiolysis.

Study	Reason for exclusion
Trinh 2022, Mao 2020, Sendag 2013	These studies included subjects where second look hysteroscopy for IUA outcomes was not described in the methodology.
Jin 2021	This study was noted to have inadequate methodology for assessment of IUA recurrence as well as inaccurate reporting.

Supplementary Table S7. Studies that were excluded from the meta-analysis of primary prevention adjuvants.

Study	Reason for exclusion
Lee 2020	This study reported IUA incidence data collectively for all pathologies and did not specify what methodology was performed for resection of pathology. In addition, the study included patients with IUAs as the indication for their surgery, thus making it impossible to use these data for the purposes of de novo IUA formation.
Yu 2016	This study was retrospective.
Kim 2012	This is an RCT that evaluated prophylaxis of IUA post multiple different procedures for multiple pathologies without a non-adjuvant arm (CHA gel vs HA gel).

Supplementary Table S8. Summary data on the studies included in the meta-analysis of primary prevention adjuvants.

Study	Adjuvants	Event Adjuvant Arm	Population Adjuvant Arm	Event Control Arm	Population Control Arm	Procedure Subgroup
Sroussi 2022	Hyaluronic Acid Gel	13	144	24	134	Removal of Products of Conception (1st trimester)
Vatanatara 2021	ACH Gel + Antibiotic therapy	5	32	12	30	
Hooker 2017	ACP Gel	10	60	22	58	
Huang C-Y 2020	HA Gel, (Hyalobarrier® gel, Baxter, Pisa, Italy)	6	47	9	23	Hysteroscopic Myomectomy
DiSpiezio 2011	Intercoat Gel	1	16	3	15	
Guida 2004	ACP Gel	4	25	8	24	
Tafti 2021	Hyaluronic Acid Gel (Humedix Co., Korea) + Estrogen conjugate+ Medroxyprogesterone	4	34	12	31	Hysteroscopic Metroplasty for Septa
DiSpiezio 2011	Intercoat Gel	0	6	3	7	
Tonguc 2010	IUD + Estrogen	5	44	1	35	
Guida 2004	ACP Gel	1	8	3	8	

Supplementary Table S9. Studies excluded from the meta-analysis of secondary prevention adjuvants.

Study	Reason for exclusion
X Zhao 2022, Wu 2021, Zhao 2020, Salma 2020, Liu 2019, Sebbag 2019, Azumaguchi 2019, Zhu 2018, Chen 2017, Peng 2017, Yang 2016, Thubert 2015, Liu 2014, Yamamoto 2013, Yu 2008, Preutthipan 2000, Pabuccu 1997	These studies did not use the RCT design required for this analysis.
Amer 2010	This study used a randomized design however did not report on recurrence of adhesions, and only described severity using a non-standardized classification system
Trinh 2022	This study is retrospective and subjects were evaluated by HSG to determine IUA recurrence instead of by hysteroscopy as is required for analysis.
Lisa 2024, Pabuccu 2019, Guo 2017	This study used a randomized design however did not report on recurrence rate of IUAs, only the change in AFS scores.
Jin 2021	This study used a randomized design however it reported the use of “electroacupuncture with collecting ring method” to perform adhesiolysis for one of the study arms without a detailed description that would allow this study to be categorized alongside other techniques. In addition, this study was noted to have inadequate methodology for assessment of IUA recurrence as well as inaccurate reporting.
Qu 2021	This study used a randomized design however it contained internal errors in reporting and utilized an inadequate methodology for reporting recurrence.
Mao 2020	This study used a randomized design however it did not report a methodology for second look follow-up evaluation, and, in the methodology did not describe the required use of second look hysteroscopy for diagnosis and characterization of adhesions.

Supplementary Table S10. Summary data on studies included for the meta-analysis of secondary prevention adjuvants.

Study	Adjuvants	Event	Population	Procedure Subgroup
Hanstede 2023	Copper IUD with or without hormone therapy	30	114	IUD
Y Wang 2022	Uterine-Shaped Copper IUD _ Estradiol valerate x21 days + progesterone days 12-21, x2 cycles	26	43	
Wang Y.Q. 2020	Copper IUD + Hormone Therapy	19	35	
Lin 2015	Heart-Shaped Copper IUD x1 week + Estradiol valerate 6mg/day x21 days with medroxyprogesterone days 15-21 + Antibiotic Therapy	28	80	
Fernandez 2024	Womed Leaf film	42	71	Gel Barrier
Wang Y.Q. 2020	HA Gel (BioRegen Biomedical (Changzhou) Company Limited) + Hormone therapy	4	30	
Acunzo 2003	ACP Gel + Antibiotic Therapy	6	40	
Zhu 2024	Foley balloon catheter	23	71	Balloon
Guo 2022	Foley balloon x5-7 days + Estradiol 4mg/day x21 days + progesterone days 12-21, x2 cycles + Antibiotic Therapy	20	87	
Shen 2022b	Intrauterine-Suitable Balloon (ISB) + Hormone Therapy	18	60	
Wang S 2022	Foley balloon	7	15	
Yang 2022	Foley balloon with and without estrogen	65	182	
Y Wang 2022	Heart-Shapes Balloon Stent + Estradiol valerate x21 days + progesterone days 12-21, x2 cycles	22	41	
Pan L-Z 2021	Uterine balloon (Cook) x5 days + Danshen Intrauterine perfusion 3x/month x3 cycles + Oral Chinese Medicine + Estradiol valerate 3 mg/day x21 days + progesterone days 12-21, x3 cycles	16	35	
Zhou 2021	Foley balloon x5-7 days + Estradiol 4mg/day x21 days + progesterone days 12-21,	49	123	

	x2 cycles + Antibiotic Therapy			
Yang 2020	Intrauterine balloon (Cook Medical) alone, Intrauterine balloon (Cook Medical) + balloon stent stem and deflated balloon in situ for another 7 days, or intrauterine balloon (Cook Medical) + balloon stent stem and deflated balloon in situ for another 21 days	53	123	
X Huang 2020	Heart-shaped Balloon x1 week + Oestradiol valerate 4mg/day x21 days + Antibiotic Therapy	24	62	
Shi 2019	Foley balloon x5 days + Estradiol valerate 4mg/day x21 days + progesterone days 15-21, x2 cycles + Antibiotic Therapy	57	97	
Gan 2017	Foley Balloon x1 week + Estradiol valerate x21 days + progesterone days 12-21 + Antibiotic therapy	16	40	
Lin 2015	Heart-Shaped Balloon x1 week + Estradiol valerate 6mg/day x21 days with medroxyprogesterone days 15-21 + Antibiotic Therapy	25	82	
Xiao 2015	Foley Balloon x4 days	47	56	

Supplementary Table S11. Studies excluded from the meta-analysis of head-to-head comparisons of adjuvant therapies for secondary prevention.

Study	Reason for exclusion<br>In each study, the adjuvants were insufficiently similar to those in other studies, preventing pooling for meta-analysis.
Shen 2022	This is a randomized trial that compared plasma rich protein + intrauterine balloon vs intrauterine balloon alone.
Pan 2021	This is a randomized trial that compared intrauterine balloon + hyaluronic acid gel + IUD vs intrauterine balloon alone.
Yang 2020	This is a randomized trial that compared intrauterine balloon in place x7 days vs balloon in place x7 days and then left in situ deflated x7 days vs balloon in place x 7 days and then left in situ for 21 days.
Wang Y-Q 2020, Pabuccu 2019	Wang Y-Q 2020 is a randomized trial that compared hyaluronic acid gel vs hyaluronic acid gel + copper IUD vs copper IUD alone. Pabuccu 2019 is also a randomized trial that compared hyaluronic acid gel vs hyaluronic acid gel + Lippes Loop IUD vs Lippes Loop IUD alone. However, this study is ineligible for inclusion as it does not report on the IUA recurrence, only on the change in AFS scores and therefore, couldn't be pooled for meta-analysis.
Shi 2019	This is a randomized trial that compared intrauterine balloon x 5-7 days + balloon dilation at 2 weeks and 6 weeks vs intrauterine balloon x 5-7 days alone.
Huang X-W 2020, Qu 2021	Huang X-W 2020 is a randomized trial that compared intrauterine balloon x 3 days + IUD (unspecified) x 2-3 months vs intrauterine balloon x 7 days alone. Qu 2021 is also a randomized trial that compared intrauterine balloon x 7 days + GyneFix® copper IUD vs intrauterine balloon x 37 days alone; however, this study contained internal errors in reporting and utilized an inadequate methodology for reporting recurrence and thus ineligible for inclusion.
Zhu 2024	This is a randomized trial that compared autologous bone marrow stem cells + intrauterine balloon x 1 day + vaginal sildenafil vs intrauterine balloon x 3 days alone + vaginal sildenafil.

Supplementary Table S12. Studies excluded from the meta-analysis of obstetrical outcomes among patients following adhesiolysis.

Study	Reason for exclusion
Hong 2025	This study only reported total pregnancies, not live births.
Xiang 2024	The study objective was to analyze the impact of the time interval from adhesiolysis to pregnancy on placenta accreta spectrum on women with IUAs.
Xiong 2024, Xu 2024, Fouks 2022, Zhang 2021	These studies did not separately categorize the obstetrical outcomes by those patients who received adjuvant therapy compared to those who did not.
Jegaden 2023	This study reported solely on subjects that previously underwent uterine artery embolization with subsequent IUA, biasing the pregnancy outcome data.
Li, W 2020	This study reported on 10 patients with cervical insufficiency as well as IUA, biasing the pregnancy outcome data.
Sanad 2016	This prospective cohort study reported internally conflicting data regarding the live birth rate.
Kubinova 2012	This study reported on patients who underwent myomectomy with second-look hysteroscopy, only 1.56% had IUAs or underwent adhesiolysis.
Thomson 2007	This study reported on subjects that underwent fluoroscopically guided adhesiolysis; no direct visualization with hysteroscopy was performed.
Zikopoulos 2004	This retrospective study reported internally conflicting data regarding the live birth rate.

Supplementary Table S13. Summary data on studies included in the meta-analysis of obstetrical outcomes following hysteroscopic adhesiolysis with adjuvants.

Study	Adjuvants	Event	Population	Outcome
Yang H 2022	Uterine-shaped stainless-steel intrauterine device (copper coil, Yandai Contraceptive Instrument Company, China + HA gel)	2	33	Antepartum hemorrhage
Zhu 2022	Intrauterine balloon OR copper IUD	1	111	Antepartum hemorrhage
Yang H 2022	uterine-shaped stainless-steel intrauterine device (copper coil, Yandai Contraceptive Instrument Company, China + HA gel)	1	33	Placental Abruption
Song 2014	Foley +Hormone Therapy (Non-UAE group)	1	12	Placental Abruption
Tsui 2014	Hormone Therapy + balloon catheter + Hyalobarrier® gel	1	2	Placental Abruption
Wu 2024	Scissors Transection + Hormone + IUD, RF Transection Hormone + IUD, RF Transection + Hormone + HA Gel, and RF Transection + Hormone + IUD and HA Gel	200	493	Preterm labor / rupture of membranes (PROM)
Zhu 2022	Intrauterine balloon OR copper IUD	1	111	Preterm labor / rupture of membranes (PROM)
Yun Cao 2018	Stem cells onto foley balloon	2	8	Preterm labor / rupture of membranes (PROM)
Ding 2024	IUD x1 month + IV abx sulbenicillin and levornidazole + 2 cycles of estradiol for 20 days w/ dydrogesterone for last 10 days, IUD x 2 months + IV abx sulbenicillin and levornidazole + 2 cycles of estradiol for 20 days w/ dydrogesterone for last 10 days, and Balloon x 5 days + IV abx sulbenicillin and levornidazole + 2 cycles of estradiol for 20 days w/ dydrogesterone for last 10 days	12	58	Preterm delivery
Wu 2024	Scissors Transection + Hormone + IUD, RF Transection Hormone + IUD, RF Transection + Hormone + HA Gel, and RF Transection + Hormone + IUD and HA Gel	66	493	Preterm delivery
Zhu 2024	Estradiol 6mg daily x 40 days/progesterone x 10 days + BMSCs-scaffold (Autologous bone marrow stem cells-scaffold transplantation) + intrauterine balloon x 24 hrs + 3 days of levofloxacin antibiotics + vaginal sildenafil citrate x 5 days and estradiol 6mg daily x 40 days/progesterone x 10 days + intrauterine balloon x 72 hours + 3 days of levofloxacin antibiotics + vaginal sildenafil citrate x 5 days	6	68	Preterm delivery
Hansen 2022	Hyaluronic Acid Gel + IUD + Hormone Therapy	4	24	Preterm delivery
Zhu 2022	Intrauterine balloon OR copper IUD	3	111	Preterm delivery
Aghajanova 2021	PRP + Foley balloon + Antibiotics + estradiol 2mg BID for 30 days and MPA 10mg daily for the last 7 days and No Platelet-rich plasma; Foley Balloon + Antibiotics + estradiol 2mg BID for 30 days and MPA 10mg daily for the last 7 days	3	11	Preterm delivery
Chen 2020	Hormonal Therapy + heart shaped copper IUD + Foley catheter + 2 mL sodium hyaluronate gel	11	55	Preterm delivery

X Huang 2020	Heart-shaped Balloon + Oestradiol valerate + Antibiotic Therapy and IUD + Foley Balloon + Oestradiol valerate + Antibiotic Therapy	4	64	
Zhang 2019	Hormone Therapy + IUD	5	75	
Xu 2018	Hormonal Therapy OR IUD OR Foley Balloon OR HA gel OR None	7	80	
Yun Cao 2018	Stem cells onto foley balloon	2	8	
Liu 2014	Hormone Therapy + IUD + Antibiotic Therapy	2	49	
Xiao 2014	Hormonal therapy + IUD + Sodium hyaluronate gel	60	243	
Yu 2008	Hormonal therapy + IUD	4	25	
Preutthipan 2000	Hormonal therapy + IUD	1	18	
Zhu 2024	Estradiol 6mg daily x 40 days/progesterone x 10 days + BMSCs-scaffold (Autologous bone marrow stem cells-scaffold transplantation) + intrauterine balloon x 24 hrs + 3 days of levofloxacin antibiotics + vaginal sildenafil citrate x 5 days and estradiol 6mg daily x 40 days/progesterone x 10 days + intrauterine balloon x 72 hours + 3 days of levofloxacin antibiotics + vaginal sildenafil citrate x 5 days	3	68	Peripartum Hemorrhage
Yang H 2022	Uterine-shaped stainless-steel intrauterine device (copper coil, Yandai Contraceptive Instrument Company, China + HA gel)	3	33	
Aghajanova 2021	PRP + Foley balloon + Antibiotics + estradiol 2mg BID for 30 days and MPA 10mg daily for the last 7 days and No Platelet-rich plasma; Foley Balloon + Antibiotics + estradiol 2mg BID for 30 days and MPA 10mg daily for the last 7 days	2	11	
X Huang 2020	Heart-shaped Balloon + Oestradiol valerate + Antibiotic Therapy and IUD + Foley Balloon + Oestradiol valerate + Antibiotic Therapy	2	64	
Liu 2014	Hormone Therapy + IUD + Antibiotic Therapy	2	49	
Xiao 2014	Hormonal therapy + IUD + Sodium hyaluronate gel	51	243	
Yu 2008	Hormonal therapy + IUD	2	25	
Hansen 2022	Hyaluronic Acid Gel + IUD + Hormone Therapy	3	24	Retained placenta
Zhu 2022	intrauterine balloon OR copper IUD	1	111	
Chen 2020	Hormonal Therapy + heart shaped copper IUD + Foley catheter + 2 mL sodium hyaluronate gel	13	55	
X Huang 2020	Heart-shaped Balloon + Oestradiol valerate + Antibiotic Therapy and IUD + Foley Balloon + Oestradiol valerate + Antibiotic Therapy	2	64	
Song 2014	Foley +Hormone Therapy (Non-UAE group)	1	12	
Yu 2008	Hormonal therapy + IUD	3	25	
Zhu 2024	Estradiol 6mg daily x 40 days/progesterone x 10 days + BMSCs-scaffold (Autologous bone marrow stem cells-scaffold transplantation) + intrauterine balloon x 24 hrs + 3 days of levofloxacin antibiotics +	11	68	Accreta Spectrum (PAS)

	vaginal sildenafil citrate x 5 days and estradiol 6mg daily x 40 days/progesterone x 10 days + intrauterine balloon x 72 hours + 3 days of levofloxacin antibiotics + vaginal sildenafil citrate x 5 days			
Yang H 2022	Uterine-shaped stainless-steel intrauterine device (copper coil, Yandai Contraceptive Instrument Company, China + HA gel)	1	33	
Zhu 2022	intrauterine balloon OR copper IUD	2	111	
Aghajanova 2021	PRP + Foley balloon + Antibiotics + estradiol 2mg BID for 30 days and MPA 10mg daily for the last 7 days and No Platelet-rich plasma; Foley Balloon + Antibiotics + estradiol 2mg BID for 30 days and MPA 10mg daily for the last 7 days	1	11	
Chen 2020	Hormonal Therapy + heart shaped copper IUD + Foley catheter + 2 mL sodium hyaluronate gel	9	55	
X Huang 2020	Heart-shaped Balloon + Oestradiol valerate + Antibiotic Therapy and IUD + Foley Balloon + Oestradiol valerate + Antibiotic Therapy	2	64	
Xu 2018	Hormonal Therapy OR IUD OR Foley Balloon OR HA gel OR None	15	80	
Yun Cao 2018	stem cells onto foley balloon	0	8	
Liu 2014	Hormone Therapy + IUD + Antibiotic Therapy	2	49	
Song 2014	Foley +Hormone Therapy (Non-UAE group)	0	12	
Xiao 2014	Hormonal therapy + IUD + Sodium hyaluronate gel	64	243	
Yu 2008	Hormonal therapy + IUD	2	25	
Zhu 2024	Estradiol 6mg daily x 40 days/progesterone x 10 days + BMSCs-scaffold (Autologous bone marrow stem cells-scaffold transplantation) + intrauterine balloon x 24 hrs + 3 days of levofloxacin antibiotics + vaginal sildenafil citrate x 5 days and estradiol 6mg daily x 40 days/progesterone x 10 days + intrauterine balloon x 72 hours + 3 days of levofloxacin antibiotics + vaginal sildenafil citrate x 5 days	3	68	Placenta Previa
Hansen 2022	Hyaluronic Acid Gel + IUD + Hormone Therapy	1	24	
Yang H 2022	Uterine-shaped stainless-steel intrauterine device (copper coil, Yandai Contraceptive Instrument Company, China + HA gel)	2	33	
Zhu 2022	Intrauterine balloon OR copper IUD	7	111	
Aghajanova 2021	PRP + Foley balloon + Antibiotics + estradiol 2mg BID for 30 days and MPA 10mg daily for the last 7 days and No Platelet-rich plasma; Foley Balloon + Antibiotics + estradiol 2mg BID for 30 days and MPA 10mg daily for the last 7 days	1	11	
X Huang 2020	Heart-shaped Balloon + Oestradiol valerate + Antibiotic Therapy and IUD + Foley Balloon + Oestradiol valerate + Antibiotic Therapy	1	64	

Xu 2018	Hormonal Therapy OR IUD OR Foley Balloon OR HA gel OR None	2	80	
Liu 2014	Hormone Therapy + IUD + Antibiotic Therapy	3	49	
Tsui 2014	Hormone Therapy + balloon catheter + Hyalobarrier® gel	1	2	
Xiao 2014	Hormonal therapy + IUD + Sodium hyaluronate gel	12	243	
Yang H 2022	uterine-shaped stainless-steel intrauterine device (copper coil, Yandai Contraceptive Instrument Company, China + HA gel)	0	33	Hysterectomy
Chen 2020	Hormonal Therapy + heart shaped copper IUD + Foley catheter + 2 mL sodium hyaluronate gel	1	55	
Xu 2018	Hormonal Therapy OR IUD OR Foley Balloon OR HA gel OR None	0	80	
Liu 2014	Hormone Therapy + IUD + Antibiotic Therapy	1	49	
Yu 2008	Hormonal therapy + IUD	2	25	
Hansen 2022	Hyaluronic Acid Gel + IUD + Hormone Therapy	0	24	Still birth
Yang H 2022	uterine-shaped stainless-steel intrauterine device (copper coil, Yandai Contraceptive Instrument Company, China + HA gel)	1	33	
Chen 2020	Hormonal Therapy + heart shaped copper IUD + Foley catheter + 2 mL sodium hyaluronate gel	0	55	
Tsui 2014	Hormone Therapy + balloon catheter + Hyalobarrier® gel	0	2	
Preutthipan 2000	Hormonal therapy + IUD	0	18	

Supplementary Table S14. Summary data on studies included in the meta-analysis of obstetrical outcomes following hysteroscopic adhesiolysis without use of adjuvants.

Study	No adjuvant cohort	Event	Population	Outcome
Mortimer 2024	Various techniques (possible hormone, possible IU catheter)	13	76	IUGR
Hui 2018	No adjuvant	1	19	
Mortimer 2024	Various techniques (possible hormone, possible IU catheter)	7	76	Placental Abruption
Lu-Ping Zhang 2020	Hormone therapy	0	74	
Wu 2024	RF Transection + Hormone Only and Scissors Transection + Hormone Only	48	221	Preterm labor / rupture of membranes (PROM)
Deans 2018	Hormone therapy	1	88	
Capella 1999	Hormone therapy	1	9	
Mortimer 2024	Various techniques (possible hormone, possible IU catheter)	27	76	Preterm delivery
Wu 2024	RF Transection + Hormone Only and Scissors Transection + Hormone Only	38	221	
Lu-Ping Zhang 2020	Hormone therapy	5	74	
Deans 2018	Hormone therapy	28	88	
Hui 2018	No adjuvant	4	19	
Chen 2017	Hormone Therapy	5	140	
Capella 1999	Hormone therapy	1	9	
Mortimer 2024	Various techniques (possible hormone, possible IU catheter)	26	76	Peripartum hemorrhage
Lu-Ping Zhang 2020	Hormone therapy	16	74	
Deans 2018	Hormone therapy	13	88	
Chen 2017	Hormone therapy	11	140	
Fernandez 2012	No adjuvant	0	6	
Mortimer 2024	Various techniques (possible hormone, possible IU catheter)	28	76	Accreta Spectrum (PAS)
Lu-Ping Zhang 2020	Hormone therapy	17	74	
Deans 2018	Hormone therapy	7	88	
Chen 2017	Hormone Therapy	9	140	
Fernandez 2012	No adjuvant	0	6	
Capella 1999	Hormone therapy	2	9	

Mortimer 2024	Various techniques (possible hormone, possible IU catheter)	16	76	Placenta Previa
Lu-Ping Zhang 2020	Hormone therapy	6	74	Placenta Previa
Deans 2018	Hormone therapy	7	88	Placenta Previa
Hui 2018	No adjuvant	1	19	Placenta Previa
Deans 2018	Hormone therapy	4	88	Hysterectomy
Capella 1999	Hormone therapy	1	9	Hysterectomy
Lu-Ping Zhang 2020	Hormone therapy	0	74	Still birth
Deans 2018	Hormone therapy	3	88	Still birth
Fernandez 2012	No adjuvant	0	6	Still birth
Capella 1999	Hormone therapy	0	9	Still birth

Supplementary Table S15. Studies excluded from the meta-analysis of menstrual outcomes among patients following hysteroscopic adhesiolysis.

Study	Reason for exclusion
X Zhao 2022, Preutthipan 2000, Baradwan S 2018	These studies did not report on menstrual outcomes following adhesiolysis.
Pan L-Z 2021, Karande 1997	These studies did not report on the proportion of patients with normal menstruation at baseline.
H Huang 2020	This study was a retrospective analysis of patients who underwent repeat adhesiolysis with a patented intrauterine stent, which was a clear outlier among the patients studied.
Salma 2020, Yang 2020, Liu L 2019, Zhang L-P 2019, Cai 2017, Sanad 2016, Bhandari 2015, Hanstede 2015, Krajoviov 2015, Fernandez 2012, Roy 2010, Dawood 2009, Yu D 2008, Thomson 2007, Valle 1998	These studies did not use an RCT design, as was required for this analysis.
Chen L 2017	This study has internal inconsistencies within the written description of the results, which do not match those listed in Table 2 and Table 3.
Malhotra 2012	In this study, 6-month menstruation follow-up data were not categorized and are described vaguely.
Abiodun 2007, Efetie 2006, Orhue 2003	These studies were excluded as the patients did not undergo hysteroscopic evaluation at any point. They underwent blind adhesiolysis with a uterine sound.
Javaheri 2020	Though this study referred to itself as a randomized clinical trial in the title, the description of the methodology reflected that it was a non-randomized trial, thus led to this study's exclusion.
